# Fate and carbon sequestration potential of sunken macroalgae in coastal oceans from long-term microbial degradation perspective

**DOI:** 10.1093/nsr/nwaf273

**Published:** 2025-07-08

**Authors:** Hongmei Li, Zenghu Zhang, Jing Chen, Shailesh Nair, Tianqi Xiong, Hanshuang Zhao, Ding He, Kitack Lee, Nianzhi Jiao, Yongyu Zhang

**Affiliations:** Qingdao New Energy Shandong Laboratory, Qingdao Institute of Bioenergy and Bioprocess Technology, Chinese Academy of Sciences, Qingdao 266101, China; Qingdao New Energy Shandong Laboratory, Qingdao Institute of Bioenergy and Bioprocess Technology, Chinese Academy of Sciences, Qingdao 266101, China; College of Chemistry and Environment, Ankang University, Ankang 725000, China; Qingdao New Energy Shandong Laboratory, Qingdao Institute of Bioenergy and Bioprocess Technology, Chinese Academy of Sciences, Qingdao 266101, China; College of Environmental Science and Engineering, Qingdao University, Qingdao 266071, China; Qingdao New Energy Shandong Laboratory, Qingdao Institute of Bioenergy and Bioprocess Technology, Chinese Academy of Sciences, Qingdao 266101, China; Department of Ocean Science and Center for Ocean Research in Hong Kong and Macau, The Hong Kong University of Science and Technology, Hong Kong, China; Division of Environmental Science and Engineering, Pohang University of Science and Technology, Pohang 37673, Korea; Carbon Neutral Innovation Research Center and Fujian Key Laboratory of Marine Carbon Sequestration, Xiamen University, Xiamen 361101, China; Qingdao New Energy Shandong Laboratory, Qingdao Institute of Bioenergy and Bioprocess Technology, Chinese Academy of Sciences, Qingdao 266101, China; Southern Marine Science and Engineering Guangdong Laboratory (Zhuhai), Zhuhai 519782, China; Laboratory for Marine Biology and Biotechnology, Qingdao Marine Science and Technology Center, Qingdao 266237, China

**Keywords:** sunken macroalgae, ocean carbon sequestration, microbial degradation, recalcitrant organic carbon, bicarbonate production

## Abstract

Although deep-sea macroalgae sinking as a carbon sequestration strategy remains controversial, natural sinking of massive macroalgae frequently occurs in coastal oceans. In the Yellow Sea, millions of tons of the macroalga *Ulva prolifera* sink to the seafloor annually following green tides, yet their ultimate fate and carbon sequestration potential remain poorly understood. Microbial communities play a crucial role in decomposing organic matter and determining the fate of sunken macroalgae. Our 2-year simulated microbial degradation of *U. prolifera* revealed that approximately 38% of the carbon in sunken macroalgal biomass was ultimately sequestered in various forms. Of this retained carbon, 10% was transformed into dissolved inorganic bicarbonate ions, enhancing seawater alkalinity and contributing to inorganic carbon storage. Meanwhile, 28% was transformed into recalcitrant dissolved/particulate organic carbon and algal detritus, consisting of degradation-resistant compounds rich in humic-like substances, polycyclic aromatic hydrocarbons and highly aromatic compounds. Metagenomic analysis showed that these transformations were driven by a coordinated microbial succession from r-strategists to K-strategists, mediated by a microbial carbon pump and a ‘microbially driven alkalinity pump’. Our findings suggest that large-scale sinking of *U. prolifera* holds substantial potential for long-term ocean carbon sequestration, contributing to stable carbon pools in both organic and inorganic forms.

## Teaser

The massive sunken macroalgae following green tides holds substantial potential for ocean carbon sequestration, providing new insights into the carbon sequestration potential of wild macroalgae that settle in coastal environments.

## INTRODUCTION

As climate change accelerates, macroalgae mariculture, commonly known as seaweed farming, has gained global attention for its significant potential in carbon sequestration [1–3]. To further enhance the carbon sequestration capacity of macroalgae, a strategy has been proposed recently to intentionally sink cultivated macroalgae into the deep sea (i.e. deep-sea macroalgae sinking) [4], despite the loss of its economic value [5–7]. Interestingly, this approach mirrors natural events observed in coastal oceans [8], where large-scale sinking of macroalgae has occurred after frequent massive macroalgal blooms worldwide [9–11].

The occurrence of large-scale macroalgal blooms, such as *Ulva prolifera* green tides and *Sargassum* golden tides, have markedly increased over the past two decades [12]. They have serious negative impacts on coastal ecosystems by causing hypoxia and acidification, and have severely affected local ocean economies [9–11]. Currently, the main strategy to alleviate the negative impact of macroalgal blooms, such as *U. prolifera* green tide in the Yellow Sea of China, is to intercept and salvage *U. prolifera* in the outer sea areas to prevent them from accumulating and rotting on the coastline. However, only a small part of the macroalgae can be salvaged by humans. During the decline stage of macroalgal blooms

(i.e. late August), most of the floating macroalgae sink to the seabed within a few days and disappear from the sea surface. Approximately 5 million tons of *U. prolifera* (fresh weight, *fw*) sank onto the seafloor following annual green tide events [13–15]. Theoretically, the massive sunken macroalgae should significantly contribute to carbon sequestration. However, the magnitude of their contribution depends on how much carbon derived from sunken macroalgae can be stored in the ocean in the long term. This remains unknown and should be determined by examining the form and stability of the residual carbon from macroalgae following thorough degradation.

As primary decomposers in the ocean and key drivers of marine biogeochemical cycles, microbial communities play a crucial role in regulating the degradation process and the fate of macroalgal-derived carbon sunk in the seafloor [16]. During macroalgal degradation, microorganisms can convert macroalgal biomass carbon into various components, mainly particulate organic carbon (POC), dissolved organic carbon (DOC) and dissolved inorganic carbon (DIC). These carbon forms exhibit varying degrees of biological and chemical stability. Most of the labile organic carbon forms are readily mineralized and respired by microorganisms, and thus only have a short residence time in the ocean. In contrast, recalcitrant organic carbon can resist microbial degradation and be stably stored in the ocean, playing a vital role in long-term carbon sequestration [16–18]. Among the various forms of DIC, including CO_3_^2^^−^, H_2_CO_3_, HCO_3_^−^, and CO_2_, bicarbonate ions constitute the predominant contributor to ocean alkalinity. Notably, bicarbonate ions show minimal conversion to CO_2_, thereby serving as stable inorganic carbon storage [19–21]. Although the degradation of macroalgal biomass involves complex interconversions among various organic and inorganic carbon forms, it is considered that as long as the final degradation products exhibit longevity in carbon storage, they meet the criteria for long-term carbon sequestration in the ocean, either buried in sediments, dissolved in seawater, or exported to the deep ocean.

The complex degradation process of macroalgae is orchestrated by a diverse array of microbial community members, metabolic pathways and interactions that change dynamically [22,23]. These changing microbial consortia and their metabolic capabilities ultimately shape the long-term persistence of carbon in the ocean by mineralizing labile fractions while simultaneously generating refractory metabolites resistant to further degradation. These refractory metabolites, synthesized by microorganisms from labile precursors, form a significant fraction of the ocean's huge recalcitrant organic carbon pool and are well described by the ‘microbial carbon pump’ theory [24]. Additionally, macroalgae inherently contain recalcitrant components (e.g. fucoidan and polyphenols) that are not easily degraded by microorganisms, thus contributing to ocean carbon sequestration [23]. However, it remains unclear how microbial communities regulate the degradation of macroalgal biomass and drive its transformation into recalcitrant carbon that can persist in the ocean over the long term.

It is essential to recognize that the fate of sunken macroalgal carbon in natural environments is influenced by various factors, including the grazing by herbivores [25,26] and complex environmental conditions (e.g. light availability, dissolved oxygen levels and hydrodynamic forces) [27–29]. The ultimate fate and carbon sequestration potential of macroalgal biomass can vary significantly [30]. However, monitoring this process *in situ* remains a major challenge. In this study, we excluded other influences and focused on investigating the impact of microbial degradation on the fate of sunken macroalgae and its carbon sequestration effect. To better understand this process, we conducted a 2-year controlled experiment simulating the long-term microbial degradation of *U. prolifera*. Our aim was to uncover the potential long-term fate and carbon sequestration of macroalgal biomass that sinks to the coastal seabed. Furthermore, we explored the role and mechanism of microbial communities in the long-term degradation of sunken macroalgae. The results provide valuable insights into the carbon sequestration potential of wild macroalgae that settle in coastal environments.

## RESULTS

### Long-term macroalgal degradation process revealed by changes in DOC release and microbial abundance

**Figure 1.**
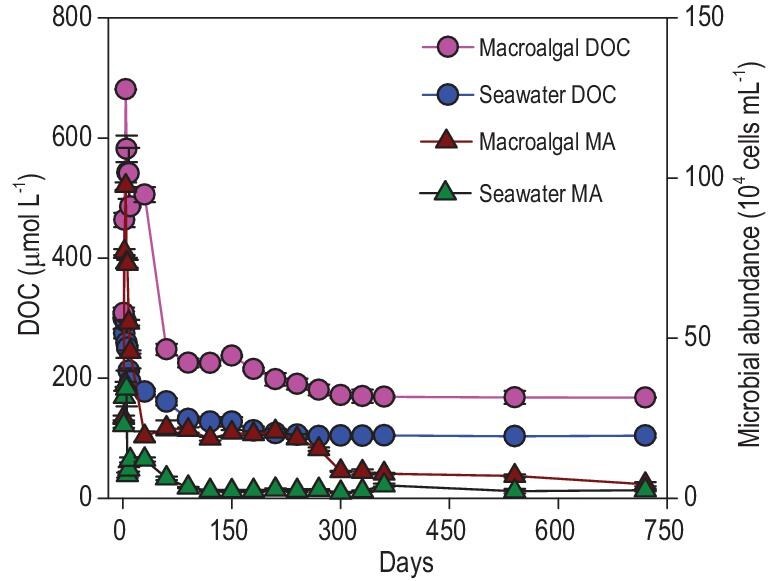
Dynamic changes in dissolved organic carbon (DOC) concentration and microbial abundance (MA) in the treatment (macroalgal DOC and macroalgal MA) and control (seawater DOC and seawater MA) groups during the long-term degradation process.

We conducted a 2-year microbial decomposition of *U. prolifera* in the laboratory to simulate the long-term degradation of sinking macroalgae. During the degradation process (Days 0–720), substantial changes in DOC concentration and microbial abundance occurred within the first 330 days (Fig. 1), and then kept stable in the remaining 390 days. In detail, an initial rapid degradation phase occurred within the first 4 days, as evidenced by a spike in DOC release, with concentrations increasing from 309 (±2) to 681 (±1) μM, accompanied by a proportional increase in microbial abundance from 3 × 10^5^ to 10 × 10^5^ cells mL^−^^1^. Subsequently, DOC concentration decreased to 170 ± 11 μM) and microbial abundance decreased to 8 × 10^4^ cells mL^-1^ decreased, with an initial rapid (Days 5–60), followed by a slower decline (Days 60–330). During Days 330–720, the DOC ceased to decrease and showed an antidegradation ability to microorganisms.

**Figure 2.**
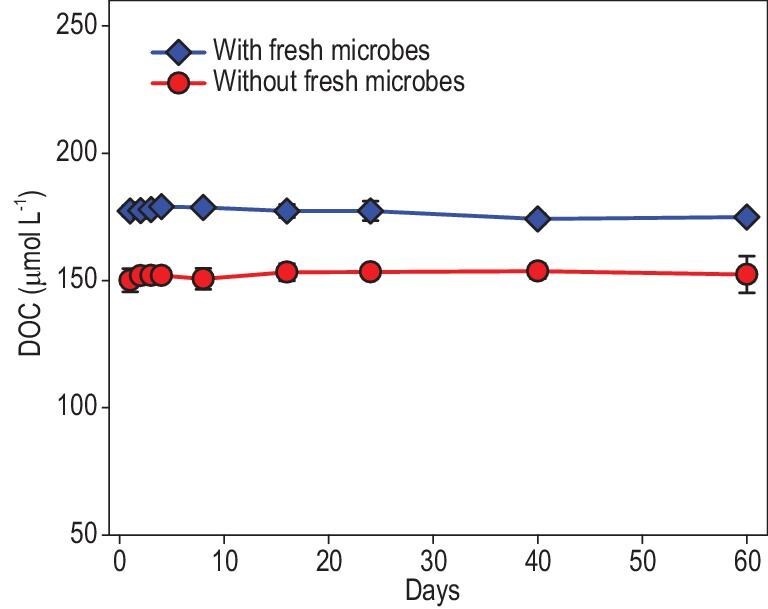
Dynamic changes in DOC concentration in the groups with and without fresh microorganisms during the 60-day follow-up degradation experiment.

After 720 days of microbial degradation, we further tested the resistance of the remaining DOC to microbial degradation by exposing it to a new microbial community introduced from coastal natural seawater for another 60 days. As a result, DOC concentrations remained stable in treatments both with (coefficient of variation = 0.9%) and without (coefficient of variation = 0.8%) fresh inoculum, indicating negligible degradation by the new microbial community and the inherent inert property (Fig. 2).

### Increasing recalcitrance properties of macroalgal degradation products evidenced by changes in DOC composition

**Figure 3.**
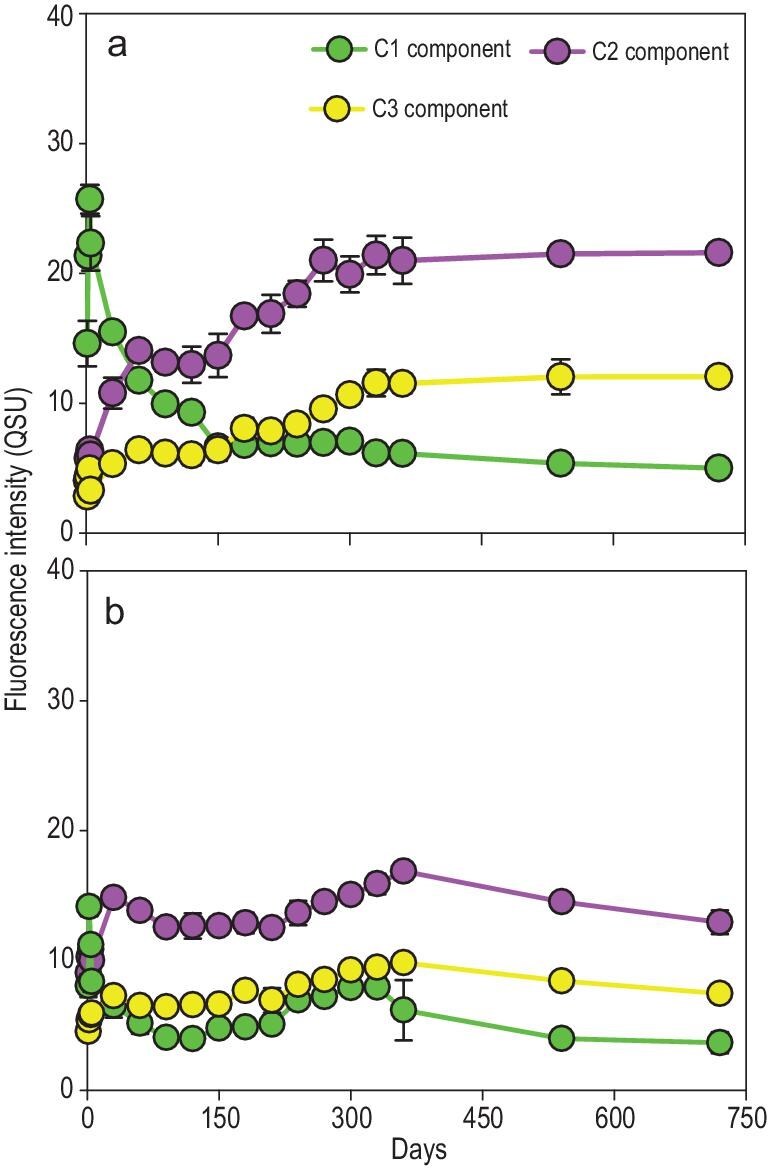
Dynamic changes in fluorescent dissolved organic matter (FDOM) components (C1, C2 and C3) during the long-term degradation process. (a) Treatment group; (b) control group.

Fluorescent components are optically sensitive compositions of organic matter and are often used as proxies for organic carbon [31]. In macroalgal degradation experiments, three main components of the fluorescent dissolved organic matter (FDOM) were identified: C1 [ex 225 (275)/em 340, protein-like substance], C2 [ex 250 (350)/em 420, humic-like substance] and C3 [ex 275 (370)/em 490, humic-like substance] (Supplementary data, Table S1). Similar to DOC concentrations, major variations in these FDOM components occurred within the first 330 days (Fig. 3a). However, protein-like and humic-like substances exhibited contrasting characteristics. The protein-like component (C1) showed a rapid initial increase for the first 4 days, followed by a continuous decrease from Days 5–330. Conversely, the two humic-like components (C2 and C3) gradually accumulated until Day 330 and then remained stable until the end of the degradation experiment. In the controls, the three components of FDOM showed similar patterns, but their amplitudes were much smaller than in the treatment group (Fig. 3b).

Meanwhile, Fourier transform ion cyclotron resonance mass spectrometry (FT-ICR MS) was used to analyze the molecular compositions of DOC. FT-ICR MS analysis identified 5026 distinct molecular formulae (MFs) of the DOC, classified into CHO-, CHNO-, CHOS- and CHNOS groups based on their elemental compositions. We found that the total MFs in the treatments increased by 7.2% (4356 vs 4063) on Day 720 compared to Day 1, with enrichment of CHOS (844 vs 737) and CHNOS (358 vs 299) containing MFs (Table 1). In contrast, CHO (1390 vs 1596) and CHNO (1764 vs 2070) containing MFs decreased on Day 720 compared to Day 1. Moreover, compared to initial DOC molecules on Day 1, the remaining DOC molecules on Day 720 (either in the control group or in the treatment group) exhibited increased recalcitrant properties, as indicated by the higher molecular mass (402 vs 388), higher O/C ratio (0.56–0.58 vs 0.54), higher double bond equivalent (DBE) value (8.17–8.25 vs 7.25), higher aromaticity index (AI*) value (0.24–0.25 vs 0.21) and a higher proportion of recalcitrant components divided by molecular lability boundary (MLB_R_) (89%–90% vs 76%) but lower H/C ratio (1.22 < 1.31) (Table 2) [32,33]. Notably, these recalcitrant properties were most pronounced in the treatment group, surpassing the control group on Day 720. Additionally, polycyclic aromatics and highly aromatic compounds, markers of recalcitrance [34], were enriched in the remaining macroalgal DOC (5.36%) compared to the initial DOC (2.64%) (Fig. S1).

**Table 1.** Elemental composition of DOC molecules in the treatment and control groups on Days 1 and 720 of the long-term degradation.

	Treatments		Controls	
Molecular composition	Day 1	Day 720	Day 1	Day 720
Total formula	4063	4356	4063	4702
CHO	1302	1390	1302	1596
CHNO	1604	1764	1604	2070
CHOS	851	844	851	737
CHNOS	306	358	306	299

**Table 2.** Molecular characteristics of DOC for all total molecular formulae in the treatment and control groups on Days 1 and 720 of the long-term degradation: peak magnitude weighted averages (wa) of the molecular mass (Mass_wa_), oxygen-to-carbon ratio (O/C_wa_), hydrogen-to-carbon ratio (H/C_wa_), double bond equivalent (DBE_wa_) and aromaticity index (AI*_wa_); percentage of labile (MLB_L_) and recalcitrant (MLB_R_) components divided by molecular lability boundary.

	Treatments		Controls	
Molecular characterization	Day 1	Day 720	Day 1	Day 720
Mass_wa_	388	402	388	402
O/C_wa_	0.54	0.58	0.54	0.56
H/C_wa_	1.31	1.22	1.31	1.22
^a^DBE_wa_	7.25	8.25	7.25	8.17
^a^AI*_wa_	0.21	0.25	0.21	0.24
^b^MLB_L_ (%)	24	10	24	11
MLB_R_ (%)	76	90	76	89

a DBE and aromaticity index (AI*) were calculated for all assigned DOC molecular formulae identified during the FT-ICR MS analysis. The DOC molecule with higher DBE and AI* values has more double bonds and higher aromaticity, indicating it is more recalcitrant to microbial degradation. ^b^MLB is used to divide the DOC molecules into more labile or recalcitrant constituents. The DOC constituents above the MLB at H/C ≥ 1.5 correspond to more labile material (MLB_L_), whereas DOC constituents below the MLB at H/C < 1.5 are indicative of more recalcitrant substances (MLB_R_). Here, the percentages of MLB_L_ and MLB_R_ are determined by the proportions of molecular formulae having H/C ≥ 1.5 or H/C < 1.5 in the total DOC molecules.

### Successive microbial communities driving the long-term degradation of macroalgae

During the macroalgal degradation, both particle-attached (3.0–20 μm) and free-living (0.2–3.0 μm) microbial communities exhibited similar successional changes (Figs S2 and S3). At the early degradation stage (Day 5), the initially dominant Proteobacteria phylum was replaced by Bacteroidetes in the treatment group compared to controls. At the family level, Flavobacteriaceae (belonging to phylum Bacteroidetes) and Rhodobacteraceae (belonging to phylum Proteobacteria) were the most dominant families. By Day 30, Proteobacteria and Bacteroidetes declined as Planctomycetes (particularly Rubinisphaeraceae) became the dominant taxa. From Day 60 to Day 120, Proteobacteria, Bacteroidetes and Planctomycetes persisted as the top three phyla. At the family level, Rubinisphaeraceae decreased substantially, but the relative abundance of Gimesiaceae and Pirellulaceae of the phylum Planctomycetes increased, with Gimesiaceae becoming the most dominant family in the free-living microbial communities. Concurrently, Chloroflexi proliferated in the particle-attached communities. From Day 180 to Day 330, Acidobacteria emerged and even surpassed Bacteroidetes in particle-attached microbial communities, becoming one of the top three dominant phyla alongside Proteobacteria and Planctomycetes. Additionally, in free-living microbial communities, Chloroflexi (mainly composed of Anaerolineaceae) and Acidobacteria also increased, with Pirellulaceae becoming the predominant family. At the end (Day 720), Actinobacteria, Thaumarchaeota and Firmicutes exhibited relative increases, especially in free-living microbial communities, where Thaumarchaeota became the second most dominant phylum. The corresponding emerging families were Nitrosopumilaceae (Thaumarchaeota), Nocardiaceae (Actinobacteria) and Ruminococcaceae (Firmicutes) in the particle-attached microbial community, and Nitrosopumilaceae (Thaumarchaeota) in the free-living microbial community.

### Microbial metabolic functions underpinning the changes in DOC composition released during long-term macroalgae degradation

A total of 11 323 functional genes across 304 KEGG pathways were identified (Table S2). Genes encoding for monosaccharide (glucose, fructose and galactose) catabolism, polysaccharide (starch and sucrose) metabolism, glycan and fatty acid metabolism were significantly enriched [adjusted *P* value (*P*_adj_) < 0.05) on Day 5, but their relative abundance had decreased significantly on Day 720 (Fig. S4). In contrast, genes involved in the degradation pathways of steroid, benzene-containing compounds, chlorocyclohexane and chlorobenzene, chloroalkane and chloroalkene, and polycyclic aromatic hydrocarbons (PAHs) were significantly (*P*_adj_ < 0.05) enriched by Day 720. Additionally, genes responsible for the synthesis of complex metabolites, including terpenoids, aromatic amino acids, glyoxylate and dicarboxylate, were also significantly enriched (*P*_adj_ < 0.05) by Day 720. This shift in metabolic function from initial simple carbohydrate utilization to complex organic degradation mirrors the enhanced recalcitrance of residual organic carbon after long-term macroalgal degradation. Given the rich content of sulfated polysaccharide ulvan in the cell walls of *U. prolifera*, we characterized ulvan degradation dynamics by analyzing the relative abundance of microbial functional genes encoding ulvan lyase (catalyzing ulvan to oligo-ulvan conversion), glucuronyl hydrolase (converting oligo-ulvan to l-rhamnose-3-sulfate), sulfatase and rhamnosidase (degrading l-rhamnose-3-sulfate). The consistent dynamics of these genes’ relative abundance indicated that ulvan degradation primarily occurred in the initial stage (e.g. on Day 5) (Fig. S5). Furthermore, metagenome-assembled genomes (MAGs) analysis revealed an abundance of genes encoding the mevalonate pathway and the methyl erythritol phosphate/1-deoxy-d-xylulose-5-phosphate (MEP-DOXP) pathway (Fig. S6). These pathways are essential for the generation of precursors of carboxyl-rich alicyclic molecules (CRAM) analogs, indicating that complex CRAMs were continuously synthesized by microbial communities and accumulated during long-term macroalgal degradation. This reflects the important role of microbial communities in the transformation of macroalgae-derived organic carbon from labile to recalcitrant, highlighting the microbial carbon pump function, as CRAMs are important markers of bio-recalcitrant DOC (RDOC) composition.

**Figure 4.**
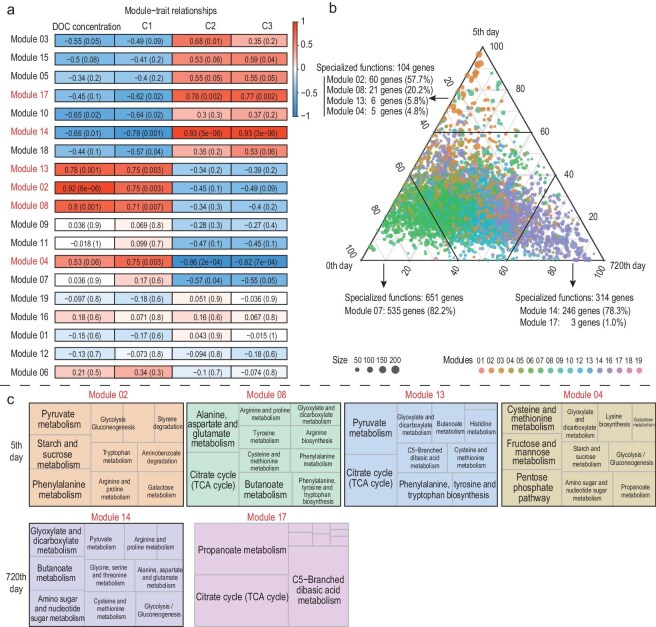
Changes in microbial community function during the long-term degradation process and its relationship with DOC concentration and FDOM components. (a) Relationships between gene modules and DOC concentration and FDOM components (C1, C2 and C3). Pearson’s correlation coefficient with *P* value in parentheses is indicated in the heatmap (red, positively correlated; blue, negatively correlated). (b) Ternary plots visualizing the distribution of function genes (*n* = 6024) on Days 0, 5 and 720 of the long-term degradation. Colors refer to gene modules identified by weighted gene co-expression network analysis (WGCNA). Functions were classified as being specialized to a certain timepoint when their relative abundance was greater than 60% (*n* = 1069). (c) Metabolic functional composition (including carbohydrate, amino acid and xenobiotics metabolisms) of gene modules that were significantly related to FDOM components. The top 10 metabolic functions in relative abundance are listed and the top 3 are marked in bold.

Moreover, weighted gene co-expression network analysis (WGCNA) clustered 11 323 functional genes into 19 modules associated with macroalgae-derived DOC composition at different degradation stages (Days 1, 5 and 720) (Table S3). Modules 02, 04, 08 and 13, primarily active in the early stage (Day 5) involving starch, sucrose, fructose, mannose and amino acid (phenylalanine, alanine, aspartate, glutamate, cysteine and methionine) metabolism, were significantly correlated with the released DOC concentration and protein-like fluorophore C1 (Pearson’s *r* > 0.71, *P* < 0.01, Fig. 4a). Conversely, modules 14 and 17, which peaked at the end (Day 720) and are involved in functions related to the carboxyl-rich compound metabolism (such as glyoxylate, dicarboxylate, butanoate propanoate and C5-branched dibasic acid), were significantly correlated with the humic-like components C2 or C3 (Pearson’s *r* > 0.53, *P* < 0.05) (Fig. 4b and c). These coordinated changes in microbial function genes and organic carbon compositions throughout the macroalgal degradation process result in the final degradation products being highly recalcitrant.

### The fate of macroalgal biomass carbon in different forms performing carbon sequestration function after long-term microbial degradation

The moisture content of *U. prolifera* thalli was 93.1% (±0.06%), with an initial carbon content of 33.5% (±3.1%) (dry weight). In the long-term degradation experiment, the macroalgal biomass carbon in each tank was calculated as 1920 (±26) μM prior to degradation. After the long-term degradation, 38.2% of the macroalgal biomass carbon (after deducting the controls without macroalgae) were retained in different forms, including DOC (63.2 ± 1.9 μmol), POC (426.7 ± 3.2 μmol), detritus carbon (48.9 ± 0.5 μmol), and DIC (195.7 ± 9.4 μmol), accounting for 3.3%, 22.2%, 2.5% and 10.2% of the initial macroalgal biomass carbon, respectively (Table S4).

As previously mentioned, all the residual organic carbon after long-term macroalgal degradation shows strong resistance to biodegradation, thus indicating its potential for stable storage in the ocean. In addition to the evidence from molecular composition analysis of the residual DOC and their anti-degradation capability, the remaining POC showed an enrichment of humic-like substances, an indicator of recalcitrant POC (Fig. S7) [31]. Moreover, the residual macroalgal detritus exhibited higher levels of phenolic, humic and condensed aromatic compounds (peaks D, F and G in Fig. S8 and Table S5) compared to the initial *U. prolifera* thalli. These are typical recalcitrant substances that make residual detritus difficult for microorganisms to degrade. The remaining DIC was also very stable, with approximately 90% of the residual DIC existing in the form of HCO_3_^−^ and 5% in the form of CO_3_^2^^−^, both of which are major contributors to seawater total alkalinity and have potential for inorganic carbon sequestration.

## DISCUSSION AND CONCLUSIONS

Under climate change scenarios, macroalgae have attracted wide attention due to their significant carbon sequestration capabilities through various pathways, such as the burial of macroalgal detritus or particulate organic carbon in local sediments, export to the deep ocean, and contribution of RDOC to seawater [35–37]. Since farmed macroalgae are harvested and removed from the ocean after maturity for various uses such as food or materials, their carbon sequestration primarily occurs during the macroalgal growth period [32]. In contrast, wild macroalgae contribute to carbon sequestration throughout their entire lifecycle, including after death, when their biomass sinks to the seafloor. In recent decades, large-scale macroalgal blooms have become more frequently globally [38]. The world's largest *U. prolifera* green tides in the Yellow Sea have occurred annually for 18 consecutive years, resulting in millions of tons of macroalgal biomass sinking to the seafloor each year [35,36]. These sunken macroalgae are eventually decomposed, particularly by microorganisms over long-term timescales. Unveiling the ultimate fate of this sunken biomass carbon of *U. prolifera*, especially the amount and forms of macroalgae-derived carbon that can be retained in the ocean after long-term microbial degradation, is crucial to understanding its carbon sequestration capacity.

By investigating the dynamic changes in DOC release and microbial abundance during 2 years of *U. prolifera* degradation under dark conditions (Fig. 1), we revealed that *U. prolifera* biomass could be completely degraded within approximately 1 year (∼330 days). During this period, most of the macroalgae-derived DOC was utilized by microorganisms and supported the growth of microbial communities. Therefore, this portion of the DOC was classified as bio-labile DOC (LDOC). In the second year (Days 330–720), the remaining DOC resisted microbial degradation, remained stable, and was thus categorized as RDOC. FDOM analysis revealed that protein-like substance (C1) decreased steadily within 5–330 days, indicating its bio-labile nature. In contrast, the continuous accumulation of humic-like components (C2 and C3) suggests that these were newly formed RDOC components generated through microbial degradation of the macroalgae-derived LDOC (Fig. 3). Additionally, the increase in the inertness index of the remaining DOC (including AI*, DBE and MLB_R_, Table 2), along with the enhanced presence of recalcitrant molecules (Fig. S1), suggests that the organic carbon left after long-term degradation is highly inert and has the potential to be stored in the ocean for a long time.

To further explore the recalcitrance of the remaining DOC, it was reinoculated with microbial communities from natural seawater and subjected to an additional 60 days of degradation, during which only 1.3% of the remaining DOC was further removed (Fig. 2). This minimal degradation suggests that the residual DOC after long-term macroalgal degradation is intrinsically inert and could contribute to long-term carbon sequestration.

**Figure 5.**
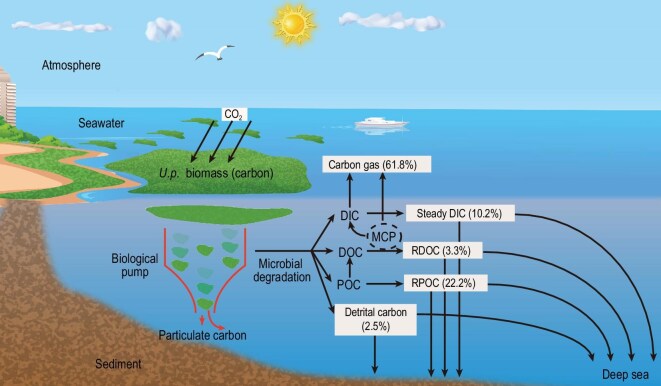
Potential fate of macroalgal (*U. prolifera*) biomass carbon after long-term microbial degradation. MCP, microbial carbon pump.

Two years of microbial degradation finally left behind ∼38.2% of the macroalgal biomass carbon existing in various forms (Fig. 5), including both organic (∼28%) and inorganic carbon (∼10.2%). This means that ∼60% of the macroalgal biomass carbon returned to the atmosphere, serving only as temporary carbon fixation rather than long-term carbon sequestration. The carbon sequestration capability of the residual macroalgae-derived carbon (i.e. DOC, POC, detritus carbon and DIC) depends on their stability and recalcitrance.

Regarding organic carbon stability, during the second year of long-term degradation, DOC concentrations and microbial abundance remained unchanged, and the remaining DOC showed strong resistance to further degradation by outside natural microbial communities. This indicates the strong intrinsic recalcitrance of remaining DOC and suggests that the residual macroalgal detritus and POC were difficult to decompose, as no increase in DOC was observed during this period.

Regarding recalcitrant markers, the fluorescent components and molecular composition characteristics of the residual macroalgae-derived organic carbon (including DOC, POC and detritus carbon) showed an enrichment of recognized recalcitrant markers (Figs S1, S7 and S8; Table S5), such as humic-like substance, polycyclic aromatics and highly aromatic compounds [22,39]. This further proves their intrinsic inertness and strong stability.

Regarding DIC stability, approximately 95% of the residual DIC exists as bicarbonate (HCO_3_^−^) and carbonate (CO_3_²⁻), which constitute the primary components of total alkalinity and show significantly greater stability in seawater than dissolved CO_2_. The stability of HCO_3_^−^ ions in ocean environments can be predicted using seawater carbonate thermodynamics in the context of ocean warming. Under ambient ocean conditions, HCO_3_^−^ ions are the most abundant among the three carbon species. For every unit of ocean temperature increase, < 0.5% of the total amount of HCO_3_^−^ ions in seawater can be converted into CO_2_, which could eventually be lost to the atmosphere. Recently, seawater alkalinity enhancement has been considered a potential way to increase ocean inorganic carbon sequestration [20,21,40].

From the perspective of microbial degradation, ∼38% of the initial macroalgal biomass carbon can eventually be retained after conversion to recalcitrant organic carbon and stable inorganic carbon, which have the prerequisites for long-term isolation from the atmosphere; we believe that sinking macroalgae have a considerable carbon sequestration contribution. Among the retained macroalgae-derived carbon after long-term degradation, a substantial portion of the recalcitrant POC (RPOC) or detritus carbon may be exported or reach the deep sea, in addition to being buried in local sediments. An estimate suggests that only 4%–9% of annually released macroalgal POC is sequestered within continental shelf sediments [41]. However, ∼18% of macroalgal biomass is transported as POC beyond the continental shelf despite high microbial activity within the shelf waters [35]. We speculate that these macroalgae-derived POCs exported to far distances or the deep sea should also be mainly RPOC after being degraded by microorganisms during the long-distance and long-time transportation, thus contributing to long-term carbon sequestration. Additionally, a considerable portion of the macroalgae-derived RDOC or stable DIC left behind may also be exported to the outside or reach the deep sea. For instance, the significantly enhanced stable DIC (mainly total alkalinity including bicarbonate and carbonate) and RDOC in the local coastal water due to microbial degradation of macroalgal carbon during or after the green tide demise period usually declined to its original level within a certain period, probably due to their outward output [21]. Similarly, the outward export of stable inorganic carbon (e.g. total alkalinity) has also been demonstrated in mangrove systems [20].

We also revealed the microbial communities and functions underpinning the long-term macroalgal degradation and the conversion between different carbon forms. During long-term macroalgal degradation, microbial communities and functions dynamically adjusted in response to changes in the compositions and properties of macroalgae-derived organic carbon at different stages. In the early stage, microorganisms mainly decompose labile components in macroalgal biomass and rapidly utilize the released LDOC, evidenced by the rapid release of DOC and protein-like fluorescent components and the subsequent rapid decrease in their concentrations (Fig. 1, Fig. 3). As the labile components are gradually consumed, the remaining macroalgae-derived organic carbon components become increasingly inert, which can be proved by the increase in the inertness index (including AI*, DBE, MLB_R_, Table 2) of the remaining DOC and the enhancement of the proportion of inert components (e.g. humic-like, phenolic and condensed aromatic compounds, Figs S7 and S8; Table S5) in POC and macroalgal detritus. These residual recalcitrant organic carbons should include the difficult-to-degrade components originally contained in the macroalgae [14,42], and the inert products (also including inert microbial necromass) formed by microorganisms after degrading the macroalgal labile components [22,43]. In response to the overall labile-to-recalcitrant transformation of organic carbon, the microbial community shifted gradually from r-strategists to K-strategists.

The initial rapid degradation of macroalgal biomass was facilitated by opportunistic r-strategist microorganisms like Proteobacteria and Bacteroidetes [44,45], releasing a surge of LDOC fractions (e.g. protein-like fluorophore, saturated fatty and sulfonic acids). r-Strategists are efficient at processing LDOC due to their rapid growth and high metabolic rates. Many Bacteroidetes members possess specialized polysaccharide utilization loci (PULs) evolved for degrading complex algal carbohydrates such as polysaccharides and glycans and are usually found in high abundance during algal blooms [46]. Concurrently, co-expression network analysis and differential gene abundance revealed enrichment in the genes involved in polysaccharide, glycan and fatty acid metabolism. As the initial colonizers degraded the macroalgal cell wall, it released a surge of other labile carbons including sulfur-containing molecules (Table 1), which gave rise to secondary colonizers, particularly Planctomycetes along with Bacteroidetes. Cell walls of *U. prolifera* are rich in sulfated polysaccharide ulvan [22]. Certain members of Planctomycetes (Rubinisphaeraceae, Gimesiaceae and Pirellulaceae) can secrete sulfated polysaccharide-degrading enzymes such as sulfatases, which might have been advantageous for their rise [47–49].

As labile fractions were depleted, a shift towards a specialized K-strategist community, dominated by Planctomycetes, Chloroflexi, Acidobacteria, Actinobacteria and Thaumarchaeota, occurred [45]. Members of Acidobacteria were reported to be able to degrade difficult-to-utilize organic matter such as cellulose, chitin and xylan [50]. The emergence of Thaumarchaeota, known for their role in ammonia oxidation and release of HCO_3_^−^, suggests an important pathway for stable DIC production during macroalgal degradation [51]. In general, K-strategists are efficient in processing complex organic carbons and could grow under limited availability of biodegradable organic carbon leading to the generation of RDOC molecules [52]. This was strongly reflected by the successional changes in FDOM components, with the gradual depletion of protein-like substances and continuous enhancement of humic-like substances suggesting a conversion of LDOC to RDOC [22,53]. The positive correlations between recalcitrant humic-like components and the related microbial gene modules involved in degrading complex aromatic compounds, steroids and chlorinated hydrocarbons, and biosynthesizing CRAMs on Day 720 further reinforce the pivotal role of these microorganisms in generating refractory carbon. Such humic-like CRAMs have been reported to be a major fraction of the oceanic RDOC pool [54]. Here, the coordinated community succession and generation of structurally complex RDOC molecules corroborate the ‘microbial carbon pump’ theory, which posits that microorganisms continuously rework and modify organic matter, generating refractory metabolites resistant to further degradation in the ocean.

It was unexpected that such a large amount of stable inorganic carbon (or total alkalinity as predominantly bicarbonate) was produced during microbial processing of labile POC and DOC, accounting for 10.2% of the initial macroalgal biomass carbon. We tentatively call this microbially driven alkalinity enhancement activity the ‘microbially driven alkalinity pump,’ a microbially driven mechanism for inorganic carbon sequestration. In other words, microbial processes can increase seawater alkalinity, enhancing bicarbonate production via various processes including sulfate reduction and denitrification [16,55,56]. Specifically, during denitrification, nitrate (a strong acid anion) serves as an electron acceptor for organic matter oxidation, with nitrate ultimately reduced to nitrogen gas that is irreversibly lost to the atmosphere. The negative charge associated with nitrate is transferred to bicarbonate, a weak acid anion, thereby increasing seawater alkalinity. In sulfate reduction, sulfate is reduced to sulfide, which reacts with metal cations to form insoluble sulfide minerals that are irreversibly lost. This process transfers the negative charge of sulfate to bicarbonate while removing acidic sulfide species from the water column, resulting in a net gain in alkalinity [55]. Indeed, our microbial functional profiles revealed enrichment in genes regulating these processes (Table S3, e.g. K00958 encoding sulfate adenylyltransferase in the sulfate reduction pathway; K00370–K00371 encoding nitrate reductase in the denitrification pathway), concurrent with increased stable bicarbonate production. These transformations are intrinsically linked to changes in seawater pH, as evidenced by our results. At the early degradation stage, microbial respiration/mineralization of macroalgae-released organic carbon produced CO_2_ and lowered pH of the seawater, which can alter the carbonate equilibrium (Fig. S9). Subsequently, excess CO_2_ degassed to atmosphere, which in turn raised the pH at the later stage. In our study, the pH values remained stabilized during the second year of long-term microbial degradation of *U. prolifera*, indicating the stabilization of the carbonate system.

Indeed, the ultimate fate and carbon sequestration potential of macroalgal biomass can vary greatly under the influence of various factors alone or in combination [26,30,57–59]. For example, in shallow sediment environments, light may still penetrate. Previous studies have demonstrated that degraded kelp detritus can sustain net primary production for up to 2 months across a gradient of light attenuation along shorelines (0–30 m depth) [28]. On the other hand, light can also promote the degradation of macroalgae-derived organic carbon (i.e. photodegradation) [32,60]. Our experimental results revealed that 24.7% of the initial macroalgal biomass carbon persisted as RPOC and detrital carbon (Fig. 5), with a significant fraction theoretically buried in coastal sediments. However, the actual burial efficiency of this particulate carbon is regulated by particle-sediment dynamics. On the one hand, resuspension events may reintroduce buried POC into the water column and export them outward, thereby reducing the sedimentary carbon sink [41]; on the other hand, since minerals and anoxic environments in sediments may weaken microbial degradation of macroalgae-derived organic carbon, the actual macroalgal carbon burial effect in sediments may be higher than our experimental results in an aerobic environment. Additionally, ocean currents or hydrodynamic forces also transport deposited macroalgal biomass or detritus back into the euphotic zone or into the deep ocean [61,62]. Besides microbial degradation, the local benthic herbivores will also play a significant role in determining the fate of sunken *U. prolifera* [63]. Therefore, the *in situ* carbon sequestration of sinking macroalgae represents a highly complex process, and the combined influence of multiple factors requires further study. Notably, compared with wild benthic macroalgal forests, such as giant kelp (*Macrocystis pyrifera*), which have gained significant attention in recent years for their carbon sequestration potential, *U. prolifera* sinking to shallow seafloor sediments may exhibit a stronger sedimentary carbon sink effect. This is because wild benthic kelp typically grows attached to rock substrates, resulting in minimal carbon burial in local sediments. However, sinking *U. prolifera* can be accumulated in large quantities in muddy sediments of coastal ocean.

In conclusion, accurately determining the ultimate fate and carbon sequestration potential of sunken macroalgae remains a major challenge due to the complex and dynamic conditions of the marine environment. From a microbial degradation perspective, this study sheds light on the pivotal role of sunken macroalgae in fueling various oceanic stable/recalcitrant carbon reservoirs through microbial-mediated processes, such as the microbial carbon pump and ‘microbially driven alkalinity pump’, which are key to long-term ocean carbon sequestration. However, it is crucial to balance the ‘conflict’ between the negative ecological impacts of large-scale macroalgal blooms and their carbon sequestration benefits. Although sinking macroalgae, such as *U. prolifera* from large-scale green tides or *Sargassum* from golden tides, offer significant carbon sink potential, their rapid microbial decomposition in the early stages can trigger hypoxia and acidification in coastal waters. Therefore, developing win–win strategies that mitigate these negative environmental impacts while enhancing carbon sequestration are of paramount importance. Potential promising approaches include using natural minerals, such as montmorillonite, to improve the stability of organic carbon in sinking macroalgae. By reducing microbial decomposition, the montmorillonite may help alleviate adverse environmental impacts while strengthening long-term carbon sequestration potential of sinking macroalgae [15].

## MATERIALS AND METHODS

### Sample collection and pretreatment

**Figure 6.**
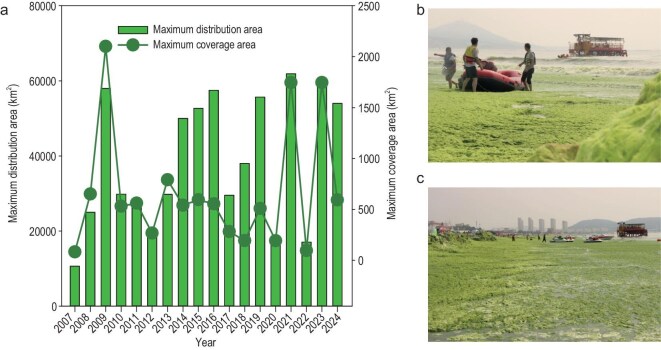
Green tides of *U. prolifera* in South Yellow Sea, China. (a) Variation of the distribution and coverage areas of green tides during 2007–24. (b and c) Photos of the massive macroalgae in nearshore area of South Yellow Sea on 23 July 2016 (i.e. the decline stage).

Fresh fronds of *U. prolifera* were sampled in the South Yellow Sea (120°51′ E; 36°4′ N) on 23 July 2016 (corresponding to the decline stage of green tide, Fig. 6). Intact thalli were selected and immediately transported to the laboratory in a 4°C cooler. On arrival at the laboratory, sterilized seawater (autoclaved at 115°C for 30 min) was used to rinse the fronds several times to eliminate epiphytes and visible particles. The seawater used for the macroalgae degradation experiments was collected from the same location as the *U. prolifera*. It was initially prefiltered through 20-μm sterile silk-bolting mesh, and subsequently through 3-μm polycarbonate membranes (Millipore Corporation, Billerica, MA, USA) to remove microalgae and larger non-living particles while preserving the natural microbial community in the filtrate. The effectiveness of microalgae removal was evaluated through integrated molecular and microscopic analyses: 16S rRNA gene sequencing to assess chloroplast 16S rRNA gene sequences and fluorescence microscopy to visually confirm the absence of intact microalgal cells in the filtered sample. After filtration, the microbial community in the filtrate was immediately processed under sterile conditions for subsequent experiments.

### Experimental design

Long-term microbial degradation of *U. prolifera* was studied in a controlled 2-year laboratory experiment. The experimental system comprised a 20-L acid-decontaminated high-density polyethylene tank, which was filled with 18 L of 3-μm filtered seawater that retained most *in situ* microorganisms. Approximately 18.0 (±0.2) g (*fw*) of *U. prolifera* was introduced into the tank as a treatment group. A control setup without the addition of *U. prolifera* was concurrently maintained. Three replicates were performed for each control and treatment. All the tanks were covered loosely to control contact with the atmosphere and incubated at ∼20°C in complete darkness for 720 days.

Prior to the degradation experiment, five random macroalgal fronds were oven-dried (GZX-9240MBE, China) at 60°C for 72 h, ground into a fine powder, and stored at −20°C for estimating macroalgal biomass carbon content. During the degradation experiment, water samples were collected at different time intervals (i.e. on Days 1, 2, 4, 6, 8, 10, 30, 60, 90, 120, 150, 180, 210, 240, 270, 300, 330, 360, 540 and 720) in replicates for DOC concentration, DOC composition (using FDOM as a proxy), microbial abundance and community composition. The molecular composition of DOC initial (Day 1) and final (Day 720) were analyzed using FT-ICR MS. Meanwhile, the fluorescent composition of POC (i.e. fluorescent particulate organic matter, FPOM), the composition of DIC (i.e. CO_3_^2^^−^, H_2_CO_3_, HCO_3_^−^ and CO_2_), and the composition of the major organic carbon in macroalgal detritus were measured on Days 1 and 720. Note that the concentrations of DIC components were calculated from DIC and pH using CO2SYS.XLS (version 24) [21,64]. At the end of the degradation experiment (Day 720), samples for measuring POC, DIC and residual algal detritus (located at the bottom of the tanks) were collected to determine the proportions of different forms of carbon. In addition, microbial biomass samples for metagenomic analysis were collected on Days 1, 5 and 720. Days 1, 5 and 720 represent the starting point of degradation, the rapid DOC release point and the end point of degradation, respectively. In this study, we categorized the macroalgae-derived DOC into two primary types, bio-labile DOC (LDOC) and bio-recalcitrant DOC (RDOC). LDOC refers to the fraction of DOC that is easily utilized by microorganisms during the degradation process, while RDOC represents the residual DOC that remains resistant to microbial degradation following the degradation phase.

Given that the microbial community remaining after long-term degradation will differ significantly from that in natural seawater, a 60-day follow-up degradation experiment was conducted to assess whether the stabilization of residual DOC on Day 720 was due to its inherent inertness. In this follow-up, the final residual macroalgal DOC was exposed to microbial communities introduced from fresh coastal seawater, along with the addition of certain inorganic nutrients. In brief, a 2.0-L aliquot from the treatment tanks was removed on Day 720. The macroalgal residual DOC was isolated by filtering through GF/F filters with a pore size of 0.7 µm. A 10-L sample of fresh seawater was obtained and prefiltered using polycarbonate membranes with a pore size of 3 μm. Subsequently, the filtrate was concentrated to 25 mL via tangential flow filtration using cartridges with a pore size of 0.2 μm, thereby capturing the natural microbial communities. A 1.0-L macroalgal residual DOC sample was inoculated with the 25 mL of concentrated natural microbial communities and inorganic nutrients (NaNO_3_ and KH_2_PO_4_: 48.5 μM and 3 μM). A control was established similarly but without the addition of natural microbial communities. The follow-up degradation experiment was conducted for 60 days in the conditions described above for the main degradation experiment. Three replicate incubations were performed for each control and treatment. Samples for measuring DOC concentrations were collected and preserved on Days 1, 2, 3, 4, 8, 16, 24, 40 and 60.

### Quantification and compositional analysis of different forms of carbon in samples

A TOC-L analyzer with an ASI-V auto-sampler (Shimadzu, TOC-L CPH, Japan) was used to measure DOC concentrations [32]. The main fluorescent compositions of dissolved organic matter and particulate organic matter (i.e. FDOM and FPOM) were examined by the 3D fluorescence spectroscopy technique excitation–emission matrices (EEM) coupled with parallel factor (PARAFAC) analysis [65]. Validation for the effect of the frozen storage conditions (−20°C) on fluorescence spectra of FDOM samples has been included in the Supplementary data (Text S2, Sample Analysis). The POC, initial macroalgal biomass carbon and residual detritus carbon were measured using a Series II CHNS/O Analyzer (PE2400, USA) [23]. DIC concentrations were measured by an infrared CO_2_ detector-based DIC analyzer (AS-C3, Apollo SciTech Inc., USA) [21]. The major organic carbon compositions contained in initial macroalgal thalli and residual macroalgal detritus were identified by Fourier transform infrared (FTIR) spectroscopy (Nicolet 6700, Thermo Fisher, USA) [66]. The molecular composition of DOC was analyzed using a 9.4 T Apex-ultra X FT-ICR MS (Bruker, Germany) under the negative mode with an Apollo II electrospray ionization (ESI) source [67,68]. Instrument settings, data calibration, acquisition, processing and formula assignment followed established protocols [68]. The elemental compositions for formula assignment were constrained to: ^12^C_0–100_, ^1^H_0–200_,^14^N_0–3_, ^16^O_0–30_ and ^32^S_0–2_. The DBE, AI* and MLB were calculated for all assigned DOC molecular formulae following FT-ICR MS analysis. All samples were measured within 2 months. Further details on the carbon sample analysis methods are provided in the Supplementary data (Supplementary Methods, Sample Analysis).

### Microbial community structure and metagenome-based function analysis

The abundance of microbial populations was assessed using a FACSAria-II flow cytometer (BD Biosciences) [69]. The composition of the microbial community was elucidated by amplifying the V4–V5 regions of the 16S rRNA genes, followed by sequencing on the Illumina MiSeq platform. The functional dynamics of the microbial community were predicted through metagenomics analysis and subsequently evaluated using WGCNA [70]. More details of the microbial sample processing, DNA extraction, sequencing and downstream analysis are described in the Supplementary data (Supplementary Methods, Sample Analysis).

### Calculation of the final proportions of different forms of carbon among macroalgal biomass carbon

At the end of the long-term microbial degradation experiment, the different forms of stable carbon remaining in the tanks (i.e. DOC, POC, DIC and residual detritus carbon) were considered as the final fate of the macroalgal biomass carbon. The percentage of each form of residual stable carbon relative to the macroalgal biomass carbon was calculated as follows:


(1) \begin{eqnarray*}
{\mathrm{Percentage\ }}C &=& \left( {C_a^{720} - C_s^{720}} \right)/\nonumber\\
&&{\mathrm{Macroalgal\ }}{{\mathrm{C}}}_{{initial}} \times 100{\mathrm{\% }}
\end{eqnarray*}

where macroalgal *C*_*initial*_ represents the initial carbon content in *U. prolifera* thalli (i.e. macroalgal biomass carbon) prior to the long-term degradation; and *C^720^_a_* and *C^720^_s_* represent the final carbon contents of different forms on Day 720 in the treatment and control groups, respectively.

## Supplementary Material

nwaf273_Supplemental_Files

## Data Availability

The 16S rRNA and metagenomic sequencing data in this study have been deposited in the China National Center for Bioinformation (CNCB) GSA database under accession code CRA005003 and CRA005004.
