# Supplementary material for: Fate and carbon sequestration potential of sunken macroalgae in coastal oceans from long-term microbial degradation perspective
Source: Natl Sci Rev. 2025 Jul 8;12(8):nwaf273. doi: 10.1093/nsr/nwaf273 (PMC12365756; doi:10.1093/nsr/nwaf273)
Supplement: nwaf273_Supplemental_Files [file nwaf273_supplemental_files.zip › Supplementary_Information.docx]

**Supplementary Information**

**Fate and carbon sequestration potential of sunken macroalgae in coastal oceans：From long-term microbial degradation perspective**

Hongmei Li ^1 †^, Zenghu Zhang ^1†*^, Jing Chen ^4 †^, Shailesh Nair ^1^, Tianqi Xiong ^5^, Hanshuang Zhao ^1^, Ding He ^6^, Kitack Lee ^7^, Nianzhi Jiao ^8*^, Yongyu Zhang ^1, 2, 3*^

^1^ Qingdao New Energy Shandong Laboratory, Qingdao Institute of Bioenergy and Bioprocess Technology, Chinese Academy of Sciences, Qingdao 266101, China

^2^ Southern Marine Science and Engineering Guangdong Laboratory (Zhuhai), Zhuhai 519782,China

^3^ Laboratory for Marine Biology and Biotechnology, Qingdao Marine Science and Technology Center, Qingdao, 266237, China

^4^ College of Chemistry and Environment, Ankang University, Ankang 725000, China

^5^ College of Environmental Science and Engineering, Qingdao University, Qingdao, 266071, China

^6^ Department of Ocean Science and Center for Ocean Research in Hong Kong and Macau, The Hong Kong University of Science and Technology, Hong Kong, China

^7^ Division of Environmental Science and Engineering, Pohang University of Science and Technology, Pohang 37673, Korea

^8^ Carbon Neutral Innovation Research Center and Fujian Key Laboratory of Marine Carbon Sequestration, Xiamen University, Xiamen, 361101, China

***Correspondence**:

Yongyu Zhang, [zhangyy@qibebt.ac.cn](mailto:zhangyy@qibebt.ac.cn); Zenghu Zhang, [zhang_zh@qibebt.ac.cn](mailto:zhang_zh@qibebt.ac.cn); Nianzhi Jiao, [Jiao@xmu.edu.cn](mailto:Jiao@xmu.edu.cn)

† Hongmei Li, Zenghu Zhang and Jing Chen contributed equally to this work.

**Supplementary Information Guide**

Supplementary Figure S1 to S10

Supplementary Table S1 to S5

Supplementary Methods

Supplementary References


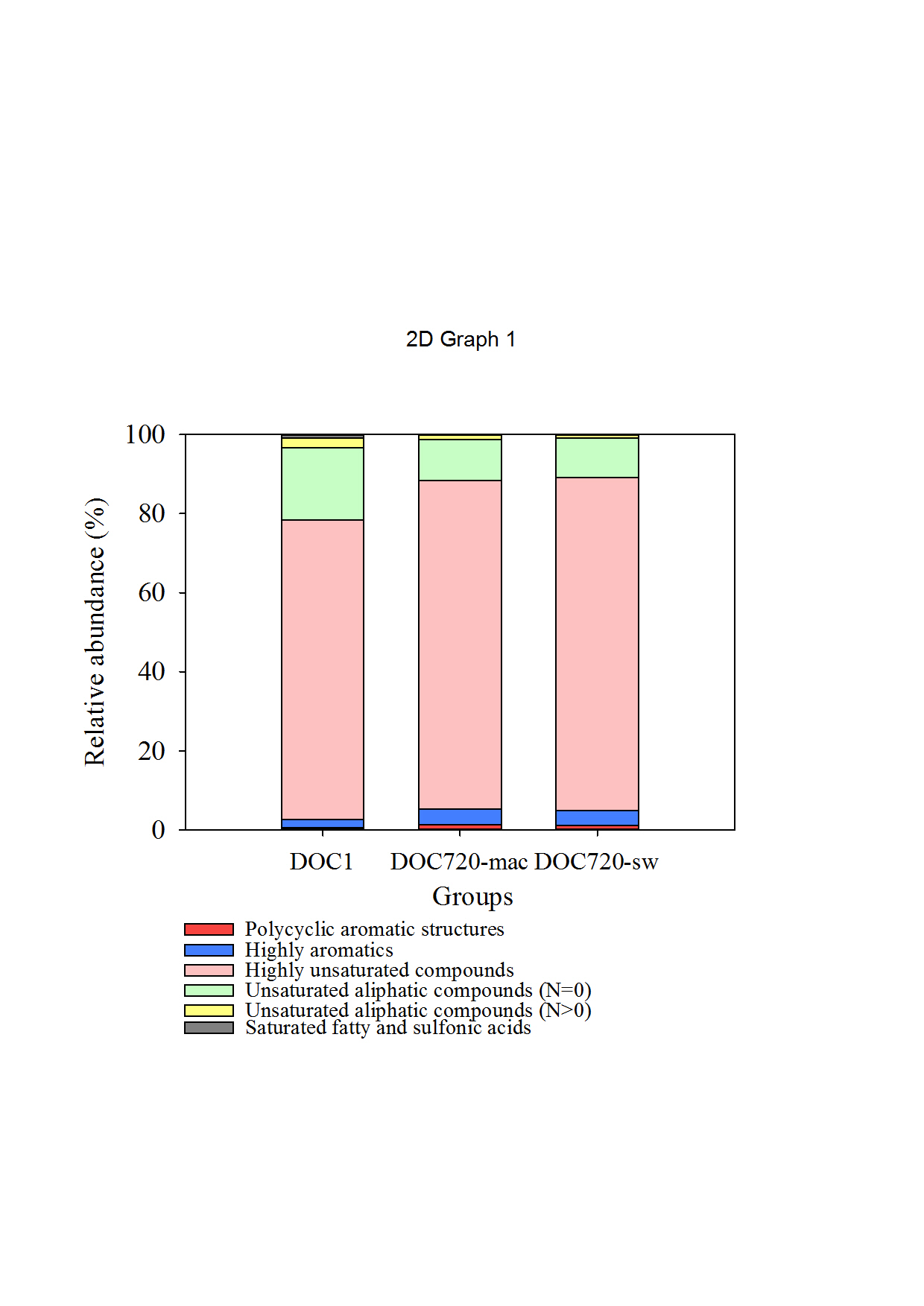


**Supplementary Figure S1:** Molecular classification (peak-intensity-weighted percentage) of DOC identified with the aromaticity index (*AI**) value and H/C ratio among the DOC in the treatment (DOC-mac) and control (DOC-sw) groups on days 1 and 720 of the long-term degradation. According to the *AI** value and H/C ratio, all the DOC molecules can be classified into different types of substances [1], *AI** >0.66 is an unambiguous criterion for characterising polycyclic aromatic molecules; and the molecules with an 0.5<AI≤0.66 are highly aromatic substances; the molecules with AI≤0.5 and H/C<1.5 are usually highly unsaturated compounds; the molecules with AI≤0.5, 1.5≤H/C<2 and N=0 (Nitrogen atom) represent unsaturated aliphatic compounds without nitrogen; AI≤0.5, 1.5≤H/C<2 and N>0 are indicative of nitrogenous unsaturated aliphatic compounds; and AI≤0.5, H/C≥2 are indicative of saturated fatty and sulfonic acids.


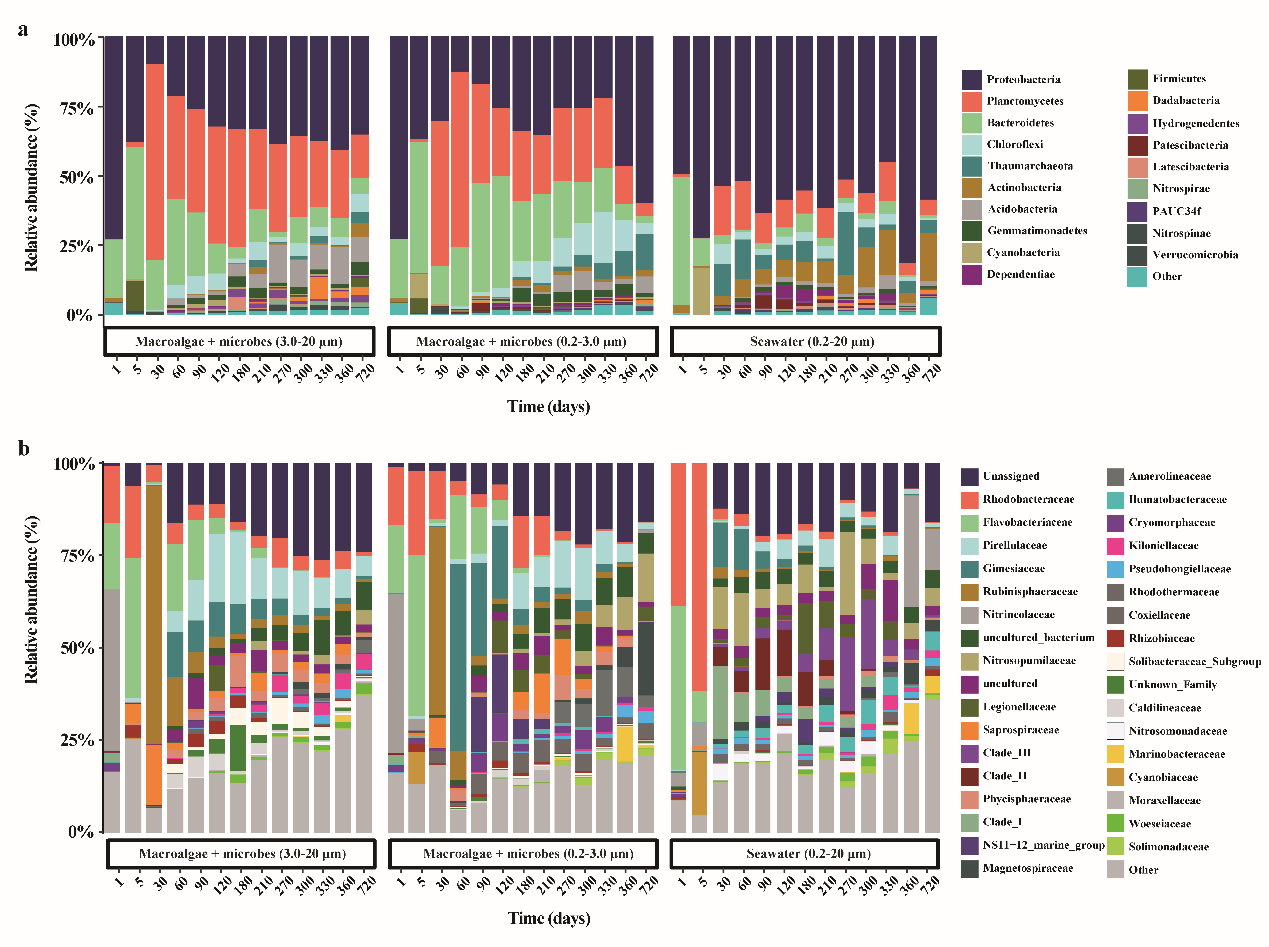


**Supplementary Figure S2:** Dynamic changes in microbial community structure at the phylum (a) and family (b) levels in the treatment and control groups during the long-term degradation process.


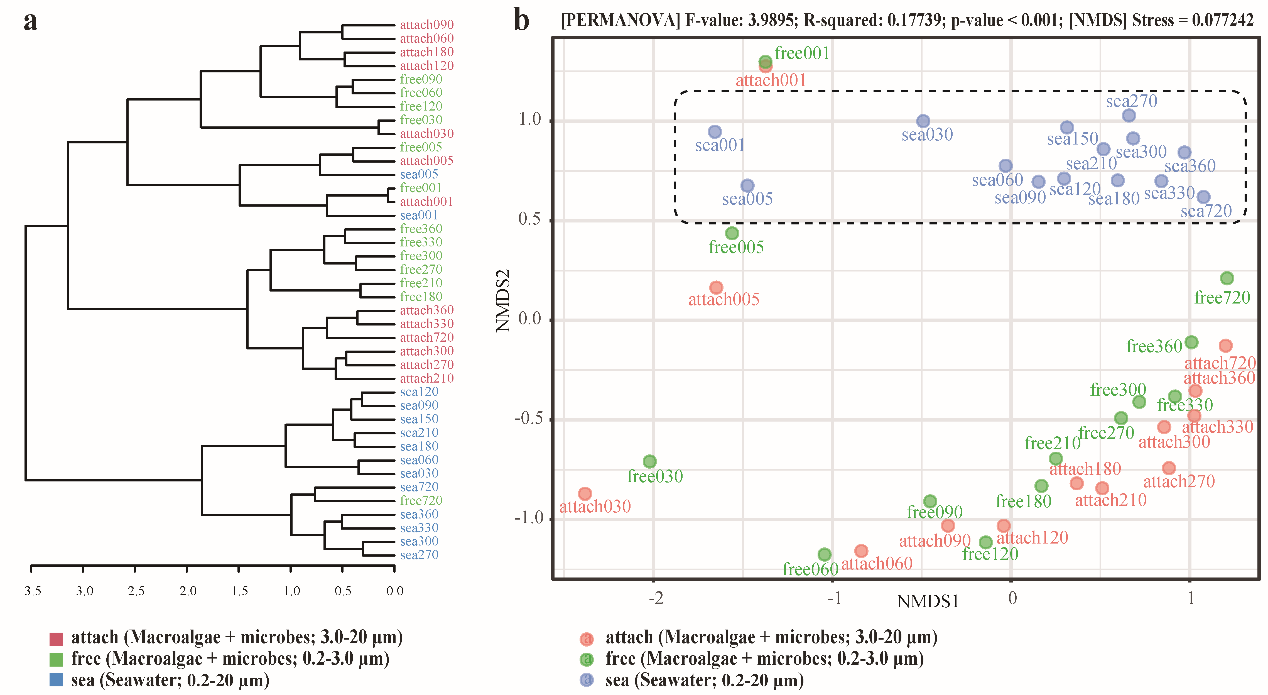


**Supplementary Figure S3:** Hierarchical cluster analysis (a) and nonmetric multidimensional scaling ordination (b) of the microbial communities sampled at different times during the long-term degradation process.


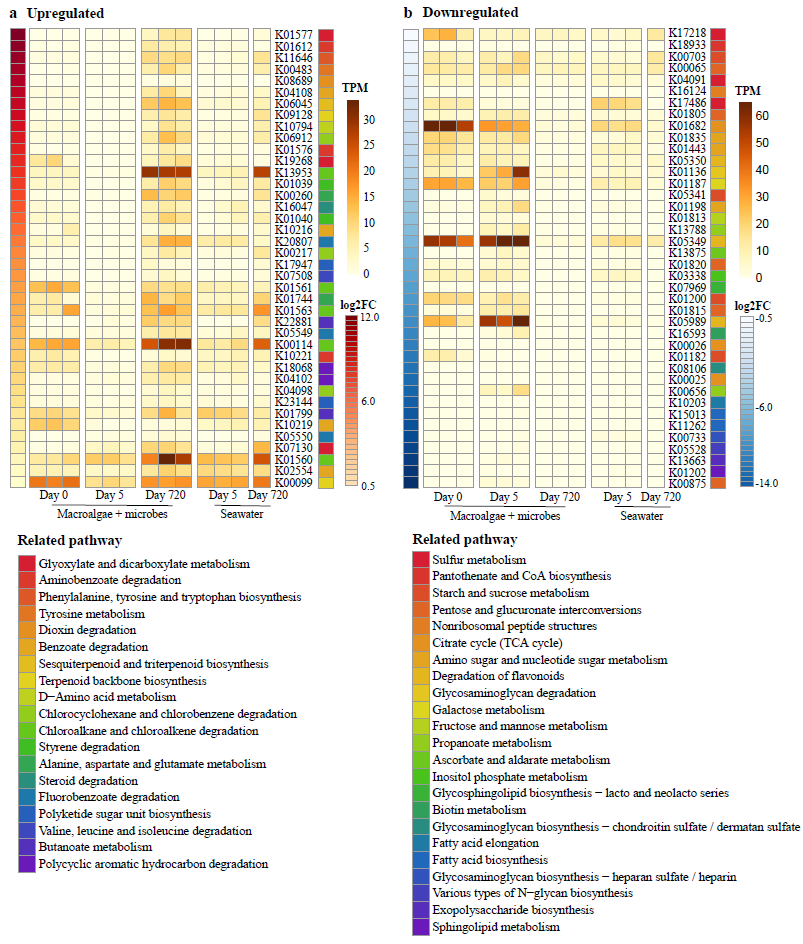


**Supplementary Figure S4:** Abundance of differentially expressed bacterial genes during the long-term degradation. Heatmaps showing (A) upregulated and (B) downregulated bacterial genes (KEGG orthologs) and their associated pathways in degradation over time. Color intensity indicates gene abundance in transcripts per million (TPM).

*
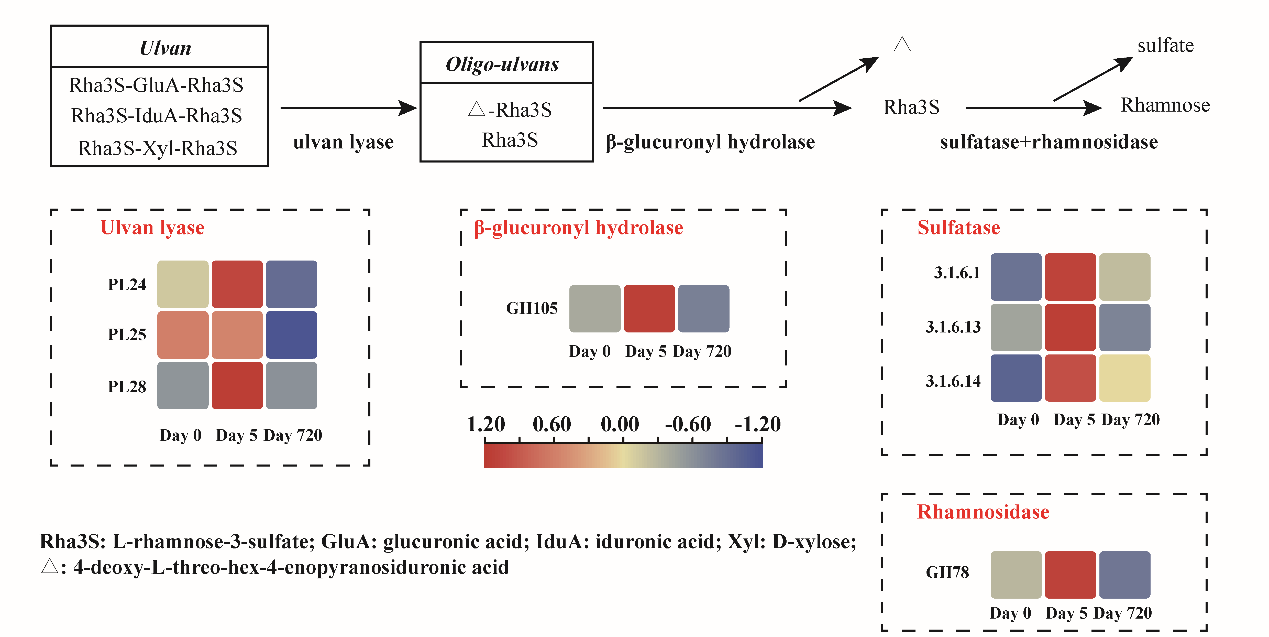
*

**Supplementary Figure S5:** Sulfated polysaccharide ulvan degradation dynamics predicted by microbial functional gene profiling.


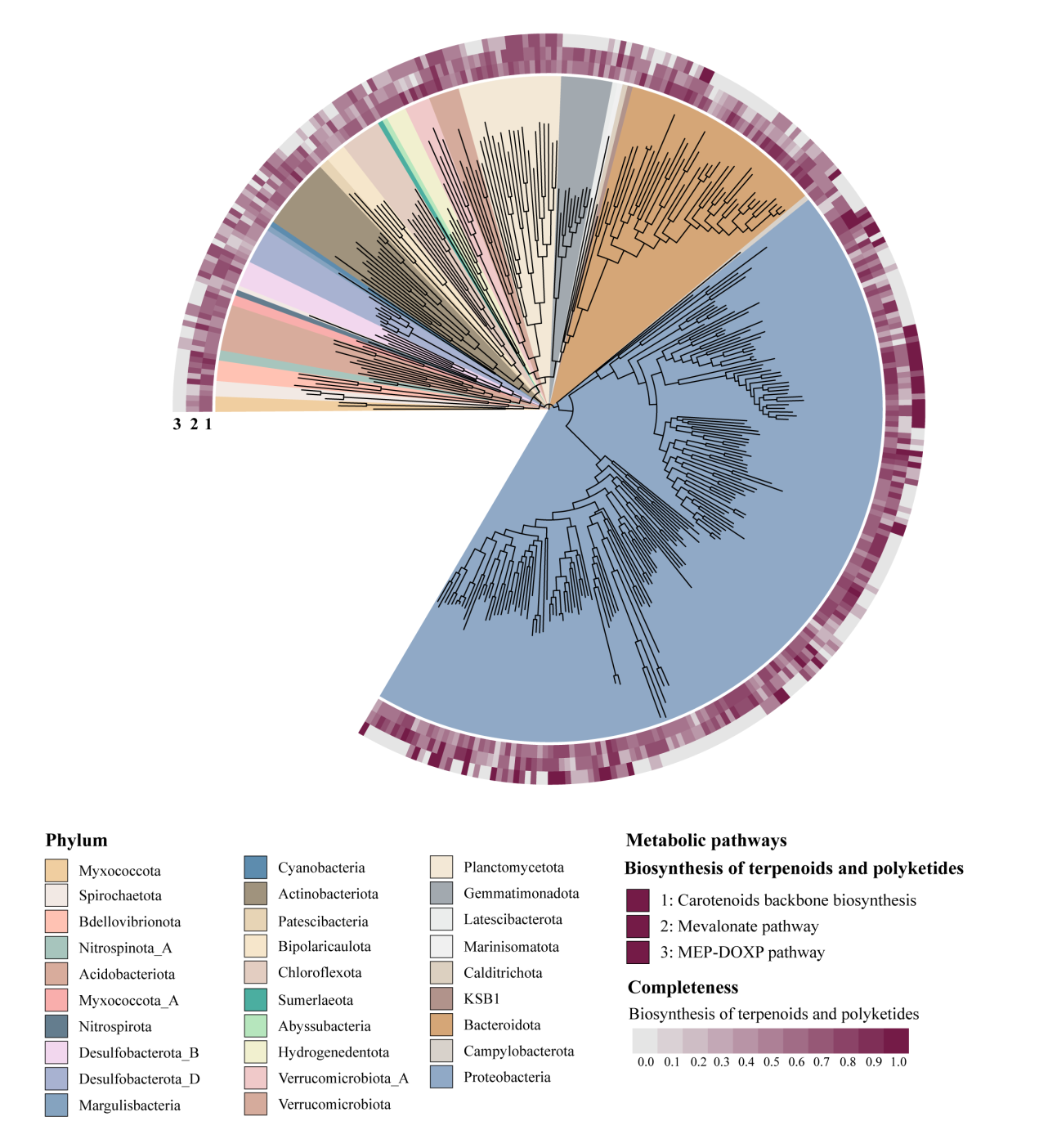


**Supplementary Figure S6:** Genomic and functional annotation of 338 bacterial metagenome‑assembled genomes (MAGs). The phylogenetic tree was constructed using IQ-TREE by 120 concatenate markers gene. The color of a phylogenetic tree according to phylum classification. Heatmap in the outer circle represents the metabolic pathway completeness of the MAGs based on the presence or absence of genes as determined by KEGG Decoder.


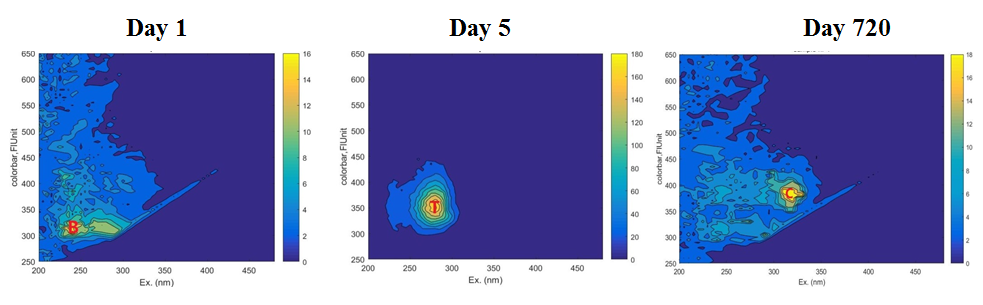


**Supplementary Figure S7:** Fluorescent components of POC (i.e., FPOM) in the treatment group on days 1, 5 and 720 of the long-term degradation. Among them, peaks B, T and C represent protein-like fluorophore (tyrosine-like), protein-like fluorophore (tryptophan-like), and humic-like fluorophore, respectively. In the three panels, high fluorescence intensity of protein-like fraction emerged at the early degradation stage (day 5), whereas high fluorescence intensity of humic-like fraction presented at the end of degradation (day 720).

**
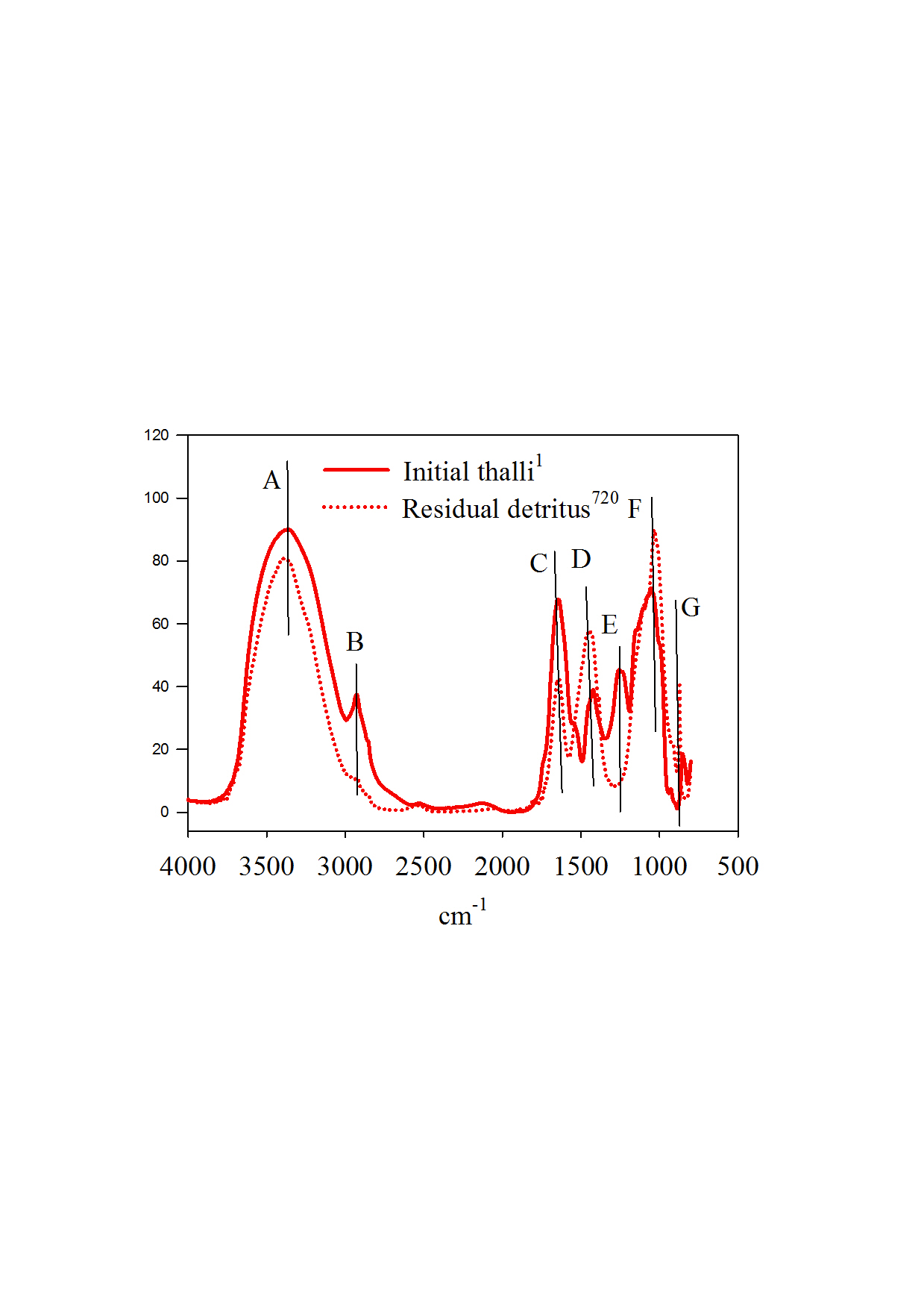
**

**Supplementary Figure S8:** FTIR spectra of initial *U. prolifera* thalli on day 1 and residual algal detritus on day 720 of the long-term degradation. Generally, there were seven characteristic infrared peaks being recorded, among which peaks A, B, C, D, E, F and G were identified associated with polysaccharides, lipids, proteins, phenol, polysaccharides, humic substances and condensed aromatic compounds, respectively via their infrared spectra (Table S5). In comparison with day 1, the peaks A, B, C and E showed low absorbance on day 720, while the peaks D, F and G presented obviously high absorbance after the long-term degradation.


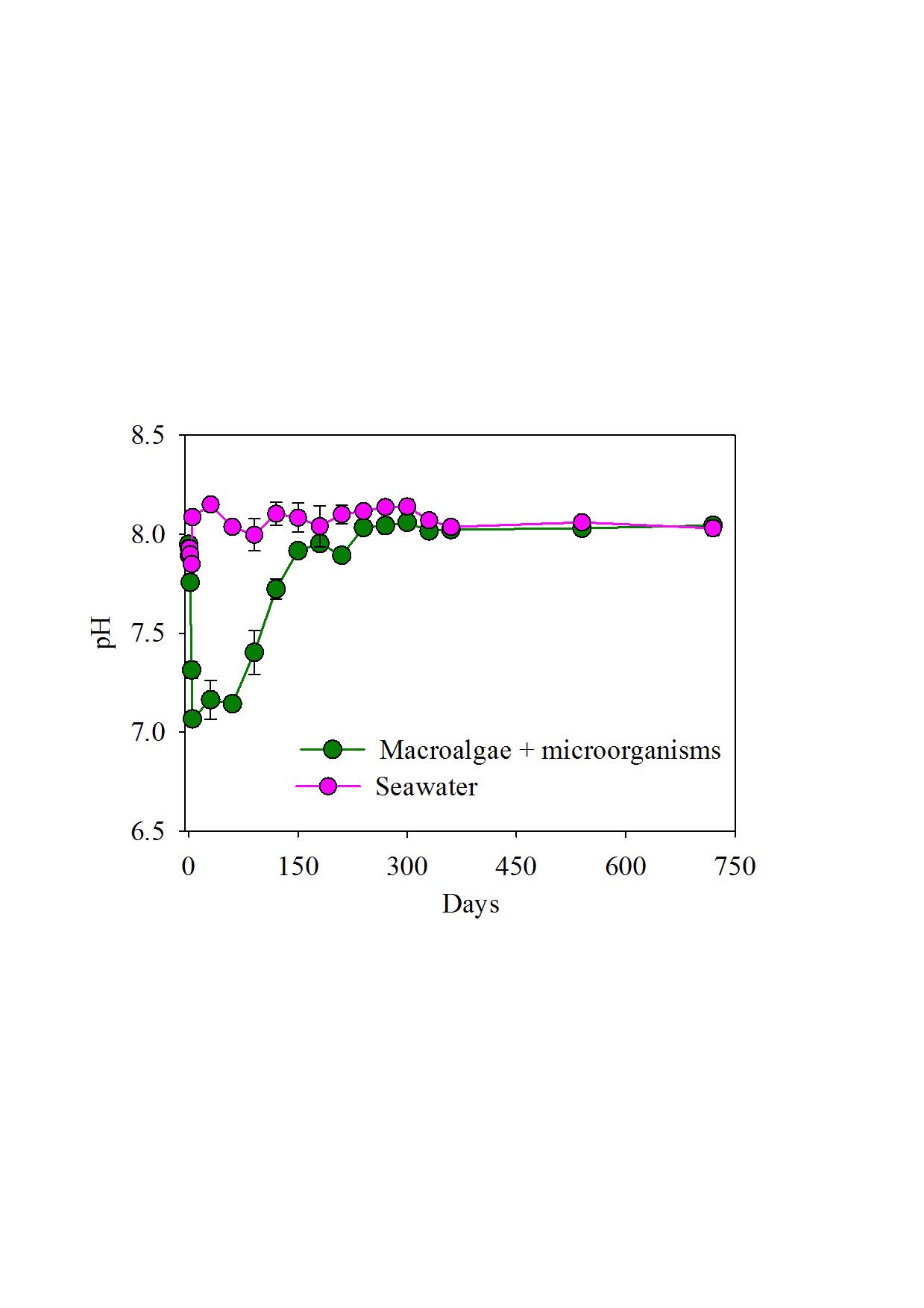


**Supplementary Figure S9:** Dynamic changes in pH in the treatment (macroalgae + microorganisms) and control (seawater) groups during the long-term degradation process.

**Supplementary Tables:**

**Supplementary Table S1:** Main components (C1, C2 and C3) of FDOM identified by EEM-PARAFAC analysis during the long-term degradation process, and the comparison of these components with previously identified components.

| Components | Ex/Em | Description | References |
| --- | --- | --- | --- |
| Component 1 | 225 (275)/340 | protein-like substance | C5:270/332 [2]  C6:280/338 [3] |
| Component 2 | 250 (350)/420 | humic-like substance | C8:250/416 [4]  C2:250/420 [2] |
| Component 3 | 275(370)/490 | humic-like substance | C3:260(370)/490 [4]  C3:(260)395/515 [5] |

**Supplementary Table S2:** KEGG pathways in each sample on days 0, 5 and 720 of the long-term degradation.

The data were available in an Excel format (see Supplementary file Table S2).

**Supplementary Table S3:** Different modules generated by WGCNA and their corresponding functional profiles.

The data were available in an Excel format (see Supplementary file Table S3).

**Supplementary Table S4:** Computational details of the final proportions of different forms of carbon among the total macroalgal biomass carbon.

| Carbon forms | Final contents  (means ± SD, μmol) | macroalgal biomass carbon^1^  (means ± SD, μmol) | Final proportions  (%) |
| --- | --- | --- | --- |
| DOC_f_^2^ | 63.2±1.9 | 1920.0±26.0 | 3.3 |
| POC_f_^2^ | 426.7±3.2 | 1920.0±26.0 | 22.2 |
| DIC_f_^2^ | 195.7±9.4 | 1920.0±26.0 | 10.2 |
| Detritus C_f_^3^ | 48.9±0.5 | 1920.0±26.0 | 2.5 |
| Carbon gases^4^ | -- | 1920.0±26.0 | 61.8 |

^1^Macroalgal biomass carbon represents the initial carbon content in *U. prolifera* thalli prior to the long-term degradation. ^2^Final concentrations of different forms of carbon were according to the following three equations: DOC_f_ = DOC^720^_a_ - DOC^720^_s_; POC_f_ = POC^720^_a_ - POC^720^_s_; DIC_f_ = DIC^720^_a_ - DIC^720^_s_; Among them, the DOC_f_, POC_f_ and DIC_f_ represent the final contents of different forms of carbon after the long-term degradation; the DOC^720^_a_, POC^720^_a_ and DIC^720^_a_ represent the contents of different forms of carbon on day 720 in the treatment group; the DOC^720^_s_, POC^720^_s_ and DIC^720^_s_ represent the contents of different forms of carbon on day 720 in the control group. ^3^Detritus C_f_ represents the carbon content of residual algal detritus on day 720 in each tank. ^4^The final proportion of carbon gas was a 100% minus the final proportions of DOC, POC, DIC, and residual algal detritus carbon.

**Supplementary Table S5:** Absorption bands identified in the infrared spectra of initial *U. prolifera* thalli on day 1 and residual algal detritus on day 720 of the long-term degradation^1^.

| Peak^2^ | Wavelength (cm^-1^) | Functional group | Compounds |
| --- | --- | --- | --- |
| A | 3400-3360 ^(i)^ | Stretching OH | Polysaccharides |
| B | 2930 ^(w)^ | Stretching -CH_2_ | Lipids |
| C | 1650-1630^(i)^ | Stretching C-O & C-N (Amide I) | Proteins |
| D | 1440-1420 ^(w)^ | Phenolic -OH; asymmetric vibrations of C–C aromatic ring | Phenol compounds |
| E | 1260^(w)^ | Stretching C-O & OH deformation of COOH | Polysaccharides |
| F | 1070-1060 ^(i)^ | CH aromatic | Humic substances |
| G | 880-850 ^(w)^ | C–H o-substituted aromatic ring | Condensed aromatic compounds |

^1^Interpretation of infrared spectra was based on Davis et al. [6], Naidja et al. [7], Bellgrove et al. [8], Mishra and Jha [9], Nwodo et al. [10], and Villacorte et al. [11].

^2^Peak codes as indicated in Fig. S6; (i) -intense band; (w) -weak band.

**Supplementary Methods**

**Text S1. Sample Collection**

During the long-term degradation process, water samples of DOC, FDOM and FT-ICR MS analysis were filtered through pre-combusted (at 450°C for 5 h) 0.7-μm GF/F filters and stored in 40-mL (for DOC concentration and FDOM composition) and 500-mL (for FT-ICR MS) brown glass bottles at -20°C. After the degradation, to measure POC, a volume of 100-mL seawater was collected under low vacuum on pre-combusted 0.7-μm GF/F filters and then frozen at -20°C. To measure DIC, 60-mL unfiltered seawater with 50-μL saturated mercury chloride solution was kept at room temperature. To measure residual algal detrital carbon, the algal detritus was obtained by centrifuging the incubation medium and discarding the supernatant, after which the detrital carbon was determined for the remaining pellet in the same way as it was for macroalgal biomass carbon prior to degradation. To analyze the microbial abundance, 1.8-mL unfiltered seawater was fixed with glutaraldehyde (final concentration: 0.5%) directly, flash-frozen in liquid nitrogen and stored at -80°C. To analyze microbial community composition, 300-mL water samples were pre-filtered using 20-μm sieve to remove larger particles, and then passed sequentially through 3.0 and 0.2-μm pore-size polycarbonate filters. The filters were then stored at -80°C. The extra 1.0-L samples on days 1, 5, and 720 were filtered according to the above steps for the analysis of the microbial community function.

**Text S2. Sample Analysis**

**Concentrations of DOC:** To determine the DOC concentrations, a Shimadzu TOC-L analyzer connected with an ASI-V auto-sampler was employed for analysis [12]. Seawater DOC standards, including the low carbon water and deep seawater, produced at Hansell’s laboratory at the University of Miami, were analyzed to maintain data quality control [13]. Moreover, the blank was deducted using Milli-Q water analysis before every five samples. The average blanks associated with the DOC measurement were ~5 μM, and the analytic precision on the triplicate injections was ± 3% [14].

**Main components of FPOM and FDOM:** Three-dimensional (3D) fluorescence spectroscopy technique coupled with parallel factor analysis was applied to analyze the main components of FPOM and FDOM. The FDOM samples were thawed to 20°C and filtered through 0.2-μm pore polyether sulfone membrane filters prior to fluorescence scans. As for FPOM samples, fluorescent material was extracted into 10 mL of 0.1 N NaOH for 24 h in the dark at 4°C, and then were neutralized with HCl to a pH near that of the original sample [15, 16], after which the resultant solution was filtered through 0.2-μm polyether sulfone filters prior to measurement. A Hitachi F-4600 spectro-fluorometer (Hitachi, Japan) was employed to measure excitation–emission matrixes (EEMs), with parameters from 200 nm to 480 nm excitation (5 nm intervals) and from 250 nm to 650 nm emission (5 nm intervals) at a scanning speed of 1200 nm min^-1^ and an integration time of 0.05 s [14]. FDOM and FPOM samples were analyzed within two months of collection. To verify the fluorescence spectra of FDOM samples under frozen storage (−20°C), we scanned the fluorescence spectra of three samples under refrigerated and frozen conditions. The results showed that there was no noticeable difference in the fluorescence spectra of the same sample under different storage conditions (Fig. S10).

The Raman peak and Rayleigh scattering were removed from the EEMs, and the missing regions were filled by three-dimensional Delaunay interpolation of the surrounding data points [17]. Fluorescence intensities in photons s^-1^ were converted to quinine sulfate units (QSU; 1 QSU = 1 ppb quinine sulfate in 0.05 M H_2_SO_4_) with a calibration curve [18]. Data were analysed using MATLAB (Mathworks, Natick, MA) and the dissolved organic matter Fluor toolbox (<http://www.models.life.ku.dk/>) [19].


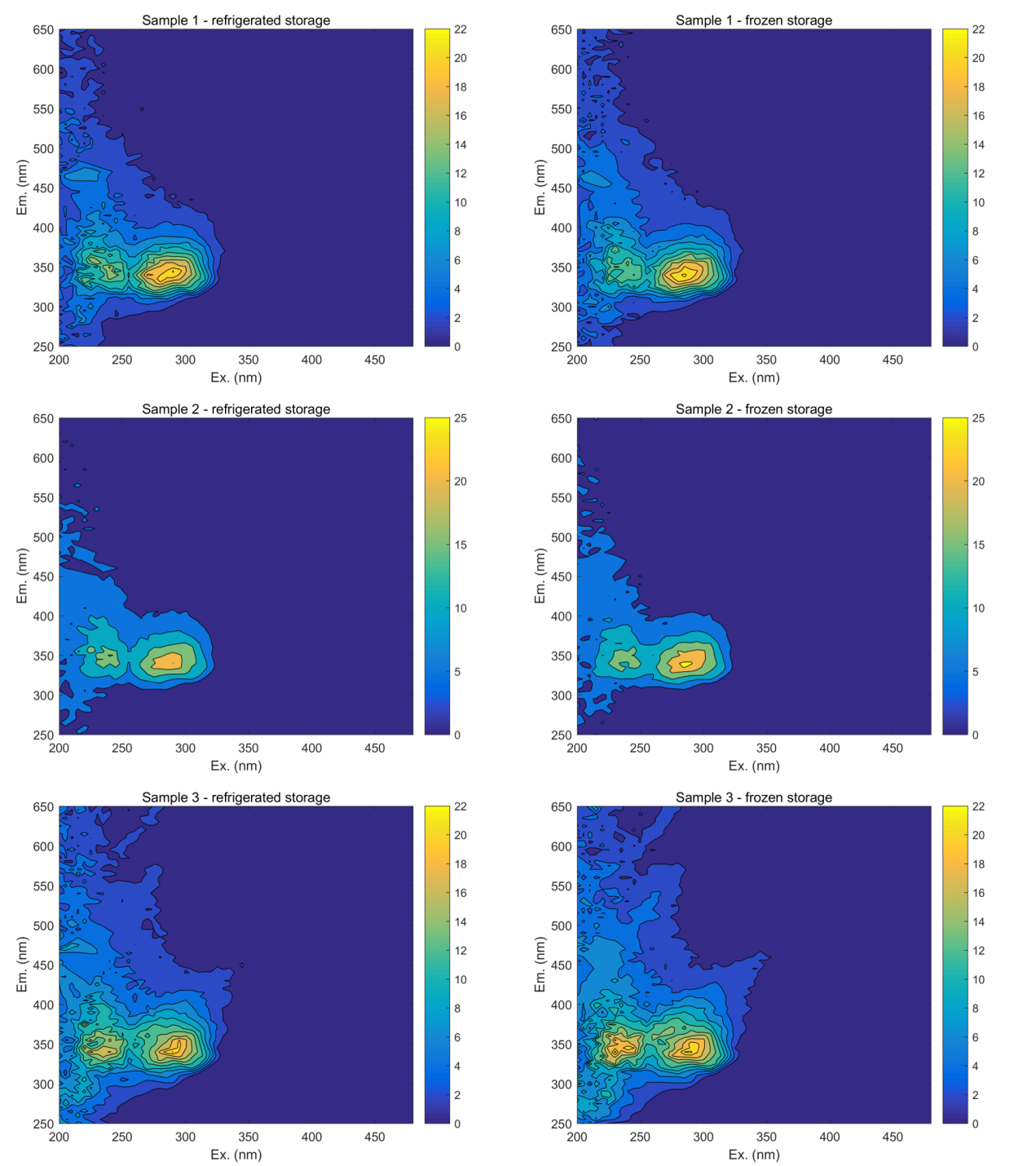


**Supplementary Figure S10:** Fluorescence spectra of three FDOM samples under refrigerated (4°C, within 24 hours) and frozen (−20°C, within two months) storage, respectively.

**Molecular composition of DOC:** Before the FT-ICR MS analysis, three parallel samples that collected during the same time were combined as Chen et al. [20], and DOM were extracted by the solid phase extraction (SPE) method (PPL cartridge, Agilent Bond Elut; 500 mg) [21]. Briefly, SPE cartridges were activated by rinsing with 18 mL methanol and washing with 18 mL acidified ultrapure water (pH = 2) prior to extraction. The samples were slightly modified by the addition of 0.1% formic acid to prevent the potential formation of chloride adducts in the solid phase extracted DOC. The acidified samples passed through the SPE cartridge at a rate of 5 mL min^-1^. The cartridge was then washed with 18 mL acidified ultrapure water (pH = 2), dried at room temperature with N_2_, and eluted with 10-mL pure methanol.

The molecular composition of DOC were analyzed using a Bruker Apex-ultra FT-ICR MS spectrometry equipped with a 9.4 T superconducting magnet and Apollo II electrospray (ESI) ion source. Methanol PPL extracts were diluted to a DOC concentration of ~50 mg L^-1^ and injected into the ESI source at a rate of 250 mL h^-1^. The typical operating conditions for negative-ion ESI analysis were: spray shield voltage of 3.5 kV, capillary column introduced voltage of 4.0 kV, and capillary column end voltage of -300 V; ions were accumulated in the collision cell for 0.2 s then were transferred into the ICR cell with a time-of-flight (ToF) of 1.1 ms. The ion transformation parameter for the quadrupole (Q1) was optimised at m/z 300. The mass range was m/z 200–800. A total of 128 scans with 2 M word size were accumulated to enhance the signal-to-noise ratio.

The methodologies that described as He et al. were selected for FT-ICR MS calibration [22], data acquisition, and processing. In brief, the FT-ICR MS was calibrated using a known homologous series of the SRFA sample (Suwannee River Fulvic acids, obtained from IHSS, USA), which contains a relatively high abundance of oxygen-containing compounds. Mass peaks with a signal-to-noise (S/N) ratio > 4, were exported to a data sheet. Data analysis was performed using in-house software. The assigned formulae were limited in the following elemental composition: ^12^C_0–60_, ^1^H_0–120_,^14^N_0–3_,^16^O_0–30_ and ^32^S_0–2_. The mass accuracy window was set to 1.0 ppm in the formula assignment section. Three basic chemical criteria was set for all elemental formulae: (1) the number of H atoms should be at least 1/3 that of C atoms and cannot be greater than that of 2C + N + 2; (2) the sum number of N and H atoms should be even; and (3) the H/C and O/C value should be restricted to be less than 3 and 1.5, respectively.

**Concentrations of macroalgal biomass carbon, algal detrital carbon, POC, DIC and DIC components:** The POC concentration, macroalgal biomass carbon and algal detrital carbon were measured by Series II CHNS/O Analyzer (PE2400, USA), among them the POC samples were fumed with HCl to remove carbonates in prior to determination, whereas measurement of macroalgal biomass carbon and algal detrital carbon was not fumed with acid. Acetanilide was employed as a standard, and the relative standard deviation of analysis was < 2%. The DIC concentrations were determined using an infrared CO_2_ detector-based DIC analyzer (AS-C3; Apollo SciTech Inc., USA), which were calibrated against Certificated Reference Materials (CRM) from Andrew G. Dickson’s lab at Scripps Institution of Oceanography at an uncertainty level of ±2 μmol kg^−1^. The concentrations of DIC components (CO_3_^2-^, HCO_3_^-^, H_2_CO_3_ and CO_2_) were calculated from DIC and pH using CO2SYS.XLS (version 24) [23].

**Main organic compositions in algal detritus:** The major organic components (based on the functional groups) contained in initial algal thalli and residual algal detritus after the degradation were identified by Fourier transform infrared (FTIR) spectroscopy (Nicolet 6700, Thermo Fisher, USA) [11]. An infrared (IR) spectrum was generated after passing a beam of IR light through the algal detritus. Absorption was recorded when the frequency of the IR light coincided with the vibrational frequency of the covalent bonds in some of molecules present in the detrital samples.

**Microbial abundance:** The microbial abundance was determined using the cytometer Facs-II Aria (BD Biosciences) according to Liang et al. [24]. Before analysis the samples were unfrozen at 37°C and stained with the SYBR Green I in the dark for 15 min at 20°C, then were determined at a flow rate of 0.1-1 mL h^−1^.

**Microbial community structure:** Three parallel samples at each sampling time point were combined before DNA isolation according to Zhao et al. [25]. Total genomic DNA was extracted from samples collected at different time points using the CTAB (cetyltrimethyl ammonium bromide) method [26]. The high-quality DNA samples were sequenced on the Illumina MiSeq platform (Majorbio Co. Ltd., Shanghai, China) using universal bacterial primers 515F (5’-GTGCCAGCMGCCGCGGTAA-3’) and 907R (5’-CCGTCAATTCMTTTRAGTTT-3’) for targeting the hypervariable V4-V5 region of the 16S rRNA gene. The paired-end reads were merged and quality-filtered via vsearch (version 2.13.6). Processed reads were dereplicated using vsearch -derep_fulllength with the minuniquesize 8 options, and finally denoised to produce the amplicon sequence variants (ASVs) using the usearch -unoise3 command with default value [27]. Chimeras were identified and removed using vsearch -uchime_ref and –db Silva database (release 132). Representative sequences were annotated against the Silva database (release 132) [28]. Alpha diversity indexes were calculated using the R package “vegan”. The 16S rRNA gene reads were deposited in the National Genomics Data Center (NGDC) Genome Sequence Archive database (accession Number: CRA005004).

**Microbial community function**: Three time point samples (i.e., days 1, 5 and 720) were selected for metagenomics analysis. The total genomic DNA of each sample was extracted using the MagPure Soil DNA KF Kit and sent to Oebiotech Company (Shanghai, China) for sequencing on an Illumina NovaSeq6000. The raw reads were deposited in the National Genomics Data Center (NGDC) Genome Sequence Archive database (accession Number: CRA005003). After sequencing, the raw reads were quality-filtered using the KneadData pipeline (https://bitbucket.org/biobakery/kneaddata) with Trimmomatic method [29]. The sequences of *U. prolifera* (GenBank accession: GCA_004138255) were removed using bowtie2. The clean data were assembled using Megahit and contigs were generated [30]. Contigs with a length of more than 500 bp were selected for subsequent analysis. Gene prediction on the assembled contigs was performed using Prodigal software [31]. The genes were annotated using diamond software against the Kyoto Encyclopedia of Genes and Genomes (KEGG) database and Carbohydrate-Active EnZymes (CAZy) databases [32, 33]. Differential abundance of KEGG orthologs (KOs) between day 5 and day 720 of long-term macroalgal degradation was determined with DESeq2 with a false discovery rate (FDR) adjusted *p*-value (*p-adj*) of < 0.05 [34]. In addition, the assembled contigs were binned using the MetaWRAP pipeline to obtain MAGs [35]. CheckM was employed to evaluate the quality of the MAGs and those MAGs with >50% completeness with <10% contamination were kept for further analysis [36]. The taxonomic identity of the 338 bacteria MAGs was determined using the Genome taxonomy database toolkit (GTDB-Tk) (Chaumeil et al., 2020) with the GTDB database [37]. A phylogenetic tree was constructed through IQ-TREE with MFP evolutionary model and the ultrafast bootstrapping value of 1000 based on multiple sequence alignment file generated by GTDB-Tk [9]. The tree was visualized using iTOL [38]. Finally, the metabolic potential of MAGs was predicted by KEGG-Decoder (www.github.com/bjtully/BioData/tree/master/KEGGDecoder).

Highly correlated functional genes were clustered into gene modules and their connection with the DOC composition was identified using weighted gene co-expression network analysis (WGCNA) [39]. In brief, the adjacency matrix of the functional genes was calculated based on the threshold parameters which were generated using the function pickSoftThreshold. Gene clustering tree was constructed based on the dissimilarity between genes, and different modules were generated by Dynamic Branch Cut methods (minimum of 20 genes per module). The modules whose eigengenes were highly correlated (r > 0.8) were merged by the function mergeCloseModules. The statistical significance between module eigengenes and the traits was defined using Pearson correlation and p-value from the univariate regression model. Ternary plots were used to visualize the changes in the functional genes and modules of the microbial community along with the degradation process. The points near the corners of the ternary plot represent the abundant functional genes at the corresponding time points (i.e., days 1, 5 and 720). Microbial functions were classified as “specialized functions” if their relative abundance within certain time point was greater than 60% (an arbitrary threshold). Other functions were classified as “core functions” [40]. Here, 1442 core functions and 566 specialized functions were assigned in the macroalgal degradation groups. These specialized functions can reflect the corresponding environmental characteristics (e.g. DOC composition).

**References**

[1] Šantl-Temkiv T, Finster K, Dittmar T, et al. Hailstones: A window into the microbial and chemical inventory of a storm cloud. Plos One, 2013, 8:

[2] Kowalczuk P, Durako MJ, Young H, et al. Characterization of dissolved organic matter fluorescence in the south atlantic bight with use of parafac model: Interannual variability. Mar Chem, 2009, 113: 182-196

[3] Stedmon CA, Markager S. Resolving the variability in dissolved organic matter fluorescence in a temperate estuary and its catchment using parafac analysis. Limnol Oceanogr, 2005, 50: 686-697

[4] Murphy KR, Stedmon CA, Waite TD, et al. Distinguishing between terrestrial and autochthonous organic matter sources in marine environments using fluorescence spectroscopy. Mar Chem, 2008, 108: 40-58

[5] Bai Y, Su R, Yao Q, et al. Characterization of chromophoric dissolved organic matter (cdom) in the bohai sea and the yellow sea using excitation-emission matrix spectroscopy (eems) and parallel factor analysis (parafac). Estuar Coast, 2017, 40: 1325-1345

[6] Davis WM, Erickson CL, Johnston CT, et al. Quantitative fourier transform infrared spectroscopic investigation of humic substance functional group composition. Chemosphere, 1999, 38: 2913-2928

[7] Naidja A, Huang PM, Anderson DW, et al. Fourier transform infrared, uv-visible, and x-ray diffraction analyses of organic matter in humin, humic acid, and fulvic acid fractions in soil exposed to elevated co2 and n fertilization. Appl Spectrosc, 2002, 56: 318-324

[8] Bellgrove A, Kihara H, Iwata A, et al. Fourier transform infrared microspectroscopy as a tool to identify macroalgal propagules. J Phycol, 2009, 45: 560-570

[9] Mishra A, Jha B. Isolation and characterization of extracellular polymeric substances from micro-algae dunaliella salina under salt stress. Bioresource Technol, 2009, 100: 3382-3386

[10] Nwodo UU, Agunbiade MO, Green E, et al. Characterization of an exopolymeric flocculant produced by a brachybacterium sp. Materials, 2013, 6: 1237-1254

[11] Villacorte LO, Ekowati Y, Neu TR, et al. Characterisation of algal organic matter produced by bloom-forming marine and freshwater algae. Water Res, 2015, 73: 216-230

[12] Benner R, Strom M. A critical evaluation of the analytical blank associated with doc measurements by high-temperature catalytic oxidation. Mar Chem, 1993, 41: 153-160

[13] Dai M, Yin Z, Meng F, et al. Spatial distribution of riverine doc inputs to the ocean: An updated global synthesis. Curr Opin Environ Sustain, 2012, 4: 170-178

[14] Li HM, Zhang YY, Liang YT, et al. Impacts of maricultural activities on characteristics of dissolved organic carbon and nutrients in a typical raft-culture area of the yellow sea, north china. Mar Pollut Bull, 2018, 137: 456-464

[15] Osburn CL, Handsel LT, Mikan MP, et al. Fluorescence tracking of dissolved and particulate organic matter quality in a river-dominated estuary. Environ Sci Technol, 2012, 46: 8628-8636

[16] Santin C, Yamashita Y, Otero XL, et al. Characterizing humic substances from estuarine soils and sediments by excitation-emission matrix spectroscopy and parallel factor analysis. Biogeochemistry, 2009, 96: 131-147

[17] Barber CB, Dobkin DP, Huhdanpaa H. The quickhull algorithm for convex hulls. ACM Trans Math Softw, 1996, 22: 469-483

[18] Mopper K, Schultz CA. Fluorescence as a possible tool for studying the nature and water column distribution of doc components. Mar Chem, 1993, 41: 229-238

[19] Coble PG. Characterization of marine and terrestrial dom in seawater using excitation emission matrix spectroscopy. Mar Chem, 1996, 51: 325-346

[20] Chen J, Li HM, Zhang ZH, et al. Doc dynamics and bacterial community succession during long-term degradation of ulva prolifera and their implications for the legacy effect of green tides on refractory doc pool in seawater. Water Res, 2020, 185: 116268

[21] Gonsior M, Schmitt-Kopplin P, Stavklint H, et al. Changes in dissolved organic matter during the treatment processes of a drinking water plant in sweden and formation of previously unknown disinfection byproducts. Environmental Science & Technology, 2014, 48: 12714-12722

[22] He C, Zhang YH, Li YY, et al. In-house standard method for molecular characterization of dissolved organic matter by ft-icr mass spectrometry. Acs Omega, 2020, 5: 11730-11736

[23] Pelletier GJ, Lewis E, Wallace DWR. Co2sys.Xls: A calculator for the co2 system in seawater for microsoft excel/vba, version 24. Washington State Department of Ecology/Brookhaven National Laboratory, Olympia, WA/Upton, NY, USA, 2015,

[24] Liang YT, Zhang YY, Zhang Y, et al. Distributions and relationships of virio-and picoplankton in the epi-, meso- and bathypelagic zones of the western pacific ocean. Fems Microbiol Ecol, 2017, 93:

[25] Zhao Z, Gonsior M, Schmitt-Kopplin P, et al. Microbial transformation of virus-induced dissolved organic matter from picocyanobacteria: Coupling of bacterial diversity and dom chemodiversity. The ISME Journal, 2019, 13: 2551-2565

[26] Harder T, Lau SCK, Dobretsov S, et al. A distinctive epibiotic bacterial community on the soft coral dendronephthya sp. And antibacterial activity of coral tissue extracts suggest a chemical mechanism against bacterial epibiosis. Fems Microbiol Ecol, 2003, 43: 337-347

[27] Edgar RC. Unoise2: Improved error-correction for illumina 16s and its amplicon sequencing. bioRxiv, 2016, 081257

[28] Quast C, Pruesse E, Yilmaz P, et al. The silva ribosomal rna gene database project: Improved data processing and web-based tools. Nucleic Acids Research, 2012, 41: D590-D596

[29] Bolger AM, Lohse M, Usadel B. Trimmomatic: A flexible trimmer for illumina sequence data. Bioinformatics, 2014, 30: 2114-2120

[30] Li D, Liu C-M, Luo R, et al. Megahit: An ultra-fast single-node solution for large and complex metagenomics assembly via succinct de bruijn graph. Bioinformatics, 2015, 31: 1674-1676

[31] Hyatt D, Chen G-L, LoCascio PF, et al. Prodigal: Prokaryotic gene recognition and translation initiation site identification. BMC bioinformatics, 2010, 11: 1-11

[32] Buchfink B, Xie C, Huson DH. Fast and sensitive protein alignment using diamond. Nature Methods, 2015, 12: 59-60

[33] Zhang H, Yohe T, Huang L, et al. Dbcan2: A meta server for automated carbohydrate-active enzyme annotation. Nucleic Acids Res, 2018, 46: W95-W101

[34] Love MI, Huber W, Anders S. Moderated estimation of fold change and dispersion for rna-seq data with deseq2. Genome Biol, 2014, 15: 550

[35] Uritskiy GV, DiRuggiero J, Taylor J. Metawrap-a flexible pipeline for genome-resolved metagenomic data analysis. Microbiome, 2018, 6: 158

[36] Parks DH, Imelfort M, Skennerton CT, et al. Checkm: Assessing the quality of microbial genomes recovered from isolates, single cells, and metagenomes. Genome Res, 2015, 25: 1043-1055

[37] Parks DH, Chuvochina M, Rinke C, et al. Gtdb: An ongoing census of bacterial and archaeal diversity through a phylogenetically consistent, rank normalized and complete genome-based taxonomy. Nucleic Acids Res, 2022, 50: D785-d794

[38] Letunic I, Bork P. Interactive tree of life (itol) v3: An online tool for the display and annotation of phylogenetic and other trees. Nucleic Acids Res, 2016, 44: W242-245

[39] Langfelder P, Horvath S. Wgcna: An r package for weighted correlation network analysis. BMC bioinformatics, 2008, 9: 1-13

[40] Fasching C, Akotoye C, Bižić M, et al. Linking stream microbial community functional genes to dissolved organic matter and inorganic nutrients. Limnol Oceanogr, 2020, 65: S71-S87
