# Supplementary material for: Fate and carbon sequestration potential of sunken macroalgae in coastal oceans from long-term microbial degradation perspective
Source: Natl Sci Rev. 2025 Jul 8;12(8):nwaf273. doi: 10.1093/nsr/nwaf273 (PMC12365756; doi:10.1093/nsr/nwaf273)
Supplement: nwaf273_Supplemental_Files [file nwaf273_supplemental_files.zip › Table_S2.pdf]

Table S2 KEGG pathways in each sample on the days 0, 5 and 720 of the long-term degradation

KEGG_Pathways	seawater_1	seawater_2	seawater_3	ulva_5th_1	ulva_5th_2	ulva_5th_3	ulva_720th_1	ulva_720th_2	ulva_720_3	control_5th_1	control_5th_2	control_5th_3	control_720_1	level1	level2	level3
ko00010	11.1497076	11.07762384	10.71982475	4.740591636	5.857608185	7.454884831	4.868363306	1.60210011	4.250345488	3.556365927	2.977025143	2.530965847	18.47160093	Metabolism	Carbohydrate metabolism	Glycolysis / Gluconeogenesis
ko00020	68.27985649	70.84747139	59.53575904	43.86364383	48.31528956	44.82161466	44.19434247	39.2503369	42.918681	49.91758552	47.0993197	44.01566752	53.74679894	Metabolism	Carbohydrate metabolism	Citrate cycle (TCA cycle)
ko00030	174.6090976	181.0285201	150.5301803	91.1984307	105.6105729	99.49817513	89.29656967	105.490126	98.71058948	99.26573176	91.23092068	92.52631224	75.37207578	Metabolism	Carbohydrate metabolism	Pentose phosphate pathway
ko00040	88.38347531	87.7063884	79.65939973	123.6722155	129.096416	155.4178273	39.84381155	41.261208	41.27054679	66.10249508	65.39241792	57.90645105	39.61726539	Metabolism	Carbohydrate metabolism	Pentose and glucuronate interconversions
ko00051	88.98164682	92.42029102	89.98496946	66.85525225	77.84491626	95.46089107	39.2337306	46.75770746	45.31419148	57.42067167	52.80808925	50.62614934	39.65990134	Metabolism	Carbohydrate metabolism	Fructose and mannose metabolism
ko00052	60.81431202	64.02855799	51.88096774	35.62017834	43.23976834	35.44510308	24.10506598	38.84405076	33.27714559	38.31455769	34.39668492	31.35679644	11.75524822	Metabolism	Carbohydrate metabolism	Galactose metabolism
ko00053	21.49377389	21.65590401	18.72686839	22.71284493	35.73119077	41.60013971	21.01062391	26.4085662	23.57257738	17.97752033	17.10839018	14.13570125	13.4399296	Metabolism	Carbohydrate metabolism	Ascorbate and aldarate metabolism
ko00061	57.63081472	60.11211116	51.21636607	40.61109576	38.92564208	45.22954766	36.74457721	38.85043692	37.07353108	44.39729186	41.93327016	50.45284306	50.0613297	Metabolism	Lipid metabolism	Fatty acid biosynthesis
ko00071	18.35882716	17.92531098	39.3590107	13.36932198	9.507763718	11.12440336	19.33187261	13.09074725	16.40665101	9.822004114	6.985040527	9.824592781	11.2824623	Metabolism	Lipid metabolism	Fatty acid degradation
ko00073	0	0	0	0	0	0	0	0	0	0.03422619	0.014880952	0.010416667	0.022452504	Metabolism	Lipid metabolism	Cutin, suberine and wax biosynthesis
ko00100	7.348416896	7.821583143	6.875060636	1.971560095	1.315215399	2.007584087	4.394343736	2.31428317	5.079936456	8.266673625	9.680018762	9.858642875	7.308219073	Metabolism	Lipid metabolism	Steroid biosynthesis
ko00120	0.004065041	0	0	0.244277062	0.422933865	0.97413832	0	0	0	0.003236246	0.042978925	0.017878286	0.002645503	Metabolism	Lipid metabolism	Primary bile acid biosynthesis
ko00121	11.01255532	12.08012265	6.472071238	5.507963071	4.998772055	5.614760254	2.554468231	1.010307006	1.115747595	4.450961531	4.916969123	6.200082546	3.076547955	Metabolism	Lipid metabolism	Secondary bile acid biosynthesis
ko00130	245.1560225	263.7304475	228.9731486	104.6097155	107.9176854	131.0767306	209.895526	201.8136557	200.9328099	157.9790393	141.3183453	160.5633282	251.616734	Metabolism	Metabolism of cofactors and vitam	Ubiquinone and other terpenoid-quinone biosynthesis
ko00140	2.1866244	2.143455247	1.969053564	0.427693395	1.281077883	0.656021604	5.009730385	3.722341219	2.555141498	3.318534158	1.405099734	3.919443274	4.744644755	Metabolism	Lipid metabolism	Steroid hormone biosynthesis
ko00190	447.7429648	508.0564433	418.7147452	224.1618755	279.3988407	269.396148	712.3845314	639.8595143	655.3385598	598.5192354	519.6935429	509.7325442	855.6929429	Metabolism	Energy metabolism	Oxidative phosphorylation
ko00195	13.84844264	13.68305874	15.64326	2.633539533	2.336903082	3.324431457	16.46705781	18.55214749	21.24696192	21.73605296	16.1077302	17.31668701	14.87695341	Metabolism	Energy metabolism	Photosynthesis
ko00196	0.451562885	0.499718341	0.427012219	0.13889909	0.022143765	0.053415209	0.081693112	0.206106258	0.002109705	0.113486411	0.107810384	0.183443931	0.003508772	Metabolism	Energy metabolism	Photosynthesis - antenna proteins
ko00220	184.4602786	194.0313427	167.9426423	58.3920365	65.03625264	65.07031076	98.80425771	97.64257698	98.73906489	107.3316881	95.34213244	94.50002505	115.7625529	Metabolism	Amino acid metabolism	Arginine biosynthesis
ko00230	681.9591143	724.3063705	661.536898	350.322541	350.0301557	366.5003809	432.0907919	420.9317754	400.1163749	427.8004855	398.0974656	420.4404519	431.8478295	Metabolism	Nucleotide metabolism	Purine metabolism
ko00232	0	0	0	0	0	0	0.220032392	0.379486027	0.154954955	0	0	0	0	Metabolism	Biosynthesis of other secondary me	Caffeine metabolism
ko00240	349.5295303	369.688575	320.0456013	206.2390719	288.6382204	215.4651579	299.1794715	301.9552659	281.4700993	298.1210912	265.9121915	274.6286361	320.743633	Metabolism	Nucleotide metabolism	Pyrimidine metabolism
ko00250	40.87692666	46.27291328	26.09874682	28.41312548	19.44839871	27.8313493	89.64075858	108.403149	96.12330225	38.29828344	32.66869346	37.63808584	87.62820306	Metabolism	Amino acid metabolism	Alanine, aspartate and glutamate metabolism
ko00253	0	0	0.002583979	0.031007752	0.010335917	0.007751938	0	0	0	0	0	0	0	Metabolism	Metabolism of terpenoids and poly	Tetracycline biosynthesis
ko00260	370.3729467	373.1663282	354.7263193	144.1465938	134.8960698	118.9970176	165.6300331	169.0820916	143.1268305	384.7180734	357.1463638	325.5446219	144.9765645	Metabolism	Amino acid metabolism	Glycine, serine and threonine metabolism
ko00261	0.022334188	0.012114146	1.331998474	0.143643598	0.132209954	0.64250691	0.319664469	0.093581116	0.12994184	0.164449618	0.112451023	0.128629545	0.124687569	Metabolism	Biosynthesis of other secondary me	Monobactam biosynthesis
ko00270	217.4549418	225.8356769	220.9861519	96.92386368	111.8169773	112.2796755	213.4009227	225.7023588	211.1325076	171.3760672	158.7534184	164.7718483	245.6013014	Metabolism	Amino acid metabolism	Cysteine and methionine metabolism
ko00280	143.6101036	155.7050975	159.3241775	62.24031679	55.69679761	51.63495792	68.7413329	83.7465889	74.84799457	87.47920421	80.63000481	89.49484077	93.8814519	Metabolism	Amino acid metabolism	Valine, leucine and isoleucine degradation
ko00281	9.658963853	10.85357925	49.93237108	3.800924397	4.416056893	2.895596497	14.09342352	13.00812081	5.264837157	11.24104316	11.73258285	14.31712406	18.40560894	Metabolism	Metabolism of terpenoids and poly	Geraniol degradation
ko00290	1.417905932	1.372300212	5.983408838	0.885800232	3.256884787	2.256950099	3.492971374	1.216297836	2.83920476	1.572259746	1.858495347	3.401567907	1.10661538	Metabolism	Amino acid metabolism	Valine, leucine and isoleucine biosynthesis
ko00300	150.8677206	160.1927687	140.6299339	72.74451097	77.99114697	75.00704265	100.7350054	99.75306837	100.3533617	128.5080006	114.1057332	117.1052186	116.2661015	Metabolism	Amino acid metabolism	Lysine biosynthesis
ko00310	25.40467999	25.12767623	26.18413485	14.52936841	13.79422471	11.59279561	34.18368785	28.00245274	27.4381545	50.89637524	47.62507446	42.58183548	35.4700809	Metabolism	Amino acid metabolism	Lysine degradation
ko00311	23.73880397	23.51493959	19.23639071	19.32290816	14.32289491	14.44074486	23.20172997	29.45368717	24.87708771	24.28184282	17.78651906	20.36872442	18.05828606	Metabolism	Biosynthesis of other secondary me	Penicillin and cephalosporin biosynthesis
ko00330	274.9373874	285.4560529	294.6880778	164.3439381	157.5877057	149.441724	260.2759871	288.6924858	256.1758687	237.7616647	227.8217914	225.469768	231.8321641	Metabolism	Amino acid metabolism	Arginine and proline metabolism
ko00332	0	0.007440476	0	0	0	0	0	0	0.119125683	0	0.001488095	0	0.116685076	Metabolism	Biosynthesis of other secondary me	Carbapenem biosynthesis
ko00333	0.033670034	0.026936027	0.006734007	0.899533236	0.34155054	0.150738474	0.05922891	0	0	0.2491896	0.013850493	0	0.097542167	Metabolism	Biosynthesis of other secondary me	Prodigiosin biosyntheses
ko00340	274.3571567	289.1822918	254.8644304	111.8698701	123.8393898	123.9203112	261.5086809	276.556092	258.5583579	195.9957694	181.1170482	186.4636049	259.6081551	Metabolism	Amino acid metabolism	Histidine metabolism
ko00350	37.78946927	38.74823801	34.23260377	20.04486768	20.31547818	17.10418352	38.69230522	54.35826629	47.27771245	50.63031342	45.2657386	43.07722797	44.78221101	Metabolism	Amino acid metabolism	Tyrosine metabolism
ko00360	133.6578097	142.5218517	140.9986415	43.15503922	35.98519955	32.77253432	82.59088336	96.98327481	79.71451761	100.425223	88.39597997	99.10255146	99.43026541	Metabolism	Amino acid metabolism	Phenylalanine metabolism
ko00361	4.23054947	4.109909182	3.994808843	0.598048207	0.684104872	0.671677657	10.25790553	15.87725061	12.63026858	5.651243916	5.878344026	5.943085238	14.34062471	Metabolism	Xenobiotics biodegradation and me	Chlorocyclohexane and chlorobenzene degradation
ko00362	77.41483705	81.97149057	69.01217366	44.26308806	44.67694984	27.74254687	78.62130029	117.6488777	90.84804987	50.88393082	47.13546033	50.15703523	108.9846376	Metabolism	Xenobiotics biodegradation and me	Benzoate degradation
ko00363	5.818777396	5.801971339	5.507818017	0.99593911	1.153507622	0.569773768	3.116383703	3.386672625	3.035868114	3.541993386	4.493696176	5.211243779	8.84265124	Metabolism	Xenobiotics biodegradation and me	Bisphenol degradation
ko00365	2.351290975	2.697054352	21.6467374	0.327700961	0.601902728	0.54430558	29.81215483	43.81656185	32.38869882	4.955248363	5.508011862	7.562820347	28.44838552	Metabolism	Xenobiotics biodegradation and me	Furfural degradation
ko00380	27.60555058	26.29786664	20.48518615	24.8974266	17.67540049	32.96310908	25.3851104	35.29624984	26.34917532	24.68442592	20.74294549	27.3240516	15.87605169	Metabolism	Amino acid metabolism	Tryptophan metabolism
ko00400	331.5739111	348.7192571	311.4929483	143.7993979	157.0309125	163.0157789	245.7619675	242.8832542	242.2906352	270.9518863	245.5705144	255.9617525	259.596453	Metabolism	Amino acid metabolism	Phenylalanine, tyrosine and tryptophan biosynthesis
ko00404	15.81474367	14.88972756	8.844963552	26.67913944	46.00057948	32.34403418	0.932857824	0.658560304	0.764086563	5.729219037	3.759823586	2.698330156	0.666090095	Metabolism	Biosynthesis of other secondary me	Staurosporine biosynthesis
ko00405	9.057334607	9.4914707	7.849457846	2.557148316	1.896007086	1.290000219	0.855163462	2.890798666	1.511043564	6.586436589	5.368500208	4.871468947	0.865325558	Metabolism	Biosynthesis of other secondary me	Phenazine biosynthesis
ko00430	21.96620628	22.73775142	20.05772645	9.426073427	8.608698116	7.141804482	4.89726821	5.981337786	6.693185293	30.15629496	27.19531431	27.42506375	2.706843	Metabolism	Metabolism of other amino acids	Taurine and hypotaurine metabolism
ko00440	57.82997393	59.0578	48.90208068	43.80668358	44.64428687	30.99796992	40.79557027	56.56051552	53.0348234	51.25260875	43.85269111	38.061187	38.45332279	Metabolism	Metabolism of other amino acids	Phosphonate and phosphinate metabolism
ko00450	83.57117351	88.13438426	79.61175641	52.91382018	48.68935411	57.08264757	88.78705316	93.98705566	85.27920192	86.80616007	78.87319608	81.07206892	106.3912048	Metabolism	Metabolism of other amino acids	Selenocompound metabolism
ko00460	0.000719942	0.03256705	0	0.28844295	0.088510139	0.041042599	0.569081206	0.107724174	0.233794812	0.08595203	0.023238279	0	2.840889812	Metabolism	Metabolism of other amino acids	Cyanoamino acid metabolism
ko00471	14.24662823	16.33535268	15.02314998	8.082046912	8.059235477	9.163336278	11.39200777	11.81161662	9.799553244	8.968192451	7.605486389	8.268356671	21.18838939	Metabolism	Metabolism of other amino acids	D-Glutamine and D-glutamate metabolism
ko00472	4.445476011	4.498923007	3.881264692	4.029406107	3.182183887	2.338922912	2.054518969	2.356656458	1.849661184	4.083692372	3.883659851	3.710141504	2.791367866	Metabolism	Metabolism of other amino acids	D-Arginine and D-ornithine metabolism
ko00480	162.2686172	165.5584785	139.507552	89.77579326	91.05280994	90.61997929	87.14589942	98.54920413	89.3663193	98.93210068	88.97404243	92.31063476	92.60019013	Metabolism	Metabolism of other amino acids	Glutathione metabolism
ko00500	115.9135079	113.8376837	89.90612469	89.13944248	78.51137351	112.4644476	78.01248354	62.94883726	68.4724292	40.15802495	35.78843261	32.30921821	113.9423784	Metabolism	Carbohydrate metabolism	Starch and sucrose metabolism
ko00510	7.555440844	6.896314711	8.881613454	7.354344373	8.744508102	18.11699583	32.97805579	43.61953987	39.64761283	16.76263243	14.8196911	19.84487121	29.91829988	Metabolism	Glycan biosynthesis and metabolis	N-Glycan biosynthesis
ko00511	0.681413026	0.856534397	0.732499461	15.76816623	9.922286328	8.070018552	2.672509292	1.577041154	0.480041602	1.559234125	2.776577892	1.535622618	3.043430795	Metabolism	Glycan biosynthesis and metabolis	Other glycan degradation
ko00513	0.289065586	0.312644226	0.266353981	0.627292675	0.261420438	1.760935006	0.07254902	0.08909228	0.055498906	0.07559277	0.125255416	0.111627655	0.006952193	Metabolism	Glycan biosynthesis and metabolis	Various types of N-glycan biosynthesis
ko00514	0.89920119	0.883338809	0.956943807	0.317793932	0.79483649	1.148611967	0.603914434	0.687686394	0.636557651	0.059139381	0.149115247	0.060484759	0.362324855	Metabolism	Glycan biosynthesis and metabolis	Other types of O-glycan biosynthesis
ko00515	0.209972662	0.219337578	0.164917945	0.031521888	0.230203456	0.14393173	0.102963687	0.058423549	0.232970569	0.203425256	0.235996952	0.161897457	0.039408867	Metabolism	Glycan biosynthesis and metabolis	Mannose type O-glycan biosynthesis
ko00520	251.6127254	271.2101891	226.0776764	154.9772776	172.621508	197.2997698	244.2258562	284.1308352	259.1874166	207.71405	179.4468556	185.7597629	247.7454012	Metabolism	Carbohydrate metabolism	Amino sugar and nucleotide sugar metabolism
ko00521	0.084622736	0.098648306	0.0812847	0.021511042	0.16306831	0.087584425	0.137034354	0	0.021276596	0.03325074	0.050851561	0.023698524	0.021359613	Metabolism	Biosynthesis of other secondary me	Streptomycin biosynthesis
ko00523	0.344668491	0.332313972	0.279901429	0.102417032	0.10673243	0.199291318	5.497285657	7.522636014	2.189113098	0.686696888	0.649059332	0.505643476	4.097084565	Metabolism	Metabolism of terpenoids and poly	Polyketide sugar unit biosynthesis
ko00525	0.025909038	0.081183305	4.436737088	0.312330844	0.183500078	0.598853987	0.142227158	0.330533774	0.074304708	0.040383062	0.098894035	0.419703991	0.389700629	Metabolism	Biosynthesis of other secondary me	Acarbose and validamycin biosynthesis
ko00532	0.121981241	0.095884019	0.112882426	0.293292675	0.436232009	0.346332764	0.949488201	0.478459724	0.355774912	0.156325359	0.08440402	0.122663039	0.369409557	Metabolism	Glycan biosynthesis and metabolis	Glycosaminoglycan biosynthesis - chondroitin sulfate / dermatan sulfate
ko00533	0	0	0	0	0	0	0	0	0.038022814	0	0	0	0	Metabolism	Glycan biosynthesis and metabolis	Glycosaminoglycan biosynthesis - keratan sulfate
ko00534	0.107074654	0.072353904	0.072678901	0.407593177	0.347928528	1.125801447	0.47269856	0.520820907	0.763084757	0.34243955	0.147949646	0.161520614	0.200738069	Metabolism	Glycan biosynthesis and metabolis	Glycosaminoglycan biosynthesis - heparan sulfate / heparin
ko00540	406.1703335	424.1577234	379.4237619	193.6315955	218.6344243	233.353543	272.1355579	287.3212338	291.8183868	302.5607224	282.6995909	294.0888038	211.2275793	Metabolism	Glycan biosynthesis and metabolis	Lipopolysaccharide biosynthesis
ko00550	125.1421643	132.1060343	137.7376618	64.50445136	74.63768207	73.30792351	66.86032215	52.19371943	58.66055686	83.91459878	74.83904683	84.68670318	81.98507834	Metabolism	Glycan biosynthesis and metabolis	Peptidoglycan biosynthesis
ko00561	38.0922862	43.48766426	84.89060899	21.57192538	16.87462145	18.58241293	47.02929964	35.54824979	33.81481702	39.50534491	37.39191576	38.57701674	58.15518739	Metabolism	Lipid metabolism	Glycerolipid metabolism
ko00562	46.03193959	48.41114151	43.16028375	50.67191824	54.89498085	48.76502402	11.4015024	11.93896806	10.64366569	49.16551089	44.78754229	39.72267427	10.95663789	Metabolism	Carbohydrate metabolism	Inositol phosphate metabolism
ko00563	0.96210076	0.980092191	1.050394276	0.628013348	0.789440266	1.378498641	0.031134352	0.012256064	0.066023062	0.470541897	0.862095249	5.92286491	0.148205374	Metabolism	Glycan biosynthesis and metabolis	Glycosylphosphatidylinositol(GPI)-anchor biosynthesis
ko00564	199.1730658	202.4401935	210.7756015	109.803856	108.0745183	122.2261629	190.5148265	168.560137	177.9725029	167.44112	152.797084	176.3900443	190.5753805	Metabolism	Lipid metabolism	Glycerophospholipid metabolism
ko00565	0.288287904	0.199474308	0.303768148	0.240660285	0.23178677	0.337030707	1.243699281	0.441754517	0.172596944	0.409411229	0.443901594	0.407735761	0.168664909	Metabolism	Lipid metabolism	Ether lipid metabolism
ko00571	11.17117422	12.0809911	10.37847702	7.822513595	10.86512325	13.06932173	21.44784233	11.08570253	13.00095485	28.80234182	22.96177245	19.93350277	45.45667047	Metabolism	Glycan biosynthesis and metabolis	Lipoarabinomannan (LAM) biosynthesis
ko00572	21.06101335	22.71826587	15.62245418	11.01247424	9.572055287	7.84766542	14.6131371	10.5534935	10.67001096	15.34183165	13.12294261	11.38371591	21.97755942	Metabolism	Glycan biosynthesis and metabolis	Arabinogalactan biosynthesis - Mycobacterium
ko00590	2.227375441	2.032355005	1.842757602	2.823274971	2.12056423	2.63915751	1.741510797	0.727523586	0.450929958	1.071255817	1.327791392	0.967184128	0.59429106	Metabolism	Lipid metabolism	Arachidonic acid metabolism
ko00591	1.313751172	1.12615035	0.937140387	6.246637696	3.007103313	7.546356156	3.320031297	0.61953711	1.595245711	2.764340731	1.566321382	1.552058717	8.160524916	Metabolism	Lipid metabolism	Linoleic acid metabolism
ko00592	0.153426571	0.115423915	0.115077207	0.434124135	0.197253314	0.135909436	1.35273096	0.605905941	0.624886637	0.188167301	0.216716327	1.410188172	0.375430038	Metabolism	Lipid metabolism	alpha-Linolenic acid metabolism
ko00600	3.633632618	2.988809623	1.852708941	2.057318492	4.092864063	3.411119771	1.271135319	1.189235412	0.680770054	1.950875368	1.212360313	0.955672128	0.737432203	Metabolism	Lipid metabolism	Sphingolipid metabolism

ko00601	0.19072188	0.26057353	0.198843771	0.049466693	0.094933022	0.122388987	0.058748404	0	0.146202264	0.451270219	0.361541518	0.413576542	0	Metabolism	Glycan biosynthesis and metabolis	Glycosphingolipid biosynthesis - lacto and neolacto series
ko00604	0.066584967	0.021486928	0.037908497	0.103425458	0.166840007	0.295368031	0	0	0	0	0.044871795	0.012820513	0	Metabolism	Glycan biosynthesis and metabolis	Glycosphingolipid biosynthesis - ganglio series
ko00620	138.2664245	148.9484835	136.0529847	70.98094851	70.53838661	76.06637103	87.01257194	97.0785929	86.8468317	77.87478596	77.21717113	80.6331935	113.3347025	Metabolism	Carbohydrate metabolism	Pyruvate metabolism
ko00621	0.001801802	0.017921147	0.023613435	0	0.011914537	0.007286845	1.628139091	4.894185338	2.930228961	0.035772071	0.059924479	0.444533857	1.610104707	Metabolism	Xenobiotics biodegradation and me	Dioxin degradation
ko00622	0.149619564	0.277339371	0.199509458	0.004761905	0.001587302	0.001587302	3.009476583	3.643135311	3.128273482	0.26333954	0.381492017	0.526173233	7.039528006	Metabolism	Xenobiotics biodegradation and me	Xylene degradation
ko00623	0.490582771	0.540620801	0.51663164	3.445948638	2.059375833	2.097965332	7.931349099	16.56356579	11.08405429	0.740682302	0.731042157	1.019899957	16.28938606	Metabolism	Xenobiotics biodegradation and me	Toluene degradation
ko00624	8.345982637	8.612874424	8.196799369	2.936597043	4.58691489	2.247675711	14.75258561	20.2476226	18.84320468	6.696491945	6.294576943	11.65121112	6.237067129	Metabolism	Xenobiotics biodegradation and me	Polycyclic aromatic hydrocarbon degradation
ko00625	0	0.001984127	0.001836547	0	0.000886525	0	0.898148806	0.655388805	0.562396743	0	0.025859676	0.050199516	2.81711044	Metabolism	Xenobiotics biodegradation and me	Chloroalkane and chloroalkene degradation
ko00626	0.235641542	0.328885268	0.333330007	0.006633499	0.003384095	0	1.961842255	3.288503261	4.29607921	0.286528766	0.271837379	0.257670991	2.231794633	Metabolism	Xenobiotics biodegradation and me	Naphthalene degradation
ko00627	25.19677946	27.80821289	24.11824011	11.29720151	15.14930837	9.983121733	48.5458151	69.64921482	51.63750265	23.01374556	21.30472288	22.40714644	42.46745468	Metabolism	Xenobiotics biodegradation and me	Aminobenzoate degradation
ko00630	219.2420192	236.28669	224.8937173	96.40547105	93.6490974	64.16710395	168.0835909	198.0616314	188.2396821	165.1444022	144.7664409	144.1866256	181.310766	Metabolism	Carbohydrate metabolism	Glyoxylate and dicarboxylate metabolism
ko00633	8.837313262	8.271792445	19.91632883	10.84247797	8.483415392	14.96222787	5.322701361	6.998725468	6.836537251	4.764496897	4.349607166	7.011009947	7.892480279	Metabolism	Xenobiotics biodegradation and me	Nitrotoluene degradation
ko00640	183.5615186	197.9655132	167.4345726	47.48036693	37.67447188	41.22384182	37.55661478	45.20149788	40.35290442	87.36307832	83.1725483	80.16320414	57.40521937	Metabolism	Carbohydrate metabolism	Propanoate metabolism
ko00642	0.155935844	0.113689483	0.11212682	0.007490637	0.093506577	0.022715508	1.248121988	2.846364298	4.371863145	0.020701267	0.675585566	0.28994842	1.291728526	Metabolism	Xenobiotics biodegradation and me	Ethylbenzene degradation
ko00643	0	0	0	0	0	0	0.011976048	0.061876248	0	0	0	0.007984032	0	Metabolism	Xenobiotics biodegradation and me	Styrene degradation
ko00650	60.77022261	64.59161555	58.62203674	39.32852982	52.38772827	47.62053813	82.2767626	97.26050749	87.79519163	41.03530381	38.0263779	41.19595173	87.82231962	Metabolism	Carbohydrate metabolism	Butanoate metabolism
ko00660	3.110986495	2.954818918	3.42150143	0.349784682	0.144144916	0.100490283	8.969969319	13.90970424	12.67799245	4.821838328	5.046319028	5.38293444	6.40628085	Metabolism	Carbohydrate metabolism	C5-Branched dibasic acid metabolism
ko00670	26.60825713	27.37681035	25.44374383	13.00940201	15.26596381	12.43974392	21.78761958	18.88149075	19.6230901	20.72143124	18.95261403	20.03708857	20.74452159	Metabolism	Metabolism of cofactors and vitam	One carbon pool by folate
ko00680	153.2351037	156.6143145	160.6160089	67.13825901	61.91240976	56.36623902	219.4672246	185.6582047	197.0036258	210.2779596	196.1999042	186.0287933	202.9731072	Metabolism	Energy metabolism	Methane metabolism
ko00710	14.35308335	14.90948253	14.79132349	4.00042764	4.25178324	2.932594047	5.428273471	3.779243092	4.177704741	1.848415849	2.030179804	2.011295848	4.252348025	Metabolism	Energy metabolism	Carbon fixation in photosynthetic organisms
ko00720	26.28886792	26.44990751	25.43824975	3.848838276	3.113927531	2.309071899	154.5476769	140.6690668	133.6124538	18.85194211	18.77884223	25.17294186	147.6303733	Metabolism	Energy metabolism	Carbon fixation pathways in prokaryotes
ko00730	130.7322032	144.7449666	142.3429673	76.5621451	87.88624345	116.6995769	147.8003604	146.5710901	151.1253702	85.34288981	78.99984174	78.51708201	144.4545533	Metabolism	Metabolism of cofactors and vitam	Thiamine metabolism
ko00740	117.1170171	125.6926896	99.75365722	45.60059573	49.80171259	48.92045704	131.0203397	123.21621	125.8955934	74.13453718	67.1443294	73.20716946	120.7039143	Metabolism	Metabolism of cofactors and vitam	Riboflavin metabolism
ko00750	123.3012631	126.9660071	120.7010172	50.61104743	51.97458015	50.21357877	72.79800764	68.97827041	69.06992502	81.19790241	72.66435388	80.16260248	86.8290851	Metabolism	Metabolism of cofactors and vitam	Vitamin B6 metabolism
ko00760	278.5869838	288.7174338	267.5977113	129.2207508	142.6386741	135.509456	276.1805797	285.6678983	271.0418011	235.0663169	207.5426938	220.4563341	281.8126562	Metabolism	Metabolism of cofactors and vitam	Nicotinate and nicotinamide metabolism
ko00770	232.4002381	240.2949876	202.0683015	86.5960314	89.76267647	94.32782476	159.7351994	178.4343357	160.5386076	177.8796735	156.6682001	158.5251925	184.1215384	Metabolism	Metabolism of cofactors and vitam	Pantothenate and CoA biosynthesis
ko00780	112.3211649	119.7059339	94.29960526	49.18177993	54.87062765	75.14824386	101.5992598	97.08090842	109.8350852	57.01820747	48.91283983	56.30768974	137.9991089	Metabolism	Metabolism of cofactors and vitam	Biotin metabolism
ko00785	51.69340857	52.66881821	49.32717168	25.13197234	27.83742688	33.99884012	39.77806683	44.27868483	42.4552472	45.77735317	42.2743957	44.38089041	43.4181237	Metabolism	Metabolism of cofactors and vitam	Lipoic acid metabolism
ko00790	360.2540734	381.6378978	323.7533596	159.3794224	178.1728655	202.2239178	305.6310133	278.7723047	294.1451447	267.6375687	241.3600313	264.4560168	336.1767805	Metabolism	Metabolism of cofactors and vitam	Folate biosynthesis
ko00791	0.72743561	0.788548576	1.131052688	3.340783816	2.281568516	1.382449421	7.274967422	8.444852672	6.346010635	3.304157696	2.978959291	2.734848189	9.039029415	Metabolism	Xenobiotics biodegradation and me	Atrazine degradation
ko00830	2.632686866	2.71480025	8.92117083	2.618227247	2.785997324	2.945018284	5.787213635	3.33727927	4.936537485	3.730815875	3.832383936	4.147484041	2.802834193	Metabolism	Metabolism of cofactors and vitam	Retinol metabolism
ko00860	687.9555423	717.6834816	641.1197042	292.3397094	324.9661013	306.8224325	604.7600879	662.7140757	603.9140778	442.7864889	404.1242047	397.977229	581.5630314	Metabolism	Metabolism of cofactors and vitam	Porphyrin and chlorophyll metabolism
ko00900	254.4462864	258.763418	231.5254108	103.5510361	109.9116626	118.1741109	196.3036383	187.1350819	191.0380372	217.7083755	188.2198853	193.473348	215.2918027	Metabolism	Metabolism of terpenoids and poly	Terpenoid backbone biosynthesis
ko00902	0	0.000848176	0.004819785	0	0	0	0.260933101	0.161802586	0.244201894	0.00877193	0.005582137	0.0485488	0.38256409	Metabolism	Metabolism of terpenoids and poly	Monoterpenoid biosynthesis
ko00903	0.724942261	0.765388333	0.704562722	0.062320269	0.045836204	0.040198639	4.741151886	6.516214175	4.633451307	1.513300202	1.84607289	2.332267527	8.815939728	Metabolism	Metabolism of terpenoids and poly	Limonene and pinene degradation
ko00904	0.016666667	0.041666667	0.008333333	0	0	0	0.033755274	0.004219409	0	0	0	0.004166667	0	Metabolism	Metabolism of terpenoids and poly	Diterpenoid biosynthesis
ko00905	0.023569024	0.03030303	0.038720539	0	0	0	0	0	0	0	0	0.006734007	0	Metabolism	Metabolism of terpenoids and poly	Brassinosteroid biosynthesis
ko00906	95.80104786	93.41986123	78.37231915	28.88420951	33.74117821	40.25681971	17.02205544	11.49182937	11.03604586	71.54629464	62.92103899	55.35279783	23.63918808	Metabolism	Metabolism of terpenoids and poly	Carotenoid biosynthesis
ko00908	30.26597489	31.75153646	26.74562173	13.00678956	14.90921429	17.37786876	17.35927695	16.42517048	16.20971561	26.05161073	23.12114815	23.95938627	21.43228742	Metabolism	Metabolism of terpenoids and poly	Zeatin biosynthesis
ko00909	0.97177547	0.920171694	1.163446777	0.094367323	0.028006622	0.020563918	11.77541798	13.98649601	12.86301194	2.297220005	2.427143888	2.113236594	5.01567233	Metabolism	Metabolism of terpenoids and poly	Sesquiterpenoid and triterpenoid biosynthesis
ko00910	78.42058503	89.71237002	96.54093989	85.49236749	87.75197748	97.73445213	120.0223354	110.5286969	110.2493536	43.86877355	44.71819994	52.7570484	160.4754287	Metabolism	Energy metabolism	Nitrogen metabolism
ko00920	362.4320232	399.7400269	283.349314	139.1983236	145.5191327	135.5074258	215.9709145	238.0678848	237.6629597	200.3546242	188.4832134	181.8524327	213.9508002	Metabolism	Energy metabolism	Sulfur metabolism
ko00930	2.442053015	2.274871871	3.573646983	2.617406237	3.42627122	4.85462688	9.025330524	13.52576476	10.02151359	14.64444051	19.65279494	15.87442204	16.53731544	Metabolism	Xenobiotics biodegradation and me	Caprolactam degradation
ko00940	0.032183908	0.033818085	0.042742637	0	0	0	0.641204371	0.208522856	1.069598298	0	0.033333333	0.023058927	3.869594347	Metabolism	Biosynthesis of other secondary me	Phenylpropanoid biosynthesis
ko00941	0.073093981	0.100163997	0.126077354	0	0	0	0	0	0	0.00462963	0.057966135	0.044213412	0	Metabolism	Biosynthesis of other secondary me	Flavonoid biosynthesis
ko00943	0.20627263	0.228347883	0.223318167	0.275858319	0.828811821	1.16173775	3.484664628	4.795899142	2.234998928	0.884578556	0.465415118	0.402204279	3.196241661	Metabolism	Biosynthesis of other secondary me	Isoflavonoid biosynthesis
ko00960	0.825209764	0.857950839	0.815143294	0.288566141	0.153182155	0.061836625	5.296827242	4.41927246	3.234156216	2.072215856	0.686721696	0.941055569	3.070979042	Metabolism	Biosynthesis of other secondary me	Tropane, piperidine and pyridine alkaloid biosynthesis
ko00965	12.95071171	17.13364414	14.30673589	2.760633903	3.757696287	2.073924731	2.349114	5.189680711	3.0911385	1.502898952	1.9356962	2.382038024	4.972761938	Metabolism	Biosynthesis of other secondary me	Betalain biosynthesis
ko00970	1126.587889	1200.662714	1027.816651	529.2799342	558.145078	597.1314975	826.8859167	785.5887941	768.6924524	878.0881494	803.0610325	810.6282831	910.9423709	Genetic Informat	Translation	Aminoacyl-tRNA biosynthesis
ko00980	0.040540541	0.036036036	0.029279279	0	0	0	0.017687732	0.046841348	0.061286921	0.021306341	0.12059014	0.050465826	0.092597872	Metabolism	Xenobiotics biodegradation and me	Metabolism of xenobiotics by cytochrome P450
ko00981	0	0.019607843	0	0	0	0	0.045213839	0	0	0	0.04589042	0.003565062	0.07862654	Metabolism	Metabolism of terpenoids and poly	Insect hormone biosynthesis
ko00982	0.043604272	0.045660331	0.063722882	1.050617743	0.264215943	0.294350559	9.281831646	0.724629947	5.031670073	0.23430335	0.20146652	0.291798805	0.953671592	Metabolism	Xenobiotics biodegradation and me	Drug metabolism - cytochrome P450
ko00983	7.487671845	8.084800505	7.641705366	1.668184421	0.92259584	0.886532045	0.843577406	0.855904129	0.458101617	1.572919939	1.86673012	2.340402809	0.745263611	Metabolism	Xenobiotics biodegradation and me	Drug metabolism - other enzymes
ko00984	32.67769724	30.75313432	20.94517473	1.834457941	2.181391671	1.300067024	25.77802773	20.38082134	18.78454171	6.462364799	18.20631331	36.6008325	29.97112255	Metabolism	Xenobiotics biodegradation and me	Steroid degradation
ko00999	0.102756892	0.078254158	0.087719298	0	0	0.001618123	0.659272138	0.1560435	0.099993864	0.012373166	0.069280111	0.041077427	0.072739499	Metabolism	Biosynthesis of other secondary me	Biosynthesis of secondary metabolites - unclassified
ko01040	45.42055708	46.48872395	49.43923712	18.26310918	17.87211703	19.04007319	31.02165461	27.59009446	28.98424987	25.67616398	25.69507289	27.51920548	37.31231763	Metabolism	Lipid metabolism	Biosynthesis of unsaturated fatty acids
ko01051	0.377108644	0.470271505	0.396854809	0.000992063	0	0.003968254	1.009791713	0.227852437	0.310006646	0.479507968	0.488849608	0.211419995	1.379005743	Metabolism	Metabolism of terpenoids and poly	Biosynthesis of ansamycins
ko01052	0.035665295	0.023319616	0.053919753	0.077169312	0.026105442	0.118611111	0.73650046	0.13527422	0.148557047	0.206980215	0.394949924	0.195890958	0.373838617	Metabolism	Metabolism of terpenoids and poly	Type I polyketide structures
ko01053	0.318776122	1.048853638	3.657202514	7.846010194	6.322737242	4.313654282	9.521772128	4.100299494	3.358004942	2.109777814	2.701745527	2.4247698	5.161398674	Metabolism	Metabolism of terpenoids and poly	Biosynthesis of siderophore group nonribosomal peptides
ko01054	0.30465205	0.298113634	0.211239362	4.297949069	1.496914743	1.522908921	0.557142436	0.68297772	0.301268257	2.159407642	1.291861153	0.751368925	0.203526673	Metabolism	Metabolism of terpenoids and poly	Nonribosomal peptide structures
ko01055	0.261303113	0.307159016	0.32190601	0.012457335	0.025157233	0.00137741	0.155279481	0.115616686	0.206987045	0.157109336	0.150742177	0.135812528	0.058840765	Metabolism	Metabolism of terpenoids and poly	Biosynthesis of vancomycin group antibiotics
ko01057	7.905384001	8.117604209	4.257170673	1.791075498	2.812556352	2.181927821	14.95524153	16.84076955	16.17773967	3.669745765	3.360325492	3.300114261	29.54202747	Metabolism	Metabolism of terpenoids and poly	Biosynthesis of type II polyketide products
ko01059	17.19519129	16.41075124	16.12004904	24.89924344	9.417379281	7.56721344	32.90595224	28.25945766	16.84007928	15.51687482	15.75087626	19.67331504	31.21001886	Metabolism	Metabolism of terpenoids and poly	Biosynthesis of enediyne antibiotics
ko01501	32.87057112	36.71340435	30.90308861	16.09447111	19.56566862	16.98019492	22.51213917	21.4158757	22.46368278	25.47920068	24.36825496	27.07373539	18.30950184	Human Diseases	Drug resistance	beta-Lactam resistance
ko01503	54.87319897	56.76773339	50.27523295	36.67335739	42.82487669	37.83748145	33.66716383	30.74198047	33.53655368	29.703223	27.39068736	30.275561	41.25685407	Human Diseases	Drug resistance	Cationic antimicrobial peptide (CAMP) resistance
ko01521	0.100991451	0.102876728	0.073080147	0	0	0	0	0	0	0	0	0.007067138	0	Human Diseases	Drug resistance	EGFR tyrosine kinase inhibitor resistance
ko01522	0.147631849	0.10104587	0.112576691	0.041280233	0.049736316	0.072997038	0	0	0	0.044166323	0.142444203	0.073491217	0	Human Diseases	Drug resistance	Endocrine resistance
ko01524	3.341215752	2.844385934	3.421064292	8.984508913	3.819083135	8.992845122	0.328001469	0.018975244	0.008321895	16.4452945	12.79386579	12.95990728	0.050487266	Human Diseases	Drug resistance	Platinum drug resistance
ko02010	1787.905123	1929.277304	1903.681774	1174.847542	1225.63685	938.960033	1042.182336	1059.958507	1065.687592	1469.668577	1322.972842	1304.747033	1157.960578	Environmental Ir	Membrane transport	ABC transporters
ko02020	726.7341664	764.8875792	727.1400126	447.9684801	471.1744772	455.6302783	508.7167532	501.1069248	501.5231049	488.3577187	442.2353606	452.3368825	537.3300482	Environmental Ir	Signal transduction	Two-component system
ko02024	413.3312736	435.2429607	388.0680728	550.8494522	464.7198162	353.7065511	578.6819333	657.5783641	494.3047878	448.0538998	406.7247182	395.7739629	510.3036773	Cellular Process	Cellular community - prokaryotes	Quorum sensing
ko02025	33.16694892	42.50470394	192.711055	48.46778757	61.74981035	54.24232417	47.75419286	29.21261413	35.27930384	29.55029078	23.95706986	34.55989736	48.97372526	Cellular Process	Cellular community - prokaryotes	Biofilm formation - Pseudomonas aeruginosa
ko02026	82.34687602	84.65595433	104.5095813	61.8556212	62.67564749	53.10302086	63.34410118	67.55978053	63.22364565	61.10664194	56.72754313	67.16906684	77.55430116	Cellular Process	Cellular community - prokaryotes	Biofilm formation - Escherichia coli
ko02030	9.94493796	11.9150719	5.886868456	11.20658654	10.72583879	8.688885425	10.91777034	14.51619885	10.87777079	4.858783816	3.713304637	3.342762286	9.301470882	Cellular Process	Cell motility	Bacterial chemotaxis
ko02040	169.2079295	192.115525	243.4375755	187.7713598	171.2149214	147.4231604	174.8897073	277.2392173	219.1965484	164.8869862	147.9649248	152.1702734	232.1295334	Cellular Process	Cell motility	Flagellar assembly
ko02060	55.04548168	57.47429712	66.43351121	29.58062319	35.34630807	27.96369162	59.33988175	47.50204986	60.06744726	40.65743634	31.61520317	33.27432637	63.26047233	Environmental Ir	Membrane transport	Phosphotransferase system (PTS)
ko03008	20.04965225	19.55758221	18.92101287	5.521857729	11.37775415	13.35825948	54.76851817	45.82881524	47.47095333	10.03645387	11.59908375	14.15002856	47.36226978	Genetic Informat	Translation	Ribosome biogenesis in eukaryotes
ko03010	1132.76951	1158.316694	959.3052394	689.9363523	817.6623	810.2572735	1042.587069	1041.159793	1007.770046	970.9734473	860.3302229	919.4530808	1236.761428	Genetic Informat	Translation	Ribosome
ko03013	48.77229123	49.26865912	45.22306842	26.7552872	23.44212145	32.98109505	93.09052877	81.87784653	82.04465431	29.5052957	28.52192524	39.48927942	101.6074945	Genetic Informat	Translation	RNA transport
ko03015	3.792781078	3.907062242	4.101228114	3.84263701	2.859508571	5.935631968	29.3897527	26.77459114	23.5486014	2.855956486	4.323749652	5.493014741	23.8650805	Genetic Informat	Translation	mRNA surveillance pathway
ko03018	466.5947832	494.2068827	433.8207401	258.9583538	260.1194872	295.701934	248.5197881	227.2253657	222.2481115	322.0791611	293.1989539	295.4142427	278.4564633	Genetic Informat	Folding, sorting and degradation	RNA degradation
ko03020	288.0665835	293.2286301	271.9713405	211.9078835	236.3267127	226.2327768	252.7490875	231.4434737	237.2906535	296.6655652	267.4867389	273.3987754	320.9286308	Genetic Informat	Transcription	RNA polymerase

ko03022	0.609287419	0.513361531	0.55873694	0.18069166	0.27950889	0.632353956	0.009208542	0.445557917	0.01754386	0.014406665	0.266581564	0.124005313	0.04975067	Genetic Informat	Transcription	Basal transcription factors
ko03030	138.7508929	141.5017917	123.7679141	60.41906292	63.74479207	78.25187153	202.0820098	176.0403026	175.23643	143.823417	127.0243793	137.0444213	208.9438787	Genetic Informat	Replication and repair	DNA replication
ko03040	4.106328426	3.444086405	4.081448426	4.408038824	6.169825503	13.15345885	0.860516497	0.09924608	0.066066535	1.12218401	2.678054946	1.490404186	0.08714908	Genetic Informat	Transcription	Spliceosome
ko03050	4.502770649	4.849865015	4.888267983	0.4069224	0.687714365	1.14285245	54.17680535	43.18631159	41.35363594	6.390055359	6.101899809	9.083355868	53.20046524	Genetic Informat	Folding, sorting and degradation	Proteasome
ko03060	55.68258281	59.82580049	53.17459956	33.4334515	31.92193512	37.44819234	51.5761004	50.4418381	45.89896878	47.21535421	42.44489032	45.28645445	56.81664658	Genetic Informat	Folding, sorting and degradation	Protein export
ko03070	5.429634695	7.3710467	24.11979182	81.30777942	159.3961071	114.2002025	24.45813178	11.82893985	15.56521348	12.79871747	12.0545991	9.61474941	20.72127352	Environmental I	Membrane transport	Bacterial secretion system
ko03320	0.049295228	0.157116253	2.032643204	2.1115227	1.235708261	4.215089512	0.390060381	0.236224464	0.022644109	0.9891395	0.596360756	0.615496671	0.284564545	Organismal Syst	Endocrine system	PPAR signaling pathway
ko03410	173.4062758	181.6483785	164.956476	86.66866765	87.73200697	91.49197363	204.5961284	197.1690312	186.5235807	172.1165811	152.4075653	154.5223685	207.8677314	Genetic Informat	Replication and repair	Base excision repair
ko03420	299.0947382	321.3324576	263.5757344	131.2203355	132.8929688	142.4417347	181.2781113	149.4360115	164.9318653	210.2357966	193.331044	190.383275	202.0908707	Genetic Informat	Replication and repair	Nucleotide excision repair
ko03430	199.2579167	210.0020934	164.4007914	111.8335676	101.0515676	113.3339082	112.1323971	91.14538673	92.93267615	112.8054645	101.7432526	102.2080149	117.0802342	Genetic Informat	Replication and repair	Mismatch repair
ko03440	415.090791	428.7680865	376.5413552	177.1968378	181.7448967	197.7335661	189.5743588	172.7836368	176.1836879	258.4122448	237.2447909	228.450928	236.4750478	Genetic Informat	Replication and repair	Homologous recombination
ko03450	0.123186256	0.538799375	0.375688828	0.447015597	0.387250127	0.527904429	10.42176122	10.8070319	8.865366764	1.549315258	1.878816671	1.445488498	14.65724208	Genetic Informat	Replication and repair	Non-homologous end-joining
ko03460	0.243009217	0.236628892	0.265798642	1.801999559	0.802080072	2.546641293	0.100029791	0.087570621	0.119286511	0.838842907	0.944129107	1.179588741	0.028163675	Genetic Informat	Replication and repair	Fanconi anemia pathway
ko04010	0.094870778	0.076674702	0.132728302	0.116955729	0.225805235	0.396211443	0.977659352	0.077319979	0.034825624	0.059648713	0.216841814	0.140805251	0.001145475	Environmental I	Signal transduction	MAPK signaling pathway
ko04011	0.045145954	0.045740235	0.064298587	0.068982545	0.114413546	0.189244666	0.002411091	0	0	0.087425289	0.102832131	0.549325688	0	Environmental I	Signal transduction	MAPK signaling pathway - yeast
ko04013	0.124362804	0.14143673	0.13678653	0.255045542	0.322011427	0.662262402	0.019607843	0	0	0	0.043831169	0.022222222	0.004403563	Environmental I	Signal transduction	MAPK signaling pathway - fly
ko04014	0.794485415	0.947743405	0.912181145	0.560389207	0.686559126	0.585582485	0	0.044905009	0.085534591	0.025989365	0.072685329	0.072926608	0	Environmental I	Signal transduction	Ras signaling pathway
ko04016	0.096796856	0.118915701	0.12967219	0.855998405	0.384385761	0.479862132	0.026565465	0.133459836	0.054515368	0.300417738	0.41940349	0.28247125	0	Environmental I	Signal transduction	MAPK signaling pathway - plant
ko04020	0	0.001013171	0.001013171	0	0	0	0	0	0	0.006079027	0.041033435	0.011651469	0	Environmental I	Signal transduction	Calcium signaling pathway
ko04068	0.00191022	0	0.00191022	0.022222222	0.06962963	0.100740741	0	0	0	0.00191022	0.038204394	0.017191977	0	Environmental I	Signal transduction	FoxO signaling pathway
ko04070	0.251390897	0.113581013	0.238729883	0.101757453	0.274926299	0.461858524	0	0	0	0	0.024586288	0.009463911	0.001801802	Environmental I	Signal transduction	Phosphatidylinositol signaling system
ko04071	0.067993794	0.057475001	0.077391097	0.0720798	0.121052058	0.237830754	0	0	0.04954955	0.020366836	0.116291634	0.035451189	0	Environmental I	Signal transduction	Sphingolipid signaling pathway
ko04075	0.032192624	0.035426731	0.06289807	0.331511438	1.061160837	0.953350581	0	0.07962963	0	0.035631367	0.040606491	0.023004027	0.011973775	Environmental I	Signal transduction	Plant hormone signal transduction
ko04080	0	0	0	0.012300123	0.056580566	0.070110701	0	0	0	0	0	0	0	Environmental I	Signaling molecules and interactio	Neuroactive ligand-receptor interaction
ko04111	0.663012883	0.650505215	0.716993506	0.489409395	1.103024577	1.622246695	0	0	0.183450935	0.107187938	0.630419508	0.178722115	0.005774502	Cellular Process	Cell growth and death	Cell cycle - yeast
ko04112	325.5803207	345.41955	308.10619	177.6934624	178.1172206	189.4846516	199.0269871	211.3937439	206.8324794	277.8218891	253.6033378	253.5258996	243.736178	Cellular Process	Cell growth and death	Cell cycle - Caulobacter
ko04113	4.427615432	3.730997915	2.207857186	4.187616527	7.500709118	4.987473002	0.216591925	0.133809284	0.070222309	0.514373267	0.516523503	0.309564559	0.363033334	Cellular Process	Cell growth and death	Meiosis - yeast
ko04114	0	0	0	0.021419219	0.086279499	0.170341934	0	0	0	0.015151515	0.022727273	0.003787879	0	Cellular Process	Cell growth and death	Oocyte meiosis
ko04115	0.515208803	0.379506397	0.388733192	0.357784003	0.318794705	0.614217372	0.234765438	0.164084159	0.009920703	0.164832599	0.768822704	0.355032167	0.198323109	Cellular Process	Cell growth and death	p53 signaling pathway
ko04120	1.033446573	0.815333001	1.1254084	6.821667306	4.126822793	8.649633399	2.603609351	5.088135593	5.383448896	4.633828343	4.481528609	3.703245123	0.227837132	Genetic Informat	Folding, sorting and degradation	Ubiquitin mediated proteolysis
ko04122	105.7961897	116.1252457	101.6685326	46.54143185	53.41826621	57.48121162	108.2969959	96.48580067	101.3268722	79.36118283	70.5841934	75.35011828	135.2462734	Genetic Informat	Folding, sorting and degradation	Sulfur relay system
ko04130	0.323098561	0.228632887	0.263011442	0.704210184	0.452575206	0.820424883	0	0	0	0.002936858	0.002911208	0.005031447	0.006396696	Genetic Informat	Folding, sorting and degradation	SNARE interactions in vesicular transport
ko04137	0.04657083	0.044174903	0.031184486	0.147885906	0.362433414	0.742825291	0.080764436	0	0	0.00245098	0.031862745	0.024509804	0.00621202	Cellular Process	Transport and catabolism	Mitophagy - animal
ko04138	17.06477426	17.30539523	21.71940316	9.394872943	5.839046086	7.914072221	12.55573721	14.70915959	12.72259296	23.78283112	18.31339637	17.77347357	18.41547964	Cellular Process	Transport and catabolism	Autophagy - yeast
ko04139	0.349410177	0.313869986	0.373619995	0.206806531	0.299414303	0.564897878	0.050989976	0.005291005	0.190192244	0	0	0	0.047104896	Cellular Process	Transport and catabolism	Mitophagy - yeast
ko04140	0.068952391	0.033509169	0.085038992	0.261436241	0.29768454	2.071930238	0.291710776	0.030120482	0	0.139122381	0.093902468	0.150812708	0.000518403	Cellular Process	Transport and catabolism	Autophagy - animal
ko04141	5.04314538	4.894671226	7.100161663	19.07507604	12.43585755	23.39614217	60.03786376	59.41620072	52.10427156	14.25682303	12.47397535	13.37842289	41.08013565	Genetic Informat	Folding, sorting and degradation	Protein processing in endoplasmic reticulum
ko04142	8.797856632	9.14235509	9.498310311	16.88072597	13.45589404	28.12168177	9.712590393	7.453679162	6.752040664	9.736741757	9.588208554	7.955510213	10.52377862	Cellular Process	Transport and catabolism	Lysosome
ko04144	1.371004592	1.14324069	1.272340702	1.537339504	2.715309496	5.061331124	0.431795772	0.095639654	0.101794563	0.516000788	1.703177081	0.788913766	1.571275883	Cellular Process	Transport and catabolism	Endocytosis
ko04146	13.73335941	15.12432488	9.266010132	1.886285882	1.279073725	1.88747176	8.88752661	9.71170108	8.046538893	7.046759267	7.338559201	5.708138029	6.790323273	Cellular Process	Transport and catabolism	Peroxisome
ko04150	0.16326003	0.107015479	0.081992461	0.193026004	0.544674152	0.787975591	0	0	0	0.016682767	0.214765754	0.099252717	0.0029449	Environmental I	Signal transduction	mTOR signaling pathway
ko04151	0.060945134	0.050874641	0.061533999	0.034178029	0.052311436	0.109489051	0	0	0	0.03071636	0.154287614	0.034353612	0	Environmental I	Signal transduction	PI3K-Akt signaling pathway
ko04152	0.091755345	0.078720148	0.073495195	0.575635858	0.24663028	0.544269384	0	0	0	0.115505278	0.09253301	0.111757082	0.00933924	Environmental I	Signal transduction	AMPK signaling pathway
ko04210	1.965770488	2.256531244	1.776697322	3.086437469	3.472410427	5.417915964	5.804307615	12.2533388	3.336775931	2.74364241	3.36639713	2.676087572	3.161215437	Cellular Process	Cell growth and death	Apoptosis
ko04212	0.091313657	0.060064205	0.081614292	0.03664147	0.050304688	0.157372854	0	0	0	0.01210121	0.051743859	0.029314344	0.007751938	Organismal Syst	Aging	Longevity regulating pathway - worm
ko04213	64.47420362	71.73304494	54.22556135	28.69774837	32.63903376	34.0870435	29.83153196	25.09468524	27.27041371	29.71844346	25.88250385	26.67314485	38.15856777	Organismal Syst	Aging	Longevity regulating pathway - multiple species
ko04214	27.46417804	28.64353346	19.10798347	13.52111725	13.24398136	17.35496123	5.960950051	2.882157321	2.951358872	15.06068791	13.50602954	15.1229228	5.985653907	Cellular Process	Cell growth and death	Apoptosis - fly
ko04216	0	0	0	0	0	0.016528926	0.018666667	0.002666667	0.002666667	0.085399449	0.035812672	0.016528926	0	Cellular Process	Cell growth and death	Ferroptosis
ko04217	0.028490028	0.008547009	0.020892688	0.481745966	0.073047696	0.179081072	0	0	0	0.208127155	0.128280102	0.109211776	0.046061048	Cellular Process	Cell growth and death	Necroptosis
ko04218	0.327148179	0.277672225	0.338653299	0.207461049	0.241715638	0.381457559	0	0	0	0.005638025	0.08647067	0.024923206	0.003663004	Cellular Process	Cell growth and death	Cellular senescence
ko04261	0	0	0	0	0	0	0	0	0	0.025488249	0.100007986	0.030845352	0	Organismal Syst	Circulatory system	Adrenergic signaling in cardiomyocytes
ko04310	0.222133021	0.150121179	0.252691838	0.34849606	0.236367348	0.457514083	0.070827714	0.137977211	0.246070245	0.497304589	1.854005573	1.245095514	0.125089739	Environmental I	Signal transduction	Wnt signaling pathway
ko04320	0	0	0	0.179812747	0.018738235	0.050938338	0	0	0	0.013899747	0.190083849	0.022651854	0	Organismal Syst	Development	Dorso-ventral axis formation
ko04330	0.052726409	0.021352828	0.039871346	0.135964943	0.079304123	0.199021157	0	0.006319115	0	1.504432194	1.353090948	0.691952142	0	Environmental I	Signal transduction	Notch signaling pathway
ko04340	0.043709783	0.042210137	0.082895779	0.094396759	0.121910727	0.255485205	0	0	0	0.00392279	0.15563908	0.075811983	0	Environmental I	Signal transduction	Hedgehog signaling pathway
ko04341	0	0	0	0	0	0	0	0	0.045698925	0	0	0	0	Environmental I	Signal transduction	Hedgehog signaling pathway - fly
ko04391	0.174046065	0.113746517	0.12048745	0.059950435	0.229306587	0.288072741	0	0.002710027	0	0	0	0.005859921	0	Environmental I	Signal transduction	Hippo signaling pathway -fly
ko04512	0	0	0	0.266201182	0.224884343	0.321415822	0.262042389	0	0	0	0	0	0	Environmental I	Signaling molecules and interactio	ECM-receptor interaction
ko04514	0.063085554	0.063930185	0.08153247	0.107234866	0.053311257	0.154152717	0.052410901	0	0	0.661687537	0.434187425	0.355783846	0	Environmental I	Signaling molecules and interactio	Cell adhesion molecules (CAMs)
ko04520	0	0	0	0	0	0	0.028735632	0	0.009578544	0	0	0	0	Cellular Process	Cellular community - eukaryotes	Adherens junction
ko04530	0	0	0	0.136938646	0.05700506	0.097564832	0	0	0	0	0	0	0	Cellular Process	Cellular community - eukaryotes	Tight junction
ko04550	0.051344005	0.090098661	0.048142555	0.062195484	0.087398672	0.205387382	0	0	0	0	0	0	0	Cellular Process	Cellular community - eukaryotes	Signaling pathways regulating pluripotency of stem cells
ko04610	0.034608379	0.024635701	0.036429872	0	0	0	0.008097612	0.54378646	0.034141399	0.027176527	0.083509234	0.035682126	0.049162257	Organismal Syst	Immune system	Complement and coagulation cascades
ko04612	0	0	0	0.044160964	0.059608442	0.168925653	0	0	0	0	0.034722222	0.025462963	0	Organismal Syst	Immune system	Antigen processing and presentation
ko04614	17.64436832	15.90939169	11.81350128	10.58377998	7.890816555	12.54263174	7.00854268	5.739908324	4.102127295	5.419464142	6.639994384	5.875937092	4.881908225	Organismal Syst	Endocrine system	Renin-angiotensin system
ko04621	0.02504817	0.01734104	0.021194605	0.048816908	0.158562925	0.436996667	0.040650407	0	0	0	0.034482759	0.01532567	0	Organismal Syst	Immune system	NOD-like receptor signaling pathway
ko04622	0.088706343	0.111103457	0.140026292	0.04883914	0.050186538	0.640369618	0.059379395	0.480794553	0.012027491	0.268659246	0.593352515	0.407345925	0	Organismal Syst	Immune system	RIG-I-like receptor signaling pathway
ko04624	0.079254118	0.057002096	0.066979886	0.017898374	0.041577364	0.027204331	0.091849833	0.039007092	0.042860657	0.122050017	0.083107683	0.062375753	0.034665539	Organismal Syst	Immune system	Toll and Imd signaling pathway
ko04626	101.7719303	100.8134632	77.49239678	61.40465767	77.52372768	56.64801558	59.40252087	60.20959214	57.45951788	59.63033631	55.83197607	56.87429897	64.83477833	Organismal Syst	Environmental adaptation	Plant-pathogen interaction
ko04657	0	0	0	0.031463024	0.120677987	0.148541897	0	0	0	0	0	0	0	Organismal Syst	Immune system	IL-17 signaling pathway
ko04660	0	0	0	0	0	0	0	0	0	0.005524862	0.020257827	0.005524862	0	Organismal Syst	Immune system	T cell receptor signaling pathway
ko04662	0	0	0.006006006	0	0	0	0	0	0	0.006006006	0.052552553	0.010510511	0	Organismal Syst	Immune system	B cell receptor signaling pathway
ko04710	0.181862188	0.132490408	0.124354227	0.165408617	0.103989764	0.194821183	0	0	0	0.120282024	0.15752112	0.227520189	0.001053186	Organismal Syst	Environmental adaptation	Circadian rhythm
ko04711	0.030448718	0.020833333	0.044871795	0	0	0	0	0	0	0	0	0.003205128	0	Organismal Syst	Environmental adaptation	Circadian rhythm - fly
ko04712	0.204672423	0.146703719	0.200072887	0	0	0	0	0	0	0	0	0.005914644	0	Organismal Syst	Environmental adaptation	Circadian rhythm - plant
ko04714	12.27772817	11.71989883	10.60815169	9.322217954	6.491179969	8.677212799	9.949136286	19.5265635	13.01179055	12.92828099	12.13816416	10.57506322	9.445126466	Organismal Syst	Environmental adaptation	Thermogenesis
ko04721	0.082303114	0.056551731	0.032601029	0.031429393	0.114664941	0.209651639	0	0	0	0.029766064	0.201480445	0.111505352	0.001754386	Organismal Syst	Nervous system	Synaptic vesicle cycle
ko04722	0	0	0	0.344104308	0.178362218	0.218051506	0	0	0	0.009259259	0.037037037	0	0.00462963	Organismal Syst	Nervous system	Neurotrophin signaling pathway
ko04723	4.472374796	4.292159756	7.506108227	1.660913228	2.422147144	1.634421287	1.885100449	4.6259124	3.294573368	5.713788154	5.162010202	5.560247346	3.115942136	Organismal Syst	Nervous system	Retrograde endocannabinoid signaling
ko04724	0	0	0	0	0	0	0	0	0	0.003546099	0.058510638	0.012411348	0	Organismal Syst	Nervous system	Glutamatergic synapse
ko04728	0	0	0	0.01659751	0.042876902	0.065006916	0	0	0	0	0	0	0	Organismal Syst	Nervous system	Dopaminergic synapse
ko04740	0.052434457	0.014939659	0.028089888	0	0	0	0	0	0	0.011742595	0.084224535	0.029443747	0	Organismal Syst	Sensory system	Olfactory transduction

ko04742	0	0	0.004246285	0	0	0	0	0	0.021978022	0.004433153	0.06305812	0.033902685	0	Organismal Syst	Sensory system	Taste transduction
ko04744	0	0	0	0.070009225	0.181949106	0.347636771	0	0	0	0	0	0	0.00254104	Organismal Syst	Sensory system	Phototransduction
ko04745	0.084424726	0.128316259	0.123837809	0.066044362	0.070535733	0.144699682	0	0	0	0.004206099	0.004206099	0	0.001232286	Organismal Syst	Sensory system	Phototransduction - fly
ko04750	0.008915573	0.006092833	0.015833298	0.269106878	0.379154435	0.478354975	0.392744568	0.044290444	0.686057004	1.004697053	1.217743322	0.542614809	0.000248571	Organismal Syst	Sensory system	Inflammatory mediator regulation of TRP channels
ko04810	0	0	0	0.014652015	0.031135531	0.054945055	0	0	0	0.011188792	0.057736075	0.035140439	0.003663004	Cellular Process	Cell motility	Regulation of actin cytoskeleton
ko04910	1.357647886	1.402721908	1.459315056	5.859172778	2.748674361	5.600493017	7.173516438	3.550212612	3.139023213	0.933644114	2.608671041	1.711137933	2.688335619	Organismal Syst	Endocrine system	Insulin signaling pathway
ko04914	0	0	0	0.080920512	0.05988024	0.100798403	0	0	0	0	0	0	0	Organismal Syst	Endocrine system	Progesterone-mediated oocyte maturation
ko04915	0.106262299	0.080158278	0.029927761	0	0.00273224	0.032786885	0	0	0	0.030422925	0.110424659	0.035236098	0	Organismal Syst	Endocrine system	Estrogen signaling pathway
ko04918	8.677656881	7.728789586	5.252208578	2.993602724	2.723920486	2.259794772	4.091922392	4.222074123	2.669901935	5.168864481	5.083849393	6.021805063	4.013771121	Organismal Syst	Endocrine system	Thyroid hormone synthesis
ko04919	0.082514913	0.068033125	0.061874566	0.101845257	0.155697297	0.326207699	0.649722861	0.579440731	0.675260277	0.021072797	0.048432357	0.065808645	0.101722035	Organismal Syst	Endocrine system	Thyroid hormone signaling pathway
ko04922	0.015053763	0.034408602	0.017204301	0.065762026	0.102160494	0.092901235	0	0	0	0.008948546	0.0758664	0.031570338	0	Organismal Syst	Endocrine system	Glucagon signaling pathway
ko04923	0.003787879	0.026515152	0.003787879	0.029239766	0.051169591	0.057017544	0	0	0	0.008573047	0.050676665	0.01183432	0	Organismal Syst	Endocrine system	Regulation of lipolysis in adipocyte
ko04928	0.379700459	0.336114883	0.351291771	0.059813928	0.034700173	0.080145102	7.859710526	7.804683857	7.166650585	0.348079802	0.476133505	0.850669116	8.356382022	Organismal Syst	Endocrine system	Parathyroid hormone synthesis, secretion and action
ko04931	0.163939704	0.103371387	0.13332675	0.041228423	0.066325651	0.11690327	0	0	0	0.042393331	0.179362795	0.097109286	0	Human Diseases	Endocrine and metabolic diseases	Insulin resistance
ko04934	0.134428757	0.09976069	0.164043555	0.027124774	0.12682166	0.181386686	0.110014077	0	0.01327058	0.010430283	0.179515383	0.04307474	0.141040692	Human Diseases	Endocrine and metabolic diseases	Cushing's syndrome
ko04940	0	0.003898635	0.001949318	0	0	0	0	0	0	0.045892509	0.029239766	0.031189084	0	Human Diseases	Endocrine and metabolic diseases	Type I diabetes mellitus
ko04964	0	0	0	0.022292994	0.065293895	0.09862432	0	0	0	0.003546099	0.042553191	0.014184397	0	Organismal Syst	Excretory system	Proximal tubule bicarbonate reclamation
ko04966	0.138095908	0.172045886	0.151113723	0.025700935	0.063862928	0.087227414	0.034547152	0	0	0	0	0.00564924	0	Organismal Syst	Excretory system	Collecting duct acid secretion
ko04970	0.004046471	0.008666374	0.025752986	0.215507237	0.148094039	0.263286846	0.094827586	0	0.017241379	0.253522151	0.243235602	0.248343471	0	Organismal Syst	Digestive system	Salivary secretion
ko04972	0	0	0	0.005464481	0	0.016393443	0	0	0	0.00273224	0	0.00273224	0	Organismal Syst	Digestive system	Pancreatic secretion
ko04973	0	0	0	0.038900871	0.04957265	0.090598291	0	0	0	0	0	0	0	Organismal Syst	Digestive system	Carbohydrate digestion and absorption
ko04974	13.44517081	12.17768859	8.252827789	16.95196667	9.273677814	16.64792224	5.195574678	5.297947939	4.191825311	7.246651713	6.01813738	4.739285903	4.901333078	Organismal Syst	Digestive system	Protein digestion and absorption
ko04976	0.012280702	0.024561404	0.043859649	0.033941356	0.150556411	0.217404628	0	0	0	0.033333333	0.029233799	0.075689223	0	Organismal Syst	Digestive system	Bile secretion
ko04977	0.409385125	0.404389632	0.472817849	0.414217476	0.717665773	2.428060749	0.549921957	0.582797055	0.567284866	0.381534234	0.76375152	0.421531669	1.563993794	Organismal Syst	Digestive system	Vitamin digestion and absorption
ko04978	2.327321535	2.803202037	1.129738844	3.696537359	2.723571945	4.85337348	2.135127205	2.423378457	2.435434497	2.942610222	2.070423741	2.88078006	1.917372728	Organismal Syst	Digestive system	Mineral absorption
ko05010	20.46239185	23.44674263	26.07270463	6.566281126	13.51245671	7.914235193	2.208859252	0.879542376	3.154418355	2.988188796	3.266095414	5.309456915	0.749791777	Human Diseases	Neurodegenerative diseases	Alzheimer's disease
ko05012	0	0.000797448	0	0.072298691	0.121983628	0.245917712	0.04855643	0.014435696	0.002624672	0.022469763	0.125207669	0.089040072	0	Human Diseases	Neurodegenerative diseases	Parkinson's disease
ko05014	0.017777778	0.019259259	0.020740741	0.069885686	0.042382589	0.095647194	0	0	0	0	0	0	0	Human Diseases	Neurodegenerative diseases	Amyotrophic lateral sclerosis (ALS)
ko05016	5.291586221	5.311503061	6.160001479	4.309313196	7.516777667	15.65448785	0.003756137	0.04141575	0.028207781	0.883458509	6.503471529	2.735775919	0.098692465	Human Diseases	Neurodegenerative diseases	Huntington's disease
ko05020	0.274009514	0.255810134	0.285860749	0.328812508	0.940250007	1.207959134	0	0.030828516	0.026974952	0.065131178	0.224298422	0.11367521	0.09137412	Human Diseases	Neurodegenerative diseases	Prion diseases
ko05034	0.092479613	0.085887235	0.101166612	0.628802512	0.374828599	0.818482219	0	0	0	0.005362145	0.041176471	0.001960784	0.003902116	Human Diseases	Substance dependence	Alcoholism
ko05100	0.148823924	0.113738259	0.204193829	1.681872222	2.118426144	1.624847758	0.781677004	1.442643511	0.56767454	0.258190013	0.310339027	0.180992476	0.412661865	Human Diseases	Infectious diseases	Bacterial invasion of epithelial cells
ko05110	0.266079275	0.251061716	0.227551292	0.14117135	0.147107801	0.300056228	0.190545017	0.004938272	0	0.281261897	1.598230863	0.168069047	0.010082201	Human Diseases	Infectious diseases	Vibrio cholerae infection
ko05111	47.36119693	54.13812935	58.87935643	58.46086215	63.5601717	75.3967837	40.80981061	33.42887201	33.54435187	26.94482997	21.93363178	21.26768166	40.9750785	Cellular Process	Cellular community - prokaryotes	Biofilm formation - Vibrio cholerae
ko05120	14.09269249	17.36917795	9.56052479	15.20658719	11.71456868	18.69021894	1.81615205	3.533680187	3.567409222	5.851022554	6.42163918	8.563606794	1.213051959	Human Diseases	Infectious diseases	Epithelial cell signaling in Helicobacter pylori infection
ko05130	1.04750163	0.878122394	1.039285349	2.946043232	3.75047794	6.430729994	0.427009198	0.005834508	0	0.434266202	1.131093823	0.711453582	0.041762396	Human Diseases	Infectious diseases	Pathogenic Escherichia coli infection
ko05131	0	0	0	0.01754386	0.047908232	0.098178138	0	0	0	0	0.019267823	0.003853565	0.02688172	Human Diseases	Infectious diseases	Shigellosis
ko05132	0.232566941	0.222930766	0.276988818	0.098182317	0.274523321	0.527757802	0.204212965	0.481925645	1.176138134	0.090012876	0.275953965	0.100140154	0.320554217	Human Diseases	Infectious diseases	Salmonella infection
ko05133	1.841228956	1.928149189	1.51057531	0.946286955	2.094787299	2.946436632	1.881546683	1.907715026	1.251062557	1.818137412	1.734958961	1.538733635	1.336815086	Human Diseases	Infectious diseases	Pertussis
ko05134	54.28936709	59.35193814	49.3107629	24.05432646	29.48010207	27.04185063	26.21771169	25.3606726	26.8171258	49.47314816	44.5150667	44.43221585	32.95962676	Human Diseases	Infectious diseases	Legionellosis
ko05143	0.026844626	0.028987397	0.006837607	0.201612835	0.322318134	0.144727135	1.263022626	0.313910047	0.997315692	0.873561866	0.673507697	0.383896248	1.482526394	Human Diseases	Infectious diseases	African trypanosomiasis
ko05146	0	0	0.001763668	0.219000654	0.545079036	0.501175352	0.351799753	0.567610607	0.1169364	0.003518054	0.009786514	0	0.238443894	Human Diseases	Infectious diseases	Amoebiasis
ko05150	0.319594286	0.389963149	0.399103305	1.506857693	1.849889293	1.120472966	1.483614268	1.084186899	0.810560996	0.328365837	0.338624022	0.30064942	3.861619169	Human Diseases	Infectious diseases	Staphylococcus aureus infection
ko05152	0.633996705	0.86467412	0.886091368	0.120449444	0.120249241	0.233417328	4.147951191	0.831240564	0.635731491	2.467053299	1.731807015	1.611704378	2.069171649	Human Diseases	Infectious diseases	Tuberculosis
ko05162	0.055550429	0.115868268	0.051142138	0.048026048	0	0.012922263	0.051014189	0.046696524	0.011076643	0.013882579	0.077402616	0.02442906	0.04	Human Diseases	Infectious diseases	Measles
ko05164	0.079108778	0.045224864	0.085391582	0.886750796	0.239215286	0.68002396	0	0	0	0.024658217	0.156629499	0.126193617	0.002829037	Human Diseases	Infectious diseases	Influenza A
ko05165	0.048651507	0.04981932	0.069165345	0.003663004	0.054945055	0.095238095	0	0	0	0	0	0	0	Human Diseases	Infectious diseases	Human papillomavirus infection
ko05166	0.453473248	0.417909301	0.535961157	0.277142942	0.632438299	1.136197407	0	0	0	0.032244404	0.174698409	0.073000154	0.003030303	Human Diseases	Infectious diseases	HTLV-I infection
ko05168	0.002339181	0	0	0.181052757	0.140116959	1.113024987	0	0	0	0.022063047	0.003944773	0	0	Human Diseases	Infectious diseases	Herpes simplex infection
ko05170	0.392248511	0.503218581	3.049008923	2.505251419	0.882842751	3.345624355	0.171822129	0.001363327	0	1.120391376	1.059427943	1.196820852	0.091801916	Human Diseases	Infectious diseases	Human immunodeficiency virus 1 infection
ko05202	0.090042964	0.117372613	0.091995917	0.636172203	0.10311198	0.271804791	0.015151515	0	0	0.02894373	0.20198947	0.09201927	0	Human Diseases	Cancers	Transcriptional misregulation in cancers
ko05203	0.015122873	0.020163831	0.047860937	0.028039391	0.161430158	0.249386753	0	0	0	0.020290219	0.207687388	0.063694586	0	Human Diseases	Cancers	Viral carcinogenesis
ko05204	0.293310152	0.343563447	0.303025234	1.75459668	1.724710332	0.961643559	2.50509887	4.92276682	3.108357116	0.716694155	0.576266286	0.351797284	3.818929333	Human Diseases	Cancers	Chemical carcinogenesis
ko05206	0	0	0	0.017897092	0.044742729	0.055928412	0	0	0	0.008210181	0.013136289	0.034482759	0	Human Diseases	Cancers	MicroRNAs in cancer
ko05225	0.181160914	0.177067224	0.1933608	0.031899464	0.096472825	0.240649222	0	0	0	0.014294716	0.12760055	0.042134916	0.001872659	Human Diseases	Cancers	Hepatocellular carcinoma
ko05226	0	0	0	0	0	0	0	0	0	0.007130125	0.035650624	0	0	Human Diseases	Cancers	Gastric cancer
ko05230	0.034227726	0.033122246	0.037065703	0.004901961	0	0	0.578690834	0.372534912	0.598856238	0.150756794	0.137903384	0.106381149	0.433125059	Human Diseases	Cancers	Central carbon metabolism in cancer
ko05231	0.391661014	0.295442494	0.385102815	0.041125541	0.04923415	0.128040121	0	0	0	0.029660132	0.284992702	0.099361369	0	Human Diseases	Cancers	Choline metabolism in cancer
ko05322	0.012944984	0.009708738	0.012269033	0.06958466	0.140986781	0.165215893	4.345337171	0.396594873	1.03274935	0.027061374	0.176460213	0.107408022	0.851370366	Human Diseases	Immune diseases	Systemic lupus erythematosus
ko05412	0	0	0	0	0	0	0.04342723	0	0.007042254	0	0	0	0	Human Diseases	Cardiovascular diseases	Arrhythmogenic right ventricular cardiomyopathy (ARVC)
