# Supplementary material for: Fate and carbon sequestration potential of sunken macroalgae in coastal oceans from long-term microbial degradation perspective
Source: Natl Sci Rev. 2025 Jul 8;12(8):nwaf273. doi: 10.1093/nsr/nwaf273 (PMC12365756; doi:10.1093/nsr/nwaf273)
Supplement: nwaf273_Supplemental_Files [file nwaf273_supplemental_files.zip › Table__S3.pdf]

Table S3 Different modules generated by WGCNA and their corresponding functional profiles

Modules	KEGG_KO
Module 01	K00078;K00224;K00280;K00387;K00503;K00516;K00591;K00594;K00629;K00639;K00649;K00653;K00667;K00668;K00744;K00753;K00774;K00905;K00920;K00933;K00976;K00988;K01098;K01189;K01204;K01205;K01217;K01225;K01231;K01276;K01291;K01314;K01320;K01369;K01379;K01415;K01429;K01466;K01498;K01526;K01753;K01850;K02156;K02328;K02555;K02977;K03190;K03286;K03347;K03357;K03360;K03361;K03507;K03535;K03691;K03835;K03858;K03927;K04364;K04424;K04427;K04445;K04456;K04494;K04506;K04512;K04521;K04560;K04602;K04606;K04678;K04782;K04834;K04837;K04838;K04840;K04842;K04843;K04849;K04853;K04854;K04855;K04857;K04889;K04905;K04908;K04911;K04913;K04915;K04949;K04950;K04951;K04952;K04955;K04977;K04990;K05011;K05038;K05323;K05391;K05399;K05624;K05633;K05659;K05666;K05669;K05670;K05688;K05745;K05769;K05841;K05849;K05850;K05869;K05906;K05986;K06052;K06129;K06252;K06271;K06277;K06446;K06496;K06545;K06560;K06665;K06671;K06692;K06777;K06826;K06835;K06896;K07298;K07366;K07377;K07378;K07756;K07759;K07880;K07964;K08008;K08214;K08291;K08469;K08574;K08578;K08581;K08585;K08657;K08781;K08806;K08853;K08985;K09420;K09520;K09530;K09538;K09564;K09566;K09576;K09578;K09586;K09602;K09630;K09654;K09668;K09675;K09842;K09956;K10060;K10151;K10190;K10223;K10224;K10262;K10273;K10352;K10355;K10361;K10370;K10415;K10423;K10442;K10454;K10463;K10464;K10520;K10567;K10591;K10594;K10608;K10625;K10641;K10646;K10684;K10688;K10689;K10693;K10776;K10895;K10910;K10954;K11000;K11049;K11087;K11097;K11140;K11169;K11187;K11224;K11270;K11436;K11485;K11585;K11587;K11604;K11605;K11606;K11607;K11655;K11664;K11715;K11728;K11733;K11735;K11860;K11862;K11866;K11873;K11876;K11972;K11977;K12042;K12056;K12057;K12058;K12059;K12060;K12061;K12063;K12064;K12065;K12067;K12068;K12072;K12164;K12165;K12171;K12229;K12231;K12232;K12233;K12302;K12309;K12310;K12311;K12312;K12314;K12375;K12376;K12381;K12392;K12430;K12446;K12488;K12660;K12667;K12670;K12824;K12894;K12943;K13039;K13076;K13131;K13146;K13148;K13184;K13202;K13249;K13291;K13335;K13341;K13352;K13447;K13448;K13486;K13510;K13538;K13660;K13667;K13702;K13720;K13738;K13750;K13751;K13755;K13761;K13763;K13887;K13890;K13912;K13937;K13982;K13987;K14008;K14258;K14423;K14429;K14453;K14498;K14510;K14557;K14607;K14616;K14621;K14693;K14725;K14735;K14736;K14785;K14800;K14959;K15009;K15074;K15117;K15148;K15188;K15202;K15228;K15229;K15259;K15284;K15289;K15290;K15293;K15300;K15303;K15381;K15397;K15401;K15410;K15447;K15490;K15499;K15562;K15621;K15628;K15653;K15664;K15666;K15688;K15698;K15717;K15732;K15893;K15909;K15920;K16080;K16195;K16197;K16250;K16280;K16286;K16296;K16330;K16416;K16417;K16462;K16510;K16573;K16586;K16604;K16607;K16681;K16729;K16751;K16754;K16811;K16813;K16892;K16893;K17085;K17094;K17098;K17099;K17108;K17236;K17253;K17275;K17307;K17338;K17342;K17458;K17495;K17593;K17923;K17928;K17970;K18040;K18042;K18058;K18079;K18097;K18247;K18261;K18320;K18438;K18577;K18640;K18667;K18669;K18696;K18730;K18735;K18748;K18801;K18826;K18933;K18966;K18980;K19007;K19160;K19161;K19312;K19357;K19365;K19370;K19372;K19418;K19477;K19480;K19496;K19497;K19499;K19500;K19501;K19502;K19527;K19538;K19601;K19662;K19704;K19749;K19776;K19782;K19881;K19882;K19906;K19908;K19919;K19932;K19947;K19970;K19977;K20045;K20046;K20091;K20092;K20111;K20170;K20174;K20175;K20176;K20247;K20279;K20290;K20365;K20369;K20488;K20520;K20522;K20526;K20538;K20551;K20656;K20712;K20754;K20844;K20972;K21289;K21511;K21635;K21682;K21773;K21804;K21841;K21862;K21863;K21914;K21918;K21954;K21971;K22030;K22127;K22207;K22217;K22244;K22277;K22372;K22374;K22378;K22380;K22614;K22733;K22745;K22816;K22827;K23009;K23012;K23079;K23093;K23344;K23387;K23451;K23470;K23486;K23531;K23545;K23563;K23593;K23624;K23664;K23737;K23882;K23887;K23894;K23953;K24030;K24042

Module 02	K00004;K00006;K00011;K00026;K00041;K00046;K00065;K00079;K00102;K00103;K00106;K00139;K00143;K00162;K00225;K00234;K00235;K00236;K00242;K00261;K00314;K00323;K00326;K00327;K00339;K00357;K00362;K00367;K00372;K00380;K00423;K00425;K00426;K00428;K00433;K00434;K00455;K00456;K00479;K00486;K00500;K00508;K00521;K00522;K00529;K00550;K00556;K00574;K00592;K00597;K00599;K00606;K00654;K00656;K00657;K00665;K00670;K00671;K00682;K00685;K00694;K00698;K00703;K00715;K00726;K00729;K00731;K00787;K00796;K00804;K00815;K00816;K00819;K00824;K00825;K00838;K00844;K00849;K00853;K00886;K00888;K00889;K00894;K00895;K00900;K00904;K00914;K00915;K00921;K00922;K00936;K00944;K00967;K00975;K00987;K00993;K01006;K01045;K01052;K01065;K01074;K01078;K01083;K01099;K01102;K01103;K01108;K01109;K01110;K01120;K01125;K01127;K01130;K01134;K01135;K01136;K01137;K01146;K01153;K01154;K01158;K01161;K01164;K01177;K01184;K01188;K01190;K01192;K01193;K01198;K01201;K01206;K01213;K01219;K01223;K01227;K01228;K01247;K01254;K01258;K01266;K01270;K01271;K01278;K01279;K01285;K01288;K01297;K01299;K01302;K01305;K01309;K01356;K01360;K01366;K01373;K01381;K01399;K01404;K01410;K01426;K01444;K01456;K01479;K01482;K01490;K01510;K01514;K01517;K01527;K01530;K01534;K01552;K01565;K01578;K01593;K01620;K01629;K01661;K01685;K01686;K01727;K01729;K01758;K01764;K01785;K01796;K01799;K01804;K01805;K01812;K01815;K01827;K01838;K01853;K01855;K01900;K01904;K01911;K01922;K01930;K01941;K01948;K01995;K01996;K01997;K01998;K01999;K02087;K02100;K02126;K02133;K02134;K02136;K02137;K02144;K02145;K02146;K02147;K02148;K02149;K02150;K02154;K02155;K02179;K02180;K02183;K02209;K02210;K02212;K02214;K02236;K02242;K02256;K02258;K02260;K02261;K02262;K02321;K02324;K02325;K02349;K02350;K02361;K02365;K02374;K02429;K02477;K02478;K02480;K02502;K02513;K02516;K02529;K02532;K02533;K02537;K02541;K02542;K02551;K02554;K02560;K02565;K02585;K02586;K02587;K02591;K02592;K02594;K02596;K02597;K02608;K02641;K02658;K02682;K02684;K02685;K02726;K02728;K02729;K02730;K02731;K02734;K02736;K02737;K02750;K02757;K02779;K02825;K02826;K02839;K02856;K02868;K02870;K02871;K02873;K02909;K02911;K02918;K02920;K02932;K02934;K02937;K02941;K02942;K02951;K02953;K02957;K02963;K02964;K02969;K02973;K02981;K02986;K02993;K02999;K03002;K03005;K03008;K03018;K03019;K03020;K03022;K03023;K03028;K03029;K03031;K03033;K03035;K03037;K03038;K03039;K03048;K03061;K03063;K03064;K03065;K03066;K03077;K03083;K03089;K03099;K03103;K03107;K03108;K03114;K03115;K03127;K03131;K03134;K03139;K03141;K03142;K03143;K03150;K03153;K03155;K03165;K03169;K03173;K03174;K03178;K03206;K03220;K03239;K03240;K03247;K03249;K03250;K03251;K03252;K03253;K03254;K03256;K03311;K03325;K03328;K03339;K03341;K03343;K03345;K03348;K03349;K03354;K03355;K03358;K03363;K03364;K03366;K03372;K03374;K03377;K03379;K03427;K03437;K03456;K03462;K03485;K03509;K03511;K03514;K03515;K03526;K03529;K03534;K03568;K03606;K03638;K03640;K03661;K03678;K03681;K03700;K03735;K03748;K03804;K03818;K03844;K03847;K03849;K03850;K03859;K03860;K03869;K03872;K03878;K03879;K03880;K03881;K03882;K03883;K03923;K03930;K03934;K03935;K03936;K03940;K03942;K03953;K04020;K04031;K04041;K04068;K04072;K04088;K04101;K04345;K04349;K04354;K04368;K04371;K04373;K04392;K04411;K04412;K04421;K04457;K04461;K04464;K04499;K04505;K04516;K04523;K04532;K04534;K04536;K04566;K04575;K04603;K04646;K04648;K04679;K04711;K04714;K04728;K04733;K04740;K04758;K04770;K04806;K04807;K04833;K04850;K04851;K04856;K04859;K04958;K04959;K04960;K04961;K04962;K04963;K04966;K04972;K04975;K04986;K04988;K05012;K05015;K05016;K05019;K05035;K05040;K05186;K05222;K05236;K05284;K05288;K05290;K05305;K05349;K05361;K05367;K05389;K05499;K05502;K05522;K05528;K05531;K05542;K05543;K05546;K05601;K05603;K05609;K05610;K05616;K05617;K05642;K05643;K05658;K05663;K05665;K05667;K05668;K05672;K05673;K05674;K05680;K05681;K05699;K05713;K05714;K05720;K05724;K05727;K05733;K05740;K05749;K05750;K05752;K05754;K05755;K05756;K05757;K05758;K05765;K05768;K05775;K05798;K05809;K05860;K05861;K05863;K05864;K05872;K05951;K05954;K05989;K06027;K06037;K06047;K06062;K06063;K06067;K06091;K06103;K06109;K06111;K06125;K06169;K06171;K06172;K06185;K06204

K06211;K06268;K06270;K06276;K06316;K06330;K06350;K06580;K06630;K06631;K06632;K06633;K06636;K06639;K06640;K06642;K06643;K06655;K06669;K06672;K06674;K06675;K06677;K06682;
K06685;K06691;K06693;K06694;K06767;K06812;K06837;K06866;K06878;K06894;K06902;K06909;K06910;K06929;K06949;K07013;K07024;K07050;K07075;K07085;K07137;K07165;K07198;K07200;
K07203;K07204;K07213;K07227;K07248;K07252;K07269;K07284;K07317;K07318;K07326;K07338;K07359;K07365;K07375;K07376;K07404;K07432;K07436;K07439;K07440;K07441;K07450;K07483;
K07485;K07489;K07509;K07514;K07515;K07542;K07556;K07674;K07683;K07705;K07727;K07734;K07736;K07738;K07741;K07746;K07748;K07768;K07777;K07784;K07796;K07819;K07821;K07827;
K07877;K07884;K07885;K07897;K07901;K07903;K07905;K07915;K07925;K07931;K07937;K07938;K07943;K07953;K07962;K07977;K08017;K08054;K08057;K08059;K08065;K08069;K08073;K08074;
K08092;K08106;K08138;K08139;K08142;K08150;K08171;K08191;K08194;K08220;K08239;K08264;K08266;K08267;K08269;K08272;K08285;K08286;K08287;K08288;K08292;K08330;K08332;K08333;
K08334;K08343;K08360;K08364;K08467;K08476;K08486;K08490;K08493;K08496;K08504;K08518;K08568;K08576;K08582;K08591;K08592;K08597;K08647;K08653;K08654;K08658;K08676;K08715;
K08730;K08734;K08737;K08739;K08740;K08741;K08762;K08770;K08777;K08793;K08794;K08798;K08805;K08813;K08819;K08823;K08825;K08827;K08836;K08837;K08838;K08844;K08850;K08856;
K08857;K08863;K08870;K08872;K08874;K08955;K08956;K08961;K08974;K08976;K08978;K09008;K09011;K09116;K09125;K09164;K09188;K09228;K09258;K09313;K09377;K09414;K09415;K09419;
K09422;K09423;K09476;K09486;K09487;K09489;K09490;K09492;K09493;K09494;K09495;K09496;K09497;K09498;K09499;K09500;K09502;K09504;K09506;K09517;K09518;K09522;K09529;K09533;
K09548;K09549;K09553;K09554;K09561;K09562;K09568;K09569;K09577;K09584;K09592;K09595;K09613;K09645;K09647;K09649;K09651;K09660;K09666;K09685;K09704;K09740;K09780;K09790;
K09796;K09805;K09826;K09848;K09849;K09867;K09896;K09909;K09925;K09935;K09938;K09940;K09951;K09952;K09953;K09955;K09958;K09974;K10082;K10085;K10086;K10088;K10106;K10133;
K10134;K10141;K10144;K10203;K10225;K10237;K10244;K10251;K10256;K10257;K10263;K10268;K10270;K10277;K10279;K10290;K10295;K10317;K10356;K10357;K10358;K10359;K10364;K10365;
K10389;K10390;K10392;K10394;K10395;K10396;K10397;K10398;K10399;K10400;K10401;K10405;K10406;K10408;K10409;K10410;K10413;K10414;K10417;K10418;K10420;K10426;K10436;K10447;
K10457;K10523;K10569;K10577;K10579;K10581;K10583;K10589;K10592;K10595;K10598;K10599;K10606;K10610;K10615;K10626;K10631;K10632;K10643;K10645;K10661;K10679;K10685;K10686;
K10692;K10699;K10706;K10712;K10734;K10738;K10744;K10745;K10746;K10750;K10752;K10753;K10765;K10766;K10770;K10772;K10777;K10779;K10799;K10800;K10802;K10823;K10824;K10838;
K10839;K10841;K10842;K10846;K10858;K10865;K10866;K10867;K10870;K10875;K10876;K10884;K10891;K10916;K10949;K10950;K10973;K11062;K11086;K11090;K11092;K11093;K11094;K11096;
K11098;K11103;K11124;K11126;K11129;K11135;K11136;K11137;K11143;K11155;K11161;K11168;K11170;K11204;K11231;K11240;K11251;K11254;K11262;K11268;K11269;K11273;K11279;K11293;
K11294;K11303;K11308;K11314;K11320;K11323;K11324;K11339;K11340;K11346;K11364;K11367;K11374;K11380;K11393;K11411;K11412;K11416;K11421;K11425;K11434;K11438;K11462;K11463;
K11481;K11490;K11491;K11492;K11498;K11526;K11538;K11547;K11548;K11564;K11577;K11583;K11584;K11592;K11593;K11594;K11644;K11647;K11654;K11662;K11665;K11684;K11713;K11718;
K11721;K11723;K11742;K11743;K11748;K11750;K11762;K11770;K11783;K11786;K11795;K11798;K11804;K11805;K11806;K11808;K11824;K11826;K11835;K11836;K11837;K11838;K11839;K11840;
K11844;K11848;K11853;K11855;K11856;K11857;K11859;K11864;K11874;K11877;K11883;K11884;K11902;K11922;K11940;K11964;K11967;K11968;K11976;K11981;K11984;K11992;K12021;K12026;
K12160;K12173;K12175;K12176;K12177;K12182;K12188;K12189;K12190;K12191;K12197;K12199;K12200;K12243;K12245;K12257;K12265;K12272;K12275;K12301;K12305;K12306;K12307;K12316;
K12319;K12323;K12326;K12348;K12378;K12384;K12385;K12386;K12389;K12391;K12393;K12396;K12397;K12398;K12399;K12402;K12403;K12417;K12418;K12447;K12450;K12451;K12462;K12471;
K12478;K12479;K12483;K12486;K12491;K12492;K12493;K12500;K12525;K12544;K12558;K12559;K12567;K12571;K12572;K12580;K12581;K12585;K12586;K12587;K12590;K12591;K12592;K12600;
K12603;K12605;K12607;K12614;K12620;K12622;K12623;K12625;K12655;K12658;K12661;K12662;K12666;K12735;K12739;K12811;K12812;K12813;K12815;K12816;K12817;K12818;K12819;K12820;
K12821;K12822;K12823;K12825;K12826;K12827;K12828;K12829;K12830;K12831;K12832;K12833;K12834;K12836;K12837;K12839;K12842;K12843;K12844;K12845;K12847;K12848;K12849;K12850;
K12854;K12856;K12858;K12859;K12860;K12861;K12862;K12863;K12864;K12867;K12868;K12869;K12871;K12872;K12873;K12874;K12876;K12877;K12879;K12880;K12881;K12883;K12890;K12945;
K12947;K12975;K12989;K12998;K13017;K13024;K13025;K13026;K13043;K13047;K13055;K13058;K13091;K13095;K13100;K13101;K13102;K13103;K13107;

	K13108;K13111;K13113;K13115;K13116;K13117;K13118;K13120;K13121;K13123;K13124;K13126;K13130;K13141;K13143;K13154;K13176;K13177;K13179;K13192;K13205;K13206;K13207;K13210;K13220;K13254;K13282;K13289;K13298;K13303;K13304;K13305;K13338;K13339;K13347;K13348;K13370;K13411;K13421;K13431;K13444;K13479;K13511;K13512;K13532;K13577;K13617;K13621;K13653;K13676;K13694;K13696;K13699;K13704;K13707;K13708;K13726;K13749;K13754;K13775;K13783;K13788;K13800;K13806;K13809;K13811;K13832;K13886;K13921;K13922;K13923;K13941;K13946;K13960;K13990;K14001;K14004;K14005;K14006;K14007;K14011;K14012;K14015;K14018;K14050;K14079;K14137;K14157;K14191;K14209;K14213;K14272;K14290;K14292;K14297;K14298;K14299;K14312;K14319;K14326;K14327;K14337;K14342;K14376;K14381;K14386;K14391;K14399;K14401;K14402;K14403;K14404;K14407;K14408;K14411;K14413;K14430;K14437;K14439;K14447;K14454;K14497;K14521;K14536;K14537;K14538;K14539;K14544;K14546;K14548;K14549;K14552;K14555;K14556;K14560;K14561;K14563;K14565;K14566;K14567;K14569;K14570;K14571;K14572;K14573;K14575;K14623;K14638;K14642;K14684;K14685;K14689;K14696;K14713;K14724;K14726;K14754;K14763;K14764;K14766;K14768;K14769;K14771;K14772;K14773;K14774;K14775;K14776;K14777;K14778;K14779;K14780;K14786;K14788;K14791;K14792;K14793;K14794;K14797;K14799;K14801;K14802;K14803;K14805;K14806;K14807;K14808;K14809;K14810;K14811;K14815;K14816;K14819;K14820;K14823;K14824;K14827;K14829;K14830;K14831;K14834;K14835;K14837;K14839;K14842;K14843;K14846;K14848;K14849;K14852;K14856;K14857;K14859;K14861;K14863;K14864;K14944;K14947;K14948;K14950;K14962;K14964;K14981;K14997;K15013;K15015;K15026;K15027;K15028;K15030;K15048;K15054;K15075;K15081;K15085;K15101;K15103;K15106;K15108;K15110;K15111;K15119;K15128;K15146;K15156;K15171;K15172;K15174;K15176;K15177;K15178;K15180;K15190;K15200;K15216;K15227;K15255;K15262;K15263;K15271;K15272;K15275;K15276;K15277;K15278;K15279;K15280;K15287;K15292;K15296;K15304;K15305;K15306;K15315;K15322;K15332;K15333;K15336;K15340;K15342;K15356;K15363;K15370;K15376;K15382;K15414;K15421;K15423;K15424;K15426;K15436;K15437;K15440;K15442;K15443;K15445;K15446;K15448;K15456;K15463;K15477;K15498;K15505;K15507;K15518;K15519;K15533;K15541;K15542;K15553;K15576;K15577;K15578;K15620;K15627;K15631;K15680;K15692;K15693;K15707;K15716;K15726;K15728;K15746;K15778;K15779;K15790;K15791;K15850;K15889;K15890;K15892;K15898;K15921;K15925;K15979;K16054;K16056;K16073;K16089;K16185;K16186;K16196;K16211;K16213;K16263;K16275;K16276;K16295;K16297;K16339;K16345;K16363;K16369;K16455;K16461;K16470;K16474;K16478;K16487;K16491;K16494;K16495;K16506;K16533;K16536;K16547;K16550;K16569;K16570;K16575;K16576;K16578;K16583;K16593;K16600;K16601;K16606;K16609;K16669;K16675;K16692;K16694;K16697;K16699;K16700;K16705;K16724;K16732;K16740;K16743;K16747;K16748;K16780;K16781;K16802;K16803;K16812;K16841;K16845;K16846;K16865;K17043;K17081;K17086;K17087;K17095;K17255;K17261;K17262;K17263;K17267;K17279;K17286;K17290;K17301;K17302;K17361;K17408;K17413;K17427;K17428;K17430;K17496;K17498;K17506;K17508;K17543;K17544;K17553;K17579;K17580;K17583;K17600;K17601;K17604;K17605;K17606;K17608;K17609;K17610;K17617;K17619;K17632;K17637;K17677;K17679;K17681;K17707;K17713;K17726;K17733;K17744;K17751;K17757;K17761;K17775;K17785;

	K17790;K17794;K17808;K17816;K17817;K17820;K17866;K17867;K17868;K17878;K17888;K17907;K17914;K17916;K17917;K17951;K17972;K17987;K17988;K18009;K18043;K18046;K18050;K18051;<br>K18061;K18065;K18081;K18082;K18083;K18121;K18134;K18139;K18146;K18148;K18156;K18158;K18159;K18167;K18168;K18170;K18234;K18272;K18283;K18289;K18309;K18333;K18399;K18404;<br>K18410;K18418;K18422;K18423;K18441;K18442;K18443;K18457;K18458;K18460;K18464;K18465;K18466;K18468;K18470;K18478;K18552;K18584;K18588;K18595;K18597;K18598;K18619;K18624;<br>K18643;K18649;K18657;K18663;K18665;K18726;K18740;K18749;K18752;K18758;K18763;K18782;K18787;K18815;K18819;K18831;K18849;K18889;K18953;K18954;K18993;K18995;K19001;K19006;<br>K19027;K19029;K19049;K19054;K19092;K19103;K19104;K19127;K19128;K19129;K19130;K19165;K19166;K19175;K19176;K19178;K19181;K19199;K19219;K19225;K19266;K19269;K19300;K19306;<br>K19307;K19327;K19352;K19355;K19371;K19384;K19398;K19399;K19427;K19431;K19449;K19469;K19476;K19498;K19503;K19525;K19531;K19532;K19539;K19572;K19584;K19603;K19607;K19612;<br>K19619;K19656;K19672;K19673;K19674;K19676;K19678;K19679;K19680;K19681;K19682;K19683;K19685;K19693;K19701;K19716;K19719;K19730;K19750;K19751;K19753;K19754;K19756;K19757;<br>K19758;K19759;K19760;K19801;K19868;K19937;K19941;K19942;K19949;K19951;K19955;K19983;K19985;K19993;K19998;K20023;K20029;K20031;K20047;K20072;K20096;K20099;K20129;K20131;<br>K20151;K20161;K20163;K20164;K20165;K20166;K20167;K20168;K20178;K20179;K20180;K20181;K20182;K20183;K20184;K20196;K20198;K20221;K20222;K20223;K20224;K20241;K20242;K20251;<br>K20278;K20283;K20284;K20289;K20291;K20295;K20296;K20299;K20300;K20305;K20308;K20310;K20318;K20346;K20347;K20360;K20362;K20367;K20370;K20402;K20404;K20405;K20407;K20408;<br>K20409;K20471;K20495;K20536;K20548;K20549;K20604;K20607;K20642;K20649;K20742;K20791;K20794;K20830;K20843;K20865;K20866;K20868;K20876;K20879;K20885;K20888;K20892;K20920;<br>K20956;K20966;K20968;K21022;K21055;K21138;K21157;K21217;K21279;K21343;K21345;K21358;K21396;K21398;K21411;K21415;K21421;K21462;K21486;K21552;K21562;K21568;K21571;K21572;<br>K21573;K21574;K21594;K21607;K21618;K21694;K21735;K21737;K21750;K21763;K21766;K21767;K21768;K21797;K21798;K21805;K21806;K21813;K21828;K21829;K21850;K21853;K21868;K21916;<br>K21919;K21936;K21988;K21991;K21993;K22017;K22041;K22051;K22071;K22074;K22078;K22106;K22125;K22136;K22139;K22182;K22187;K22262;K22351;K22369;K22377;K22381;K22399;K22418;<br>K22419;K22441;K22515;K22523;K22530;K22544;K22556;K22558;K22560;K22563;K22564;K22582;K22593;K22644;K22647;K22664;K22666;K22684;K22746;K22748;K22754;K22761;K22766;K22767;<br>K22768;K22803;K22804;K22809;K22842;K22857;K22858;K22864;K22866;K22868;K22913;K22937;K22939;K22943;K22987;K23002;K23029;K23040;K23113;K23114;K23159;K23240;K23242;K23288;<br>K23289;K23298;K23309;K23314;K23327;K23329;K23341;K23343;K23355;K23358;K23379;K23383;K23435;K23437;K23438;K23456;K23460;K23485;K23490;K23503;K23538;K23544;K23553;K23565;<br>K23568;K23628;K23677;K23700;K23727;K23791;K23802;K23916;K23937;K23965;K23966;K24034;K24083

Module 03	K00002;K00016;K00038;K00055;K00067;K00070;K00082;K00105;K00119;K00121;K00129;K00132;K00141;K00153;K00156;K00158;K00179;K00180;K00193;K00194;K00197;K00198;K00226;K00230;K00231;K00248;K00273;K00274;K00284;K00360;K00363;K00370;K00371;K00373;K00374;K00376;K00441;K00461;K00462;K00467;K00491;K00505;K00512;K00540;K00541;K00570;K00586;K00633;K00638;K00675;K00683;K00688;K00728;K00784;K00833;K00867;K00897;K00917;K00940;K00953;K00968;K00973;K01005;K01007;K01016;K01023;K01027;K01035;K01046;K01049;K01050;K01066;K01067;K01101;K01114;K01138;K01147;K01155;K01195;K01250;K01251;K01286;K01298;K01304;K01361;K01416;K01417;K01461;K01481;K01507;K01531;K01539;K01551;K01554;K01568;K01574;K01575;K01579;K01587;K01615;K01621;K01634;K01643;K01667;K01671;K01697;K01710;K01762;K01790;K01808;K01821;K01844;K01847;K01851;K01860;K01865;K01905;K01965;K01989;K01993;K02006;K02008;K02066;K02067;K02076;K02078;K02084;K02086;K02107;K02164;K02182;K02283;K02305;K02347;K02371;K02381;K02383;K02385;K02446;K02466;K02491;K02499;K02562;K02566;K02574;K02598;K02657;K02768;K02844;K02875;K02917;K02929;K02956;K02974;K02978;K02979;K03042;K03045;K03051;K03053;K03055;K03056;K03090;K03091;K03212;K03268;K03278;K03282;K03293;K03303;K03312;K03324;K03333;K03340;K03342;K03350;K03385;K03420;K03471;K03483;K03537;K03539;K03547;K03549;K03552;K03627;K03653;K03671;K03692;K03698;K03709;K03716;K03724;K03742;K03761;K03762;K03816;K03817;K03822;K03824;K03871;K03889;K03890;K03891;K03892;K03918;K03928;K03933;K04023;K04024;K04026;K04027;K04028;K04032;K04033;K04059;K04063;K04092;K04104;K04116;K04117;K04343;K04459;K04484;K04515;K04517;K04753;K04757;K04767;K04787;K05271;K05310;K05337;K05342;K05356;K05360;K05363;K05364;K05552;K05555;K05558;K05565;K05587;K05614;K05657;K05709;K05711;K05797;K05805;K05825;K05846;K05888;K05917;K05953;K05967;K05991;K06039;K06133;K06148;K06200;K06282;K06298;K06409;K06595;K06603;K06860;K06870;K06873;K06874;K06883;K06886;K06893;K06915;K06928;K06935;K06943;K06945;K06947;K06956;K06960;K06973;K06986;K06994;K07025;K07029;K07030;K07044;K07054;K07062;K07077;K07086;K07093;K07096;K07118;K07123;K07124;K07131;K07138;K07139;K07163;K07169;K07214;K07218;K07230;K07241;K07243;K07260;K07261;K07267;K07273;K07275;K07281;K07302;K07319;K07321;K07345;K07358;K07393;K07395;K07443;K07451;K07464;K07467;K07494;K07496;K07503;K07517;K07533;K07562;K07565;K07576;K07580;K07586;K07646;K07654;K07655;K07669;K07671;K07692;K07696;K07697;K07753;K07763;K07775;K07790;K07793;K07799;K07810;K08022;K08085;K08151;K08168;K08172;K08177;K08195;K08219;K08225;K08253;K08256;K08260;K08276;K08281;K08298;K08302;K08368;K08481;K08602;K08642;K08651;K08680;K08690;K08716;K08717;K08764;K08969;K08970;K08971;K08977;K08980;K08996;K08999;K09065;K09150;K09153;K09157;K09165;K09252;K09388;K09456;K09488;K09523;K09705;K09728;K09743;K09746;K09753;K09758;K09770;K09772;K09793;K09795;K09797;K09818;K09820;K09825;K09829;K09835;K09843;K09874;K09892;K09915;K09939;K09946;K09984;K10010;K10039;K10040;K10206;K10211;K10252;K10275;K10297;K10342;K10473;K10528;K10533;K10536;K10619;K10700;K10762;K10780;K10837;K10855;K10856;K10907;K10944;K10945;K10946;K11014;K11029;K11147;K11159;K11180;K11181;K11249;K11259;K11263;K11264;K11325;K11390;K11410;K11418;K11437;K11528;K11603;K11618;K11645;K11740;K11741;K11745;K11782;K11816;K11822;K11931;K11942;K11949;K12062;K12066;K12069;K12264;K12405;K12420;K12431;K12510;K12528;K12529;K12539;K12548;K12555;K12583;K12601;K12602;K12940;K12954;K12956;K12962;K12963;K12987;K12994;K13021;K13057;K13238;K13252;K13275;K13315;K13449;K13530;K13542;K13570;K13572;K13573;K13580;K13586;K13622;K13623;K13628;K13644;K13661;K13675;K13746;K13774;K13794;K13810;K13812;K13815;K13830;K13859;K13926;K13933;K13940;K13948;K13955;K13988;K14054;K14059;K14084;K14138;K14164;K14194;K14201;K14335;K14339;K14340;K14379;K14465;K14520;K14580;K14599;K14600;K14611;K14645;K14657;K14661;K14667;K14683;K14709;K14730;K14818;K14826;K14941;K14956;K14979;K15033;K15034;K15232;K15238;K15253;K15256;K15408;K15422;K15444;K15450;K15520;K15524;K15526;K15538;K15547;K15580;K15581;K15582;K15629;K15642;K15720;K15725;K15727;K15733;K15781;K15789;K15825;K15836;K15861;K15862;K15868;K15876

	;K15878;K15879;K15891;K15915;K15916;K15922;K16020;K16028;K16029;K16046;K16055;K16079;K16081;K16137;K16138;K16149;K16150;K16157;K16158;K16160;K16161;K16163;K16168;K16171;K16173;K16190;K16199;K16239;K16247;K16303;K16318;K16331;K16382;K16383;K16422;K16435;K16545;K16563;K16567;K16629;K16648;K16693;K16785;K16786;K16787;K16788;K16815;K16839;K16885;K16886;K16887;K16923;K16927;K16937;K16944;K17048;K17070;K17071;K17250;K17318;K17319;K17320;K17457;K17464;K17484;K17488;K17768;K17803;K17829;K17835;K17840;K17870;K17883;K17991;K17995;K18011;K18013;K18017;K18049;K18098;K18105;K18199;K18200;K18223;K18224;K18225;K18226;K18231;K18282;K18304;K18332;K18351;K18352;K18353;K18367;K18376;K18383;K18467;K18562;K18572;K18660;K18672;K18677;K18688;K18697;K18818;K18845;K18848;K18890;K18939;K18958;K19014;K19031;K19118;K19119;K19131;K19132;K19136;K19169;K19170;K19171;K19172;K19190;K19220;K19222;K19223;K19224;K19234;K19267;K19280;K19329;K19335;K19339;K19342;K19417;K19421;K19511;K19515;K19516;K19550;K19569;K19578;K19587;K19686;K19687;K19689;K19706;K19713;K19737;K19820;K19823;K19837;K19982;K20039;K20201;K20203;K20238;K20246;K20260;K20262;K20266;K20433;K20444;K20458;K20468;K20469;K20470;K20484;K20486;K20497;K20528;K20529;K20530;K20531;K20533;K20539;K20542;K20810;K20921;K20997;K20999;K21000;K21065;K21101;K21106;K21147;K21148;K21160;K21169;K21170;K21171;K21173;K21174;K21190;K21224;K21258;K21259;K21260;K21262;K21271;K21298;K21302;K21304;K21328;K21333;K21335;K21349;K21350;K21397;K21441;K21457;K21463;K21464;K21470;K21472;K21474;K21481;K21515;K21591;K21600;K21603;K21685;K21688;K21727;K21730;K21747;K21757;K21772;K21825;K21834;K21893;K21935;K21949;K21961;K21962;K22043;K22107;K22108;K22112;K22144;K22227;K22294;K22306;K22318;K22320;K22336;K22341;K22347;K22363;K22364;K22408;K22446;K22463;K22474;K22502;K22516;K22580;K22616;K22617;K22618;K22723;K22757;K22769;K22820;K22886;K22893;K22901;K22902;K22905;K22928;K22934;K22968;K23077;K23118;K23121;K23138;K23139;K23149;K23164;K23176;K23239;K23258;K23337;K23371;K23446;K23498;K23504;K23686;K23734;K23735;K23753;K23778;K23812;K23825;K23842;K23948;K23983;K24017;K24029;K24033;K24040;K24073;K24078;K24097;K24102

Module 04	K00008;K00039;K00185;K00209;K00243;K00290;K00330;K00333;K00337;K00338;K00341;K00346;K00347;K00349;K00350;K00351;K00448;K00661;K00772;K00797;K00809;K00821;K00850;K00854;K00874;K00876;K00902;K00906;K00913;K00925;K00956;K00957;K00972;K01077;K01089;K01148;K01176;K01180;K01187;K01200;K01256;K01273;K01277;K01283;K01284;K01322;K01407;K01412;K01420;K01424;K01431;K01437;K01443;K01505;K01556;K01601;K01611;K01624;K01625;K01682;K01734;K01791;K01811;K01820;K01825;K01835;K01840;K01893;K01926;K01991;K02151;K02202;K02257;K02317;K02318;K02331;K02348;K02495;K02510;K02521;K02552;K02636;K02680;K02738;K02739;K02742;K02843;K02878;K02880;K02913;K02943;K02945;K02950;K02990;K03004;K03014;K03032;K03125;K03144;K03194;K03207;K03258;K03292;K03305;K03322;K03332;K03386;K03406;K03411;K03458;K03591;K03603;K03607;K03654;K03719;K03721;K03746;K03760;K03764;K03772;K03811;K03819;K03831;K03839;K03840;K03841;K03846;K03848;K03856;K03857;K03886;K03972;K04062;K04079;K04498;K04688;K04759;K04802;K05283;K05292;K05302;K05350;K05368;K05590;K05613;K05774;K05780;K05781;K05799;K05803;K05812;K05836;K05896;K05955;K05995;K06006;K06074;K06100;K06163;K06165;K06166;K06175;K06177;K06181;K06182;K06193;K06219;K06634;K06676;K06678;K06709;K06858;K06882;K06959;K06967;K06970;K07010;K07014;K07089;K07106;K07173;K07179;K07255;K07263;K07303;K07313;K07386;K07506;K07640;K07684;K07720;K07751;K07773;K07776;K07934;K07935;K08066;K08094;K08227;K08230;K08282;K08307;K08312;K08325;K08372;K08485;K08675;K08692;K08735;K08775;K08830;K08852;K08864;K08968;K08981;K08989;K09015;K09158;K09167;K09291;K09537;K09567;K09658;K09694;K09695;K09819;K09893;K09894;K09899;K09904;K09913;K09927;K09975;K09982;K10235;K10249;K10258;K10391;K10411;K10572;K10736;K10737;K10843;K10896;K10899;K10903;K10974;K11108;K11292;K11322;K11338;K11348;K11373;K11426;K11430;K11446;K11692;K11755;K11757;K11850;K11927;K12152;K12345;K12349;K12400;K12454;K12472;K12524;K12604;K12606;K12611;K12736;K12835;K12852;K12855;K12898;K12961;K12968;K12991;K12993;K13016;K13051;K13096;K13110;K13125;K13151;K13181;K13239;K13250;K13277;K13498;K13506;K13534;K13637;K13711;K13807;K13829;K13924;K13938;K13989;K14026;K14067;K14187;K14288;K14293;K14318;K14416;K14424;K14441;K14550;K14692;K14767;K14790;K14832;K14833;K14841;K14844;K14965;K15029;K15040;K15102;K15181;K15196;K15264;K15281;K15283;K15335;K15362;K15451;K15460;K15501;K15528;K15668;K15782;K15986;K16090;K16256;K16257;K16259;K16458;K16465;K16538;K16605;K16745;K16749;K16810;K17224;K17278;K17541;K17570;K17592;K17616;K17618;K17732;K17737;K17800;K17890;K17943;K18143;K18345;K18426;K18469;K18627;K18820;K18893;K18902;K18903;K18932;K19000;K19073;K19163;K19226;K19227;K19228;K19229;K19230;K19347;K19348;K19513;K19611;K19671;K19677;K19684;K19752;K19788;K19793;K19802;K19984;K20028;K20074;K20286;K20293;K20298;K20352;K20449;K20525;K20996;K21393;K21395;K21564;K21575;K21596;K21703;K21744;K21776;K21852;K21900;K22048;K22066;K22077;K22145;K22252;K22316;K22334;K22409;K22476;K22489;K22531;K22762;K22985;K23025;K23123;K23124;K23286;K23330;K23333;K23338;K23466;K23539;K23562;K23741

Module 05	K00044;K00048;K00112;K00187;K00189;K00223;K00257;K00279;K00365;K00466;K00468;K00514;K00524;K00527;K00622;K00686;K00695;K00756;K00814;K00938;K00977;K00994;K01013;K01128;K01131;K01160;K01167;K01183;K01312;K01337;K01363;K01394;K01400;K01402;K01405;K01468;K01546;K01547;K01548;K01557;K01567;K01616;K01655;K01699;K01705;K01712;K01731;K01745;K01914;K01957;K02088;K02091;K02101;K02241;K02471;K02539;K02599;K02805;K02821;K02854;K02872;K02882;K02938;K02947;K02955;K02960;K02985;K02989;K02997;K03068;K03436;K03475;K03715;K03736;K03758;K03792;K03867;K03897;K03922;K04018;K04093;K04374;K04449;K04460;K04508;K04558;K04659;K04734;K04739;K04780;K04781;K04784;K04788;K04789;K04790;K04791;K04792;K04793;K05190;K05308;K05370;K05517;K05551;K05573;K05692;K05698;K05716;K05759;K05770;K05826;K05871;K05873;K05874;K05881;K05887;K05901;K05926;K05942;K06223;K06229;K06238;K06254;K06320;K06338;K06412;K06505;K06506;K06520;K06530;K06540;K06585;K06609;K06751;K06849;K06934;K06974;K07009;K07033;K07057;K07066;K07074;K07224;K07346;K07453;K07480;K07487;K07548;K07606;K07647;K07656;K07672;K07698;K07701;K07769;K07779;K07788;K07789;K07876;K07888;K07918;K07920;K08005;K08095;K08100;K08105;K08166;K08241;K08326;K08369;K08446;K08779;K08807;K08811;K08855;K08986;K09124;K09133;K09146;K09166;K09478;K09676;K09712;K09732;K09741;K09822;K09857;K09879;K09942;K09957;K09999;K10005;K10006;K10009;K10014;K10030;K10123;K10231;K10538;K10628;K10697;K10709;K10793;K11071;K11081;K11082;K11083;K11084;K11089;K11145;K11252;K11257;K11275;K11329;K11383;K11385;K11386;K11387;K11422;K11433;K11494;K11520;K11521;K11522;K11533;K11534;K11608;K11609;K11611;K11616;K11635;K11725;K11737;K11738;K11739;K11825;K11890;K11892;K11897;K11898;K11899;K11914;K11917;K11923;K11930;K11939;K11943;K11944;K11947;K11987;K12073;K12172;K12240;K12242;K12342;K12421;K12422;K12423;K12426;K12427;K12428;K12433;K12434;K12436;K12437;K12440;K12441;K12443;K12444;K12466;K12526;K12649;K12710;K12741;K12757;K12950;K12951;K12953;K12955;K12981;K12983;K12990;K13040;K13041;K13090;K13137;K13158;K13159;K13162;K13178;K13195;K13219;K13276;K13308;K13310;K13326;K13379;K13381;K13439;K13489;K13501;K13522;K13540;K13654;K13657;K13658;K13659;K13662;K13670;K13686;K13721;K13820;K13823;K13824;K13861;K13919;K13920;K13928;K13929;K13930;K13931;K13934;K13935;K13957;K14019;K14029;K14051;K14052;K14136;K14188;K14192;K14215;K14242;K14275;K14313;K14338;K14384;K14410;K14440;K14443;K14618;K14631;K14633;K14680;K14698;K14699;K14721;K14743;K14840;K14949;K14952;K14953;K14954;K14955;K15113;K15230;K15240;K15320;K15337;K15355;K15361;K15395;K15471;K15474;K15495;K15496;K15643;K15670;K15729;K15784;K15849;K15855;K15919;K15972;K16016;K16045;K16070;K16131;K16151;K16193;K16216;K16301;K16378;K16396;K16397;K16548;K16610;K16645;K16647;K16649;K16666;K16695;K16792;K16794;K16795;K16920;K16938;K17067;K17245;K17283;K17328;K17717;K17734;K17742;K17755;K17756;K17850;K17876;K17880;K17892;K17915;K17961;K17986;K18045;K18093;K18100;K18141;K18201;K18215;K18248;K18251;K18252;K18254;K18255;K18256;K18257;K18264;K18275;K18286;K18288;K18294;K18445;K18447;K18472;K18481;K18540;K18545;K18609;K18662;K18671;K18701;K18781;K18785;K18830;K18851;K18913;K18925;K18956;K18957;K19015;K19051;K19076;K19142;K19246;K19271;K19283;K19284;K19303;K19304;K19309;K19350;K19373;K19433;K19540;K19563;K19666;K19777;K19783;K19787;K19811;K20022;K20024;K20051;K20110;K20162;K20257;K20273;K20331;K20416;K20445;K20466;K20467;K20611;K20616;K20628;K20719;K20761;K20777;K20788;K20813;K20838;K20860;K20900;K20901;K20910;K20937;K20944;K20948;K21073;K21161;K21164;K21166;K21188;K21191;K21199;K21200;K21264;K21286;K21431;K21442;K21473;K21479;K21483;K21489;K21601;K21608;K21689;K21691;K21695;K21739;K21746;K21793;K21836;K21886;K21887;K21904;K21960;K21977;K21992;K22103;K22221;K22222;K22233;K22295;K22309;K22339;K22444;K22450;K22505;K22552;K22690;K22692;K22795;K22796;K22797;K22821;K22822;K22847;K22908;K22957;K23095;K23136;K23151;K23156;K23183;K23188;K23274;K23293;K23301;K23302;K23319;K23361;K23393;K23414;K23416;K23558;K23776;K24002;K24070;K24084
Module 06	K00648;K00848;K01263;K03202;K06079;K06415;K07098;K07685;K07910;K08682;K09992;K11066;K11354;K11918;K12972;K14963;K15899;K18145;K19036;K21786;K22503;K22686

Module 07	K00003;K00007;K00012;K00013;K00014;K00018;K00019;K00020;K00021;K00022;K00028;K00029;K00031;K00033;K00036;K00043;K00053;K00057;K00060;K00075;K00076;K00083;K00097;K00101;K00108;K00109;K00111;K00113;K00116;K00122;K00123;K00128;K00130;K00133;K00134;K00138;K00140;K00142;K00145;K00147;K00151;K00154;K00161;K00163;K00164;K00171;K00176;K00177;K00207;K00215;K00219;K00228;K00232;K00244;K00253;K00254;K00262;K00264;K00265;K00266;K00275;K00278;K00281;K00286;K00287;K00294;K00295;K00297;K00309;K00311;K00315;K00316;K00322;K00324;K00325;K00336;K00348;K00354;K00381;K00382;K00383;K00404;K00405;K00411;K00412;K00413;K00414;K00417;K00427;K00432;K00446;K00449;K00452;K00457;K00477;K00481;K00499;K00511;K00523;K00525;K00526;K00554;K00557;K00560;K00563;K00564;K00566;K00569;K00587;K00588;K00589;K00595;K00602;K00605;K00616;K00619;K00620;K00625;K00627;K00631;K00632;K00640;K00641;K00643;K00645;K00647;K00651;K00655;K00658;K00674;K00677;K00679;K00684;K00690;K00705;K00706;K00748;K00758;K00766;K00769;K00773;K00783;K00790;K00791;K00795;K00800;K00806;K00813;K00817;K00820;K00830;K00831;K00845;K00846;K00847;K00852;K00855;K00859;K00864;K00865;K00871;K00873;K00878;K00884;K00901;K00912;K00919;K00927;K00930;K00931;K00937;K00942;K00951;K00963;K00965;K00969;K00970;K00971;K00974;K00979;K00982;K00989;K00990;K00997;K01000;K01004;K01031;K01032;K01047;K01048;K01056;K01057;K01058;K01075;K01076;K01079;K01081;K01086;K01091;K01092;K01115;K01118;K01129;K01132;K01139;K01141;K01142;K01156;K01163;K01175;K01182;K01194;K01207;K01230;K01243;K01255;K01262;K01264;K01267;K01280;K01318;K01341;K01345;K01359;K01371;K01390;K01408;K01409;K01411;K01414;K01419;K01427;K01428;K01434;K01438;K01439;K01448;K01465;K01476;K01477;K01483;K01484;K01485;K01486;K01487;K01488;K01494;K01497;K01522;K01524;K01525;K01529;K01533;K01571;K01573;K01580;K01584;K01585;K01586;K01589;K01597;K01599;K01602;K01610;K01613;K01619;K01623;K01626;K01627;K01633;K01637;K01638;K01640;K01649;K01657;K01659;K01662;K01663;K01665;K01666;K01669;K01673;K01676;K01687;K01690;K01714;K01716;K01719;K01735;K01739;K01740;K01756;K01772;K01775;K01778;K01779;K01782;K01783;K01792;K01798;K01800;K01810;K01823;K01839;K01857;K01867;K01868;K01869;K01870;K01872;K01873;K01874;K01876;K01878;K01879;K01881;K01886;K01887;K01890;K01892;K01894;K01907;K01908;K01915;K01916;K01918;K01919;K01924;K01925;K01928;K01929;K01934;K01937;K01938;K01942;K01945;K01946;K01952;K01955;K01958;K01960;K01961;K01962;K01963;K01968;K01969;K01972;K02010;K02011;K02012;K02017;K02018;K02019;K02020;K02028;K02031;K02033;K02036;K02037;K02038;K02039;K02040;K02052;K02053;K02055;K02062;K02063;K02064;K02074;K02075;K02108;K02109;K02110;K02111;K02112;K02113;K02114;K02115;K02116;K02138;K02140;K02160;K02167;K02168;K02169;K02170;K02171;K02178;K02197;K02198;K02213;K02225;K02228;K02229;K02230;K02231;K02234;K02238;K02259;K02265;K02291;K02294;K02300;K02302;K02313;K02314;K02316;K02335;K02336;K02337;K02339;K02340;K02341;K02342;K02343;K02346;K02355;K02356;K02357;K02358;K02363;K02372;K02405;K02406;K02407;K02409;K02428;K02439;K02440;K02441;K02444;K02448;K02449;K02451;K02452;K02462;K02463;K02464;K02469;K02470;K02476;K02481;K02482;K02484;K02486;K02494;K02496;K02498;K02509;K02517;K02519;K02520;K02527;K02535;K02536;K02553;K02558;K02572;K02600;K02604;K02606;K02607;K02609;K02610;K02611;K02612;K02613;K02614;K02616;K02617;K02618;K02619;K02621;K02622;K02623;K02624;K02634;K02655;K02656;K02663;K02664;K02665;K02666;K02673;K02676;K02687;K02689;K02690;K02691;K02692;K02694;K02699;K02701;K02704;K02705;K02707;K02709;K02714;K02717;K02720;K02735;K02834;K02835;K02836;K02837;K02838;K02847;K02851;K02860;K02865;K02874;K02879;K02884;K02886;K02887;K02891;K02897;K02926;K02935;K02939;K02940;K02944;K02946;K02954;K02968;K02970;K02971;K02983;K02992;K03000;K03012;K03025;K03026;K03030;K03036;K03043;K03050;K03060;K03062;K03070;K03071;K03072;K03073;K03074;K03075;K03079;K03087;K03092;K03098;K03100;K03101;K03104;K03118;K03126;K03128;K03137;K03138;K03152;K03168;K03177;K03179;K03181;K03184;K03185;K03192;K03215;K03216;K03217;K03218;K03241;K03245;K03269;K03270;K03281;K03287;K03301;K03304;K03310;K03313;K03314;K03317;K03321;K03327;K03351;K03352;K03380;K03387;K03402;K03403;K03404;K03417;K03426;K03428;K03431;K03438;K03439;K03449;K03466;K03472;K03473;K03474;K03495;K03498;K03499;K03500;K03501;K03502;K03503;K03504;K03523;K03525;K03527;K03528;K03530;K03531;K03536;K03542;K03544;K03545;K03548;K03551;K03553;K03554;K03555;K03557;K03558;K03559;K03561;K03562;K03565;K03566;K03569;K03570;K03572;K03573;K03575;K03576;K03578;K03579;K03580;

	K03581;K03582;K03583;K03584;K03586;K03587;K03588;K03589;K03590;K03592;K03595;K03596;K03597;K03598;K03599;K03600;K03601;K03611;K03612;K03613;K03614;K03615;K03616;K03617;K03620;K03621;K03624;K03625;K03628;K03629;K03630;K03631;K03632;K03634;K03635;K03637;K03641;K03644;K03646;K03648;K03650;K03655;K03656;K03657;K03658;K03664;K03667;K03668;K03672;K03673;K03676;K03683;K03685;K03688;K03689;K03690;K03694;K03695;K03701;K03702;K03703;K03710;K03714;K03720;K03723;K03732;K03734;K03745;K03747;K03749;K03750;K03752;K03767;K03770;K03771;K03773;K03774;K03775;K03777;K03778;K03781;K03782;K03786;K03787;K03790;K03796;K03797;K03798;K03801;K03803;K03806;K03807;K03808;K03809;K03812;K03820;K03843;K03845;K03862;K03863;K03893;K03925;K03937;K03946;K03952;K03965;K03966;K03969;K03970;K03973;K03974;K03975;K03976;K03977;K03978;K03979;K03980;K03981;K04035;K04037;K04038;K04039;K04040;K04042;K04043;K04044;K04047;K04066;K04075;K04080;K04082;K04083;K04084;K04090;K04095;K04096;K04100;K04105;K04342;K04344;K04437;K04485;K04486;K04518;K04567;K04568;K04638;K04643;K04649;K04651;K04652;K04653;K04654;K04656;K04681;K04683;K04691;K04708;K04742;K04744;K04754;K04761;K04762;K04764;K04768;K04773;K04774;K04874;K04946;K05046;K05119;K05282;K05285;K05287;K05289;K05291;K05294;K05298;K05304;K05307;K05309;K05365;K05366;K05383;K05464;K05501;K05515;K05520;K05521;K05523;K05526;K05527;K05539;K05540;K05541;K05544;K05559;K05560;K05561;K05562;K05563;K05564;K05589;K05591;K05592;K05641;K05655;K05656;K05712;K05776;K05783;K05786;K05789;K05801;K05807;K05808;K05810;K05820;K05834;K05837;K05838;K05844;K05875;K05895;K05898;K05907;K05913;K05919;K05922;K05927;K05946;K05952;K05973;K05979;K05984;K06001;K06020;K06041;K06049;K06066;K06076;K06077;K06110;K06117;K06118;K06119;K06126;K06127;K06136;K06138;K06147;K06149;K06153;K06156;K06158;K06162;K06168;K06173;K06176;K06178;K06179;K06180;K06183;K06186;K06187;K06189;K06190;K06192;K06194;K06202;K06203;K06205;K06206;K06207;K06234;K06236;K06249;K06443;K06444;K06445;K06447;K06485;K06518;K06569;K06573;K06584;K06601;K06617;K06628;K06662;K06664;K06667;K06670;K06688;K06695;K06699;K06721;K06816;K06861;K06872;K06876;K06879;K06880;K06881;K06891;K06895;K06899;K06916;K06925;K06938;K06941;K06954;K06957;K06966;K06968;K06969;K06972;K06975;K06977;K06980;K06991;K06997;K07000;K07015;K07026;K07039;K07040;K07042;K07043;K07046;K07052;K07053;K07055;K07056;K07070;K07071;K07076;K07078;K07082;K07088;K07090;K07091;K07095;K07097;K07101;K07112;K07113;K07115;K07116;K07120;K07121;K07122;K07133;K07140;K07141;K07146;K07147;K07153;K07156;K07157;K07160;K07164;K07167;K07172;K07175;K07182;K07184;K07199;K07215;K07222;K07223;K07231;K07235;K07236;K07237;K07245;K07251;K07253;K07258;K07262;K07274;K07276;K07277;K07278;K07283;K07285;K07287;K07316;K07320;K07340;K07391;K07394;K07400;K07402;K07407;K07447;K07448;K07454;K07456;K07459;K07462;K07473;K07478;K07481;K07482;K07486;K07493;K07504;K07511;K07552;K07568;K07574;K07589;K07636;K07637;K07638;K07641;K07642;K07643;K07645;K07657;K07660;K07662;K07663;K07666;K07668;K07675;K07677;K07678;K07679;K07689;K07708;K07711;K07712;K07713;K07721;K07726;K07729;K07733;K07740;K07742;K07787;K07794;K07795;K07798;K07908;K07942;K07951;K07995;K08062;K08068;K08077;K08080;K08086;K08093;K08098;K08134;K08154;K08164;K08193;K08217;K08218;K08221;K08224;K08226;K08232;K08242;K08244;K08245;K08261;K08280;K08289;K08299;K08300;K08301;K08305;K08309;K08310;K08311;K08316;K08319;K08323;K08336;K08350;K08472;K08475;K08484;K08492;K08495;K08500;K08503;K08505;K08506;K08511;K08596;K08688;K08700;K08714;K08736;K08749;K08817;K08824;K08876;K08903;K08908;K08911;K08915;K08916;K08927;K08928;K08929;K08973;K08992;K08993;K08998;K09001;K09004;K09007;K09009;K09017;K09060;K09117;K09136;K09139;K09147;K09159;K09160;K09190;K09284;K09285;K09288;K09391;K09470;K09471;K09472;K09480;K09519;K09528;K09531;K09591;K09597;K09673;K09680;K09701;K09703;K09710;K09717;K09744;K09748;K09760;K09761;K09765;K09767;K09768;K09769;K09773;K09775;K09788;K09792;K09794;K09801;K09806;K09811;K09812;K09814;K09823;K09834;K09836;K09837;K09838;K09839;K09844;K09845;K09846;K09847;K09858;K09859;K09860;K09861;K09875;K09883;K09889;K09891;K09895;K09898;K09902;K09906;K09908;K09912;K09916;K09918;K09919;K09920;K09921;K09926;K09928;K09929;K09936;K09941;K09943;K09944;K09950;K09962;K09964;K09966;K09970;K09971;K09972;K09977;K09989;K10027;K10125;K10126;K10143;K10209;K10210;K10218;K10219;K10220;K10221;K10226;K10232;K10233;K10234;K10236;K10260;K10272;K10300;K10353;K10419;K10527;K10570;K10578;K10587;K10588;K10590;K10604;K10627;K10640;K10666;K10691;K10695;K10696;K10704;K10727;K10728;K10730;K10732;K10735;K10739;K10761;K10763;K10764;K10768;K10782;K10804;K10805;K10844;K10847;K10849;K10857;K10862;K10869;K10871;K10872;K10880;K10881;

	K10885;K10912;K10924;K10938;K10941;K10960;K11001;K11004;K11016;K11018;K11075;K11085;K11104;K11107;K11111;K11142;K11153;K11162;K11177;K11179;K11208;K11211;K11250;K11266;K11267;K11271;K11274;K11311;K11333;K11334;K11335;K11336;K11337;K11341;K11353;K11375;K11384;K11391;K11392;K11408;K11417;K11419;K11424;K11450;K11472;K11473;K11474;K11502;K11518;K11527;K11549;K11550;K11599;K11643;K11649;K11650;K11661;K11663;K11685;K11688;K11689;K11690;K11707;K11708;K11709;K11710;K11719;K11720;K11744;K11749;K11752;K11753;K11754;K11778;K11789;K11790;K11793;K11801;K11854;K11858;K11865;K11878;K11886;K11960;K11961;K11962;K11963;K11991;K11996;K12133;K12146;K12169;K12174;K12178;K12179;K12180;K12193;K12235;K12251;K12252;K12262;K12297;K12308;K12340;K12369;K12489;K12506;K12508;K12518;K12551;K12573;K12574;K12617;K12624;K12627;K12657;K12678;K12688;K12700;K12733;K12734;K12737;K12840;K12857;K12870;K12878;K12893;K12941;K12942;K12957;K12974;K12978;K12982;K12996;K13009;K13012;K13038;K13049;K13053;K13059;K13071;K13082;K13098;K13104;K13105;K13106;K13109;K13114;K13119;K13127;K13133;K13165;K13171;K13172;K13174;K13175;K13199;K13211;K13217;K13240;K13247;K13274;K13278;K13288;K13293;K13337;K13344;K13345;K13354;K13378;K13408;K13409;K13427;K13462;K13481;K13482;K13484;K13488;K13509;K13519;K13543;K13600;K13606;K13634;K13638;K13641;K13649;K13652;K13679;K13680;K13682;K13693;K13728;K13735;K13747;K13758;K13766;K13767;K13789;K13795;K13818;K13819;K13821;K13857;K13862;K13888;K13892;K13893;K13894;K13896;K13958;K13963;K13991;K13992;K14014;K14048;K14058;K14060;K14066;K14083;K14155;K14190;K14267;K14273;K14287;K14295;K14300;K14304;K14308;K14310;K14311;K14317;K14325;K14328;K14348;K14393;K14397;K14400;K14436;K14442;K14448;K14449;K14468;K14469;K14470;K14490;K14540;K14541;K14553;K14559;K14610;K14617;K14652;K14664;K14682;K14686;K14715;K14742;K14753;K14782;K14798;K14821;K14822;K14838;K14845;K14847;K14851;K14938;K14961;K14977;K14996;K14998;K15032;K15121;K15151;K15152;K15175;K15192;K15201;K15257;K15285;K15326;K15334;K15338;K15341;K15377;K15396;K15407;K15425;K15428;K15430;K15433;K15441;K15455;K15461;K15511;K15514;K15516;K15527;K15535;K15537;K15544;K15546;K15555;K15559;K15598;K15601;K15622;K15633;K15640;K15665;K15687;K15715;K15723;K15724;K15738;K15747;K15777;K15783;K15785;K15901;K15910;K15912;K15913;K15918;K15975;K15982;K15983;K15984;K16012;K16013;K16048;K16049;K16052;K16053;K16061;K16066;K16076;K16091;K16092;K16129;K16165;K16194;K16219;K16240;K16241;K16258;K16260;K16267;K16287;K16291;K16298;K16305;K16329;K16424;

	K16473;K16489;K16514;K16535;K16540;K16546;K16554;K16571;K16572;K16581;K16615;K16726;K16742;K16744;K16746;K16755;K16757;K16772;K16783;K16784;K16844;K16862;K16870;K16872;K16881;K16911;K17103;K17218;K17222;K17223;K17225;K17229;K17231;K17244;K17247;K17251;K17260;K17268;K17315;K17316;K17317;K17321;K17324;K17422;K17426;K17435;K17491;K17527;K17545;K17552;K17566;K17607;K17613;K17686;K17710;K17735;K17736;K17743;K17747;K17763;K17776;K17795;K17796;K17804;K17822;K17836;K17838;K17879;K17885;K17912;K17925;K17948;K17969;K17973;K17985;K17989;K18010;K18055;K18066;K18106;K18122;K18138;K18164;K18171;K18172;K18182;K18183;K18187;K18203;K18208;K18227;K18263;K18270;K18284;K18301;K18327;K18328;K18335;K18344;K18350;K18364;K18365;K18366;K18372;K18425;K18446;K18456;K18459;K18473;K18477;K18550;K18590;K18592;K18655;K18661;K18666;K18682;K18687;K18691;K18707;K18716;K18729;K18732;K18757;K18778;K18798;K18800;K18810;K18841;K18842;K18850;K18853;K18898;K18930;K18940;K18941;K18968;K18981;K18983;K18988;K18989;K18990;K18999;K19090;K19134;K19158;K19159;K19164;K19231;K19235;K19270;K19311;K19323;K19332;K19333;K19334;K19337;K19353;K19362;K19400;K19416;K19419;K19424;K19478;K19561;K19562;K19589;K19597;K19613;K19710;K19711;K19720;K19736;K19745;K19746;K19789;K19804;K19810;K19826;K19873;K19986;K19995;K19997;K20027;K20034;K20035;K20036;K20069;K20093;K20101;K20115;K20120;K20191;K20195;K20217;K20264;K20274;K20288;K20292;K20294;K20301;K20302;K20353;K20363;K20372;K20393;K20403;K20455;K20456;K20457;K20473;K20474;K20476;K20478;K20483;K20509;K20535;K20547;K20608;K20609;K20715;K20781;K20782;K20783;K20784;K20792;K20800;K20823;K20824;K20826;K20827;K20828;K20858;K20862;K20881;K20884;K20922;K20936;K20940;K20951;K20952;K20964;K20974;K20988;K20993;K20994;K21020;K21034;K21131;K21133;K21136;K21307;K21309;K21363;K21365;K21367;K21379;K21405;K21407;K21420;K21430;K21437;K21449;K21456;K21469;K21478;K21616;K21620;K21624;K21626;K21645;K21676;K21686;K21712;K21734;K21749;K21751;K21752;K21817;K21822;K21826;K21830;K21883;K21899;K21917;K21989;K22020;K22024;K22042;K22047;K22068;K22069;K22086;K22128;K22131;K22132;K22167;K22186;K22193;K22200;K22206;K22218;K22285;K22292;K22300;K22303;K22310;K22314;K22324;K22343;K22344;K22366;K22382;K22384;K22390;K22391;K22443;K22468;K22469;K22479;K22519;K22522;K22553;K22589;K22596;K22597;K22602;K22611;K22622;K22693;K22694;K22698;K22708;K22719;K22756;K22765;K22817;K22819;K22823;K22825;K22869;K22900;K22912;K22940;K22949;K22988;K23007;K23055;K23056;K23057;K23058;K23115;K23146;K23166;K23230;K23234;K23257;K23325;K23351;K23362;K23408;K23455;K23465;K23535;K23536;K23537;K23541;K23543;K23567;K23569;K23570;K23643;K23678;K23693;K23775;K23845;K23856;K23878;K24006;K24018

Module 08	K00035;K00040;K00042;K00115;K00211;K00239;K00241;K00255;K00259;K00342;K00343;K00384;K00474;K00571;K00612;K00693;K00712;K00720;K00760;K00812;K00832;K00841;K00862;K00863;K00868;K00879;K00880;K00899;K00929;K01026;K01071;K01113;K01121;K01178;K01179;K01186;K01209;K01218;K01224;K01310;K01447;K01449;K01491;K01628;K01658;K01681;K01725;K01728;K01818;K01846;K01875;K01939;K02045;K02199;K02204;K02297;K02299;K02319;K02338;K02504;K02525;K02639;K02846;K02903;K03086;K03111;K03195;K03196;K03197;K03198;K03199;K03200;K03201;K03203;K03204;K03205;K03255;K03290;K03326;K03331;K03396;K03407;K03409;K03477;K03490;K03497;K03593;K03608;K03609;K03610;K03618;K03619;K03791;K03805;K03826;K03833;K03941;K04016;K04021;K04045;K04340;K04441;K04477;K04563;K04801;K04940;K05362;K05372;K05661;K05684;K05777;K05778;K05779;K05784;K05791;K05795;K05835;K06073;K06143;K06198;K06281;K06324;K06606;K06607;K06653;K06904;K06905;K06906;K06908;K06919;K06926;K07012;K07092;K07183;K07195;K07265;K07308;K07401;K07452;K07498;K07664;K07693;K07778;K07782;K07862;K07941;K07944;K08070;K08119;K08322;K08351;K08489;K08722;K08744;K08828； K08866;K08994;K09123;K09144;K09539;K09550;K09702;K09789;K09799;K09872;K09897;K09960;K09961;K09980;K09997;K09998;K10002;K10003;K10004;K10016;K10193;K10194;K10195;K10200;K10202;K10543;K10561;K10562;K10563;K10564;K10664;K10708;K10710;K10873;K10901;K10918;K10967;K11041;K11128;K11420;K11537;K11712;K11811;K11863;K11869;K11915;K11959;K12266;K12368;K12371;K12372;K12452;K12980;K13093;K13367;K13500;K13585;K13591;K13613;K13633;K13642;K13663;K13669;K13671;K13730;K13772;K13786;K13855;K13864;K13874;K13875;K13877;K14028;K14160;K14205;K14266;K14274;K14414;K14458;K14489;K14545;K14648;K14673;K14784;K15058;K15059;K15153;K15328;K15329;K15454;K15469;K15531;K15532;K15536;K15545;K15579;K15599;K15600;K15652;K15657;K15731;K15735;K15786;K15924;K15987;K16014;K16191;K16212;K16233;K16279;K16322;K16328;K16556;K16559;K16650;K16703;K16949;K17069;K17205;K17206;K17207;K17241;K17500;K17505;K17529;K17680;K17760;K17908;K17922;K18135;K18218;K18334;K18371;K18434;K18676;K18786;K18923;K18982;K18991;K19057;K19075;K19088;K19091;K19120;K19122;K19138;K19155;K19157;K19167;K19174;K19369;K19425;K19510;K19609;K19610;K19641;K19661;K19714;K19775;K19872;K20252;K20267;K20304;K20326;K20344;K20345;K20689;K20790;K20846;K20850;K21003;K21054;K21403;K21424;K21602;K21619;K21622;K21636;K21755;K21760;K21802;K21909;K21948;K21973;K22014;K22067;K22152;K22209;K22253;K22345;K22387;K22457;K22550;K22601;K23015;K23061;K23062;K23063;K23064;K23222;K23247;K23332;K23352;K23374;K23548;K23549;K24074;K24101
Module 09	K00066;K00126;K00166;K00285;K00624;K00635;K00673;K00717;K00768;K00835;K00840;K00882;K00986;K01008;K01070;K01150;K01197;K01275;K01374;K01617;K01795;K01959;K02024;K02071;K02072;K02073;K02099;K02192;K02351;K02362;K02377;K02379;K02395;K02396;K02399;K02400;K02413;K02475;K02647;K02659;K02660;K02661;K02770;K02824;K02901;K02949;K03415;K03451;K03669;K03670;K03674;K03814;K03896;K03939;K04073;K04361;K04478； K05297;K05375;K05548;K05787;K05832;K05833;K05845;K05851;K05853;K06075;K06159;K06596;K06597;K06598;K06599;K06720;K06857;K06992;K07004;K07084;K07168;K07337;K07518;K07555;K07639;K07661;K07807;K08064;K08357;K08359;K08869;K08901;K08902;K08964;K09251;K09516;K09626;K09699;K09786;K09947;K10022;K10023;K10024;K10025;K10036;K10037;K10038;K10109;K10110;K10217;K10412;K10531;K10680;K10743;K10829;K10925;K10927;K10942;K11070;K11258;K11731;K11891;K11893;K11894;K11895;K11896;K11900;K11901;K11904;K11905;K11906;K11907;K11908;K11909;K11910;K12276;K12278;K12279;K12280;K12281;K12284;K12285;K12286;K12287;K12304;K12382;K12501;K12946;K12948;K12977;K13255;K13356;K13485;K13695;K13700;K13771;K13776;K13777;K13778;K13954;K13979;K14162;K14301;K14347;K14674;K14980;K15045;K15191;K15373;K15758;K16011;K16077;K16242;K16243;K16244;K16245;K16246;K16249;K16562;K16912;K16953;K16961;K16962;K16963;K17424;K17540;K17578;K17805;K18076;K18291;K18300;K18710;K18916;K18998;K19002;K19068;K19168;K19290;K19291;K19292;K19293;K19295;K19296;K19577;K19585;K19586;K19621;K19623;K19664;K19694;K19978;K20003;K20185;K20280;K20303;K20543;K20798;K20971;K20973;K20975;K21006;K21007;K21008;K21009;K21010;K21011;K21012;K21023;K21024;K21025;K21064;K21084;K21088;K21134;K21137;K21218;K21323;K21324;K21738;K21748;K21866;K22105;K22477;K22548;K22758;K22770;K22885;K23127;K23227;K23228;K23547;K23978;K23993

Module 10	K00023;K00050;K00058;K00074;K00077;K00088;K00099;K00104;K00167;K00169;K00170;K00245;K00246;K00249;K00252;K00276;K00340;K00344;K00366;K00390;K00459;K00507;K00548;K00565;K00567;K00568;K00600;K00604;K00609;K00611;K00615;K00626;K00652;K00666;K00681;K00759;K00762;K00763;K00764;K00765;K00789;K00793;K00794;K00799;K00826;K00858;K00869;K00872;K00891;K00903;K00928;K00939;K00941;K00943;K00945;K00946;K00948;K00950;K00954;K00955;K00992;K00995;K01082;K01095;K01133;K01159;K01240;K01259;K01265;K01321;K01336;K01372;K01441;K01495;K01515;K01523;K01561;K01563;K01588;K01591;K01609;K01644;K01647;K01652;K01653;K01664;K01684;K01689;K01692;K01693;K01695;K01696;K01698;K01702;K01703;K01704;K01749;K01754;K01755;K01766;K01770;K01776;K01802;K01803;K01807;K01845;K01854;K01866;K01883;K01885;K01889;K01895;K01897;K01902;K01912;K01920;K01921;K01923;K01933;K01935;K01940;K01950;K01951;K01956;K01970;K02003;K02004;K02009;K02027;K02077;K02193;K02194;K02195;K02200;K02203;K02218;K02221;K02224;K02227;K02232;K02237;K02293;K02315;K02330;K02392;K02394;K02397;K02398;K02416;K02419;K02427;K02434;K02453;K02454;K02455;K02456;K02457;K02458;K02459;K02460;K02461;K02474;K02483;K02488;K02500;K02501;K02523;K02528;K02548;K02563;K02564;K02567;K02568;K02570;K02573;K02601;K02650;K02652;K02654;K02662;K02669;K02670;K02671;K02674;K02806;K02863;K02864;K02867;K02876;K02881;K02890;K02892;K02895;K02906;K02916;K02931;K02933;K02948;K02961;K02965;K02967;K02982;K02988;K02994;K03011;K03013;K03017;K03076;K03106;K03110;K03113;K03116;K03117;K03147;K03151;K03182;K03183;K03186;K03187;K03188;K03189;K03272;K03283;K03306;K03308;K03320;K03365;K03424;K03434;K03455;K03469;K03517;K03520;K03524;K03560;K03571;K03594;K03639;K03642;K03643;K03665;K03666;K03684;K03686;K03687;K03704;K03705;K03717;K03722;K03733;K03743;K03753;K03769;K03789;K03821;K03919;K03924;K03929;K03932;K04046;K04061;K04085;K04487;K04488;K04748;K04752;K04755;K04760;K04763;K04765;K04771;K05346;K05516;K05524;K05636;K05785;K05792;K05939;K05970;K06015;K06137;K06139;K06199;K06213;K06269;K06287;K06346;K06442;K06641;K06689;K06920;K06942;K06950;K06953;K06958;K06985;K07003;K07023;K07028;K07038;K07080;K07100;K07102;K07107;K07114;K07126;K07154;K07161;K07171;K07234;K07259;K07266;K07279;K07280;K07289;K07304;K07305;K07315;K07323;K07390;K07396;K07397;K07399;K07457;K07460;K07479;K07560;K07566;K07648;K07659;K07735;K07939;K07967;K08084;K08296;K08304;K08306;K08341;K08720;K08900;K08991;K08997;K09003;K09458;K09512;K09540;K09582;K09585;K09648;K09696;K09697;K09709;K09774;K09781;K09804;K09808;K09810;K09815;K09817;K09833;K09862;K09888;K09903;K09930;K09948;K09968;K09986;K10020;K10215;K10441;K10552;K10559;K10669;K10676;K10716;K10773;K10778;K10806;K11003;K11065;K11175;K11209;K11214;K11253;K11610;K11784;K12267;K12339;K12659;K12997;K13005;K13185;K13283;K13292;K13412;K13472;K13507;K13529;K13533;K13574;K13639;K13770;K13998;K14003;K14016;K14055;K14170;K14260;K14261;K14262;K14589;K14597;K14759;K14986;K14987;K15066;K15078;K15109;K15258;K15371;K15512;K15513;K15515;K15539;K15650;K15745;K15773;K15866;K15914;K16050;K16074;K16135;K16482;K16515;K16566;K16599;K16602;K16603;K16868;K16898;K16915;K17199;K17226;K17227;K17230;K17285;K17472;K17716;K17865;K18068;K18136;K18285;K18346;K18369;K18431;K18586;K18681;K18828;K18829;K18901;K18979;K19145;K19268;K19338;K19944;K20249;K20327;K20417;K20534;K20553;K20793;K20978;K21159;K21303;K21903;K21959;K21970;K22379;K22855;K22969;K23094;K23246;K23256;K23269;K23440;K23481;K23995;K23997
Module 11	K00881;K02732;K02900;K03016;K03510;K03512;K05677;K05931;K06335;K07870;K07933;K08202;K08339;K08494;K08517;K08808;K08873;K09571;K10084;K10703;K10878;K10994;K11971;K12195;K12795;K12882;K13342;K13513;K13635;K13752;K13753;K14620;K14787;K15719;K16274;K17807;K18764;K18806;K19059;K19382;K19921;K22763;K23514

Module 12	K00009;K00025;K00064;K00137;K00146;K00206;K00208;K00220;K00227;K00271;K00302;K00303;K00304;K00305;K00317;K00332;K00334;K00335;K00355;K00364;K00394;K00395;K00406;K00410;K00458;K00463;K00465;K00471;K00472;K00473;K00480;K00510;K00518;K00537;K00544;K00558;K00575;K00613;K00700;K00709;K00733;K00734;K00737;K00750;K00757;K00805;K00822;K00837;K00851;K00861;K00887;K00893;K01097;K01100;K01104;K01117;K01126;K01208;K01214;K01239;K01253;K01294;K01307;K01308;K01324;K01338;K01354;K01358;K01365;K01375;K01392;K01393;K01421;K01423;K01425;K01433;K01446;K01453;K01480;K01489;K01492;K01520;K01581;K01594;K01598;K01635;K01668;K01679;K01708;K01711;K01713;K01720;K01751;K01760;K01771;K01787;K01809;K01829;K01834;K01836;K01837;K01932;K01949;K02000;K02001;K02002;K02005;K02007;K02013;K02015;K02048;K02054;K02082;K02173;K02196;K02208;K02327;K02422;K02438;K02507;K02508;K02515;K02538;K02575;K02638;K02677;K02703;K02706;K02760;K02761;K02781;K02782;K02783;K02798;K02800;K02841;K02848;K02857;K02862;K02888;K02927;K02959;K03006;K03007;K03010;K03015;K03021;K03027;K03093;K03145;K03148;K03164;K03235;K03257;K03259;K03275;K03276;K03279;K03297;K03298;K03307;K03382;K03412;K03442;K03453;K03457;K03476;K03488;K03491;K03521;K03522;K03546;K03550;K03605;K03696;K03711;K03727;K03783;K03832;K03837;K03852;K03887;K03888;K03955;K04017;K04077;K04078;K04333;K04338;K04554;K04756;K04835;K05359;K05369;K05371;K05575;K05595;K05605;K05662;K05782;K05818;K05937;K05997;K06058;K06101;K06130;K06134;K06142;K06195;K06208;K06214;K06218;K06225;K06284;K06373;K06374;K06381;K06413;K06726;K06793;K06794;K06795;K06890;K06900;K06901;K06903;K06907;K06911;K06946;K06971;K06979;K06993;K06995;K06999;K07001;K07018;K07019;K07037;K07067;K07069;K07110;K07117;K07149;K07150;K07177;K07217;K07239;K07246;K07271;K07336;K07374;K07406;K07465;K07505;K07516;K07553;K07651;K07653;K07682;K07695;K07812;K07814;K07816;K07889;K07891;K07904;K07966;K07969;K07994;K08056;K08155;K08160;K08167;K08324;K08353;K08384;K08479;K08589;K08640;K08679;K08776;K08785;K08909;K08957;K08959;K09250;K09272;K09474;K09483;K09507;K09510;K09511;K09514;K09580;K09633;K09684;K09706;K09747;K09762;K09776;K09800;K09882;K09910;K09922;K09923;K09937;K09949;K09959;K09969;K09987;K10001;K10007;K10008;K10018;K10046;K10108;K10201;K10208;K10240;K10241;K10242;K10250;K10261;K10393;K10435;K10500;K10534;K10549;K10585;K10586;K10596;K10636;K10656;K10667;K10747;K10754;K10755;K10756;K10801;K10807;K10808;K10831;K10906;K10934;K10981;K11009;K11069;K11072;K11157;K11163;K11295;K11296;K11381;K11439;K11531;K11532;K11601;K11602;K11779;K11833;K11842;K11843;K11924;K11941;K11955;K11956;K11957;K11958;K11969;K11982;K11985;K12163;K12225;K12244;K12253;K12254;K12256;K12263;K12282;K12429;K12453;K12503;K12542;K12543;K12549;K12598;K12599;K12608;K12613;K12618;K12619;K12663;K12885;K12952;K12979;K13063;K13256;K13281;K13309;K13487;K13491;K13524;K13566;K13581;K13583;K13598;K13599;K13609;K13640;K13645;K13647;K13656;K13665;K13668;K13690;K13915;K13950;K13984;K13985;K14061;K14153;K14156;K14286;K14446;K14519;K14590;K14688;K14731;K14758;K14761;K14781;K15012;K15084;K15100;K15104;K15173;K15206;K15223;K15226;K15239;K15260;K15268;K15327;K15331;K15349;K15372;K15383;K15404;K15409;K15503;K15509;K15510;K15521;K15525;K15530;K15551;K15552;K15637;K15654;K15655;K15663;K15702;K15737;K15771;K15772;K15792;K15895;K15973;K16025;K16095;K16119;K16124;K16130;K16132;K16134;K16214;K16264;K16271;K16282;K16290;K16292;K16312;K16342;K16365;K16370;K16371;K16384;K16393;K16437;K16516;K16582;K16627;K16652;K16816;K16838;K16914;K17208;K17209;K17217;K17234;K17235;K17248;K17329;K17330;K17331;K17486;K17497;K17509;K17641;K17675;K17738;K17783;K17818;K17871;K17938;K18104;K18204;K18230;K18277;K18302;K18303;K18342;K18382;K18453;K18455;K18537;K18554;K18555;K18575;K18704;K18814;K18817;K18833;K18844;K18886;K18917;K18934;K18955;K19028;K19038;K19040;K19041;K19044;K19069;K19156;K19336;K19465;K19573;K19591;K19622;K19703;K19770;K19778;K19784;K19956;K19969;K19972;K19976;K20285;K20485;K20490;K20883;K20902;K20927;K21001;K21033;K21060;K21061;K21196;K21214;K21248;K21249;K21252;K21273;K21308;K21401;K21402;K21418;K21467;K21471;K21480;K21493;K21585;K21617;K21687;K21699;K21706;K21711;K21801;K21831;K21908;K21953;K21966;K21968;K22015;K22025;K22063;K22072;K22081;K22082;K22083;K22084;K22116;K22140;K22185;K22215;K22216;K22230;K22250;K22282;K22307;K22308;K22311;K22319;K22342;K22350;K22397;K22406;K22407;K22459;K22460;K22549;K22646;K22651;K22697;K22721;K22909;K22935;K23014;K23036;K23059;K23076;K23083;K23147;K23197;K23334;K23385;K23463;K23492;K23516;K23546;K23683;K23786;K23788;K23871;K23874;K23980;K23987;K23991;K23999;K24091

Module 13	K00005;K00024;K00032;K00052;K00069;K00098;K00118;K00216;K00240;K00293;K00453;K00469;K00476;K00484;K00515;K00559;K00646;K00663;K00691;K00692;K00702;K00738;K00752;K00767;K00771;K00839;K00875;K00892;K00898;K00907;K00908;K00916;K00934;K00981;K00991;K00998;K01034;K01051;K01069;K01119;K01181;K01191;K01196;K01202;K01212;K01226;K01235;K01245;K01252;K01387;K01406;K01451;K01462;K01467;K01475;K01528;K01646;K01648;K01707;K01730;K01736;K01761;K01788;K01813;K01814;K01817;K01931;K02043;K02081;K02089;K02132;K02190;K02211;K02359;K02364;K02414;K02442;K02479;K02493;K02526;K02580;K02588;K02590;K02593;K02679;K02773;K02774;K02775;K02777;K02791;K02804;K02810;K02852;K02855;K02898;K02914;K02925;K02952;K02998;K03094;K03097;K03210;K03225;K03246;K03248;K03260;K03262;K03267;K03291;K03408;K03435;K03452;K03470;K03484;K03496;K03532;K03533;K03577;K03604;K03645;K03647;K03712;K03763;K03765;K03776;K03825;K03827;K03868;K03870;K03894;K03895;K03921;K03931;K04013;K04036;K04067;K04087;K04097;K04335;K04337;K04348;K04351;K04430;K04507;K04555;K04618;K04630;K04718;K04725;K04775;K04852;K05039;K05311;K05341;K05351;K05355;K05373;K05577;K05596;K05619;K05761;K05766;K05811;K05857;K05880;K05961;K06080;K06113;K06120;K06121;K06141;K06155;K06160;K06184;K06191;K06209;K06221;K06222;K06348;K06370;K06377;K06441;K06515;K06605;K06620;K06627;K06645;K06704;K06834;K06851;K06859;K06918;K06933;K07002;K07017;K07051;K07094;K07132;K07181;K07186;K07192;K07225;K07229;K07242;K07272;K07282;K07297;K07314;K07322;K07499;K07513;K07687;K07704;K07706;K07718;K07752;K07760;K07770;K07791;K07792;K07811;K07874;K07890;K07893;K07902;K07922;K07936;K07950;K07978;K08143;K08156;K08175;K08222;K08337;K08342;K08515;K08516;K08621;K08659;K08664;K08711;K08745;K08766;K08767;K08790;K08796;K08799;K08829;K08832;K08835;K08847;K08860;K08867;K08958;K08960;K09021;K09023;K09024;K09155;K09161;K09186;K09265;K09485;K09503;K09505;K09508;K09552;K09583;K09632;K09646;K09688;K09689;K09749;K09890;K09907;K09914;K09965;K10019;K10021;K10094;K10107;K10117;K10118;K10119;K10188;K10192;K10212;K10278;K10403;K10537;K10539;K10540;K10541;K10542;K10544;K10545;K10546;K10547;K10548;K10555;K10556;K10560;K10573;K10575;K10576;K10582;K10635;K10733;K10748;K10771;K10774;K10814;K10815;K10816;K10820;K10859;K10860;K10877;K10900;K10909;K11021;K11031;K11079;K11109;K11121;K11150;K11165;K11185;K11216;K11407;K11427;K11441;K11475;K11540;K11541;K11614;K11615;K11617;K11641;K11703;K11714;K11746;K11788;K11885;K11925;K11926;K11929;K11933;K11975;K11995;K12037;K12047;K12049;K12053;K12070;K12166;K12183;K12239;K12322;K12370;K12460;K12476;K12516;K12579;K12582;K12896;K13001;K13161;K13208;K13243;K13273;K13294;K13346;K13497;K13526;K13537;K13602;K13611;K13612;K13614;K13615;K13636;K13643;K13646;K13666;K13683;K13684;K13745;K13797;K13876;K13907;K13917;K13996;K14053;K14057;K14062;K14082;K14161;K14163;K14189;K14270;K14426;K14455;K14640;K14644;K14655;K14677;K14681;K14762;K14850;K14982;K15014;K15025;K15042;K15064;K15083;K15105;K15112;K15114;K15115;K15231;K15269;K15311;K15312;K15313;K15314;K15632;K15656;K15660;K15661;K15662;K15667;K15710;K15711;K15923;K16075;K16087;K16088;K16125;K16146;K16210;K16248;K16273;K16285;K16289;K16315;K16466;K16507;K16509;K16552;K16555;K16558;K16568;K16710;K16840;K16918;K16924;K17047;K17065;K17105;K17202;K17203;K17204;K17213;K17214;K17215;K17237;K17238;K17239;K17240;K17246;K17339;K17362;K17499;K17501;K17582;K17584;K17615;K17655;K17723;K17900;K17906;K18005;K18012;K18033;K18041;K18059;K18078;K18118;K18131;K18197;K18213;K18220;K18221;K18298;K18299;K18330;K18331;K18337;K18349;K18408;K18416;K18533;K18551;K18578;K18581;K18674;K18675;K18741;K18754;K18765;K18776;K18783;K18846;K18904;K18910;K18924;K18926;K18996;K19005;K19037;K19045;K19046;K19048;K19065;K19113;K19117;K19123;K19124;K19125;K19126;K19147;K19173;K19236;K19354;K19375;K19386;K19412;K19422;K19423;K19426;K19430;K19545;K19575;K19600;K19640;K19691;K19697;K19721;K19744;K19755;K19852;K19870;K19954;K20098;K20103;K20118;K20216;K20235;K20263;K20461;K20472;K20541;K20600;K20726;K20756;K20918;K20919;K20950;K20965;K20977;K21062;K21085;K21130;K21278;K21362;K21394;K21428;K21429;K21507;K21556;K21634;K21696;K21698;K21701;K21777;K21778;K21779;K21780;K21781;K21782;K21783;K21784;K21785;K21787;K21832;K21864;K21885;K21889;K21890;K21910;K21911;K21965;K22104;K22109;K22110;K22129;K22268;K22293;K22470;K22539;K22551;K22927;K22933;K22991;K22994;K23169;K23170;K23244;K23245;K23248;K23262;K23439;K23508;K23509;K23564;K23675;K23692;K23779;K23988;K24004;K24082;K24087

Module 14	K00001;K00010;K00015;K00027;K00030;K00034;K00045;K00054;K00059;K00068;K00073;K00087;K00090;K00091;K00096;K00114;K00117;K00120;K00131;K00135;K00148;K00150;K00172;K00174;K00175;K00184;K00186;K00188;K00214;K00217;K00221;K00247;K00260;K00277;K00282;K00283;K00289;K00298;K00299;K00301;K00306;K00313;K00318;K00320;K00331;K00359;K00368;K00375;K00389;K00392;K00407;K00450;K00451;K00454;K00464;K00483;K00496;K00528;K00542;K00545;K00547;K00549;K00555;K00561;K00573;K00577;K00596;K00598;K00601;K00603;K00610;K00621;K00634;K00662;K00696;K00697;K00710;K00721;K00754;K00761;K00782;K00788;K00798;K00803;K00808;K00823;K00827;K00836;K00856;K00857;K00860;K00883;K00885;K00926;K00947;K00949;K00952;K00958;K00962;K00966;K00978;K00980;K00983;K00996;K01001;K01002;K01003;K01009;K01011;K01012;K01014;K01015;K01017;K01025;K01028;K01029;K01039;K01040;K01042;K01044;K01053;K01054;K01055;K01061;K01087;K01096;K01144;K01151;K01166;K01170;K01173;K01174;K01185;K01210;K01222;K01233;K01236;K01241;K01244;K01246;K01295;K01301;K01303;K01342;K01346;K01397;K01413;K01430;K01432;K01442;K01455;K01457;K01458;K01459;K01460;K01463;K01464;K01469;K01470;K01473;K01474;K01478;K01493;K01496;K01501;K01512;K01518;K01519;K01535;K01537;K01555;K01560;K01562;K01569;K01576;K01577;K01582;K01595;K01596;K01604;K01607;K01612;K01622;K01631;K01639;K01641;K01654;K01674;K01677;K01683;K01694;K01706;K01709;K01715;K01721;K01724;K01726;K01733;K01737;K01738;K01743;K01744;K01752;K01759;K01768;K01777;K01781;K01784;K01793;K01801;K01826;K01841;K01843;K01848;K01849;K01856;K01858;K01880;K01884;K01896;K01899;K01953;K01966;K01971;K01974;K01975;K01990;K01992;K01994;K02014;K02016;K02021;K02022;K02025;K02026;K02029;K02030;K02032;K02034;K02035;K02041;K02042;K02044;K02046;K02047;K02049;K02050;K02051;K02056;K02057;K02058;K02065;K02068;K02069;K02079;K02083;K02103;K02117;K02118;K02119;K02120;K02121;K02122;K02123;K02124;K02128;K02172;K02188;K02189;K02191;K02201;K02206;K02217;K02245;K02251;K02274;K02275;K02276;K02277;K02278;K02279;K02280;K02281;K02282;K02292;K02295;K02303;K02304;K02320;K02322;K02323;K02334;K02380;K02386;K02387;K02388;K02389;K02391;K02393;K02401;K02402;K02403;K02404;K02408;K02410;K02411;K02415;K02417;K02418;K02420;K02421;K02423;K02426;K02431;K02433;K02435;K02437;K02445;K02450;K02472;K02473;K02485;K02490;K02492;K02503;K02518;K02540;K02549;K02550;K02569;K02584;K02603;K02615;K02626;K02635;K02651;K02668;K02683;K02686;K02688;K02727;K02744;K02745;K02784;K02793;K02795;K02796;K02823;K02849;K02858;K02866;K02869;K02877;K02883;K02885;K02889;K02893;K02896;K02899;K02902;K02904;K02907;K02908;K02910;K02912;K02915;K02919;K02921;K02930;K02936;K02962;K02966;K02975;K02976;K02984;K02987;K02991;K02995;K02996;K03040;K03041;K03046;K03047;K03049;K03057;K03058;K03059;K03088;K03105;K03119;K03120;K03124;K03130;K03135;K03136;K03149;K03154;K03163;K03166;K03167;K03208;K03223;K03230;K03231;K03232;K03233;K03234;K03236;K03237;K03238;K03242;K03243;K03263;K03264;K03265;K03271;K03273;K03274;K03284;K03294;K03296;K03299;K03300;K03315;K03329;K03330;K03335;K03336;K03337;K03338;K03353;K03367;K03381;K03383;K03388;K03389;K03390;K03392;K03394;K03395;K03399;K03405;K03410;K03413;K03414;K03418;K03425;K03430;K03432;K03433;K03446;K03478;K03518;K03519;K03538;K03540;K03543;K03563;K03564;K03567;K03574;K03585;K03602;K03622;K03626;K03636;K03651;K03652;K03660;K03675;K03679;K03680;K03699;K03707;K03718;K03726;K03737;K03738;K03739;K03741;K03744;K03754;K03757;K03768;K03779;K03780;K03784;K03785;K03788;K03794;K03795;K03799;K03800;K03802;K03823;K03828;K03829;K03842;K03851;K03884;K03885;K03917;K03926;K03943;K04014;K04015;K04019;K04034;K04065;K04069;K04070;K04074;K04076;K04091;K04094;K04098;K04099;K04102;K04103;K04107;K04108;K04109;K04110;K04112;K04113;K04114;K04115;K04127;K04382;K04479;K04482;K04483;K04496;K04550;K04561;K04564;K04565;K04655;K04712;

	K04719;K04720;K04749;K04750;K04751;K04794;K04795;K04796;K04797;K04798;K04799;K04800;K05030;K05181;K05281;K05296;K05299;K05301;K05303;K05343;K05358;K05384;K05396;K05525;K05549;K05550;K05566;K05567;K05568;K05569;K05570;K05571;K05576;K05578;K05588;K05602;K05606;K05607;K05612;K05685;K05708;K0571;K02793;K02795;K02796;K02823;K02849;K02858;K02866;K02869;K02877;K02883;K02885;K02889;K02893;K02896;K02899;K02902;K02904;K02907;K02908;K02910;K02912;K02915;K02919;K02921;K02930;K02936;K02962;K02966;K02975;K02976;K02984;K02987;K02991;K02995;K02996;K03040;K03041;K03046;K03047;K03049;K03057;K03058;K03059;K03088;K03105;K03119;K03120;K03124;K03130;K03135;K03136;K03149;K03154;K03163;K03166;K03167;K03208;K03223;K03230;K03231;K03232;K03233;K03234;K03236;K03237;K03238;K03242;K03243;K03263;K03264;K03265;K03271;K03273;K03274;K03284;K03294;K03296;K03299;K03300;K03315;K03329;K03330;K03335;K03336;K03337;K03338;K03353;K03367;K03381;K03383;K03388;K03389;K03390;K03392;K03394;K03395;K03399;K03405;K03410;K03413;K03414;K03418;K03425;K03430;K03432;K03433;K03446;K03478;K03518;K03519;K03538;K03540;K03543;K03563;K03564;K03567;K03574;K03585;K03602;K03622;K03626;K03636;K03651;K03652;K03660;K03675;K03679;K03680;K03699;K03707;K03718;K03726;K03737;K03738;K03739;K03741;K03744;K03754;K03757;K03768;K03779;K03780;K03784;K03785;K03788;K03794;K03795;K03799;K03800;K03802;K03823;K03828;K03829;K03842;K03851;K03884;K03885;K03917;K03926;K03943;K04014;K04015;K04019;K04034;K04065;K04069;K04070;K04074;K04076;K04091;K04094;K04098;K04099;K04102;K04103;K04107;K04108;K04109;K04110;K04112;K04113;K04114;K04115;K04127;K04382;K04479;K04482;K04483;K04496;K04550;K04561;K04564;K04565;K04655;K04712;K04719;K04720;K04749;K04750;K04751;K04794;K04795;K04796;K04797;K04798;K04799;K04800;K05030;K05181;K05281;K05296;K05299;K05301;K05303;K05343;K05358;K05384;K05396;K05525;K05549;K05550;K05566;K05567;K05568;K05569;K05570;K05571;K05576;K05578;K05588;K05602;K05606;K05607;K05612;K05685;K05708;K05710;K05715;K05746;K05788;K05793;K05794;K05800;K05802;K05813;K05814;K05815;K05816;K05819;K05827;K05828;K05829;K05830;K05831;K05847;K05878;K05879;K05884;K05886;K05908;K05918;K05921;K05925;K05928;K05934;K05936;K05956;K05982;K06013;K06016;K06019;K06023;K06024;K06033;K06034;K06042;K06044;K06045;K06048;K06131;K06145;K06151;K06152;K06164;K06167;K06174;K06188;K06196;K06197;K06201;K06212;K06215;K06306;K06310;K06351;K06378;K06400;K06402;K06410;K06416;K06602;K06666;K06798;K06862;K06863;K06864;K06865;K06867;K06869;K06875;K06877;K06885;K06888;K06889;K06898;K06912;K06917;K06922;K06927;K06930;K06932;K06937;K06940;K06944;K06952;K06961;K06962;K06963;K06965;K06976;K06978;K06981;K06982;K06983;K06987;K06988;K06989;K06990;K06996;K06998;K07005;K07006;K07008;K07020;K07027;K07031;K07032;K07034;K07041;K07045;K07048;K07058;K07059;K07060;K07061;K07063;K07064;K07065;

	K07068;K07079;K07104;K07108;K07127;K07130;K07142;K07143;K07151;K07152;K07155;K07158;K07159;K07166;K07176;K07178;K07216;K07219;K07220;K07221;K07226;K07228;K07232;K07233;<br>K07238;K07240;K07254;K07257;K07270;K07286;K07290;K07300;K07301;K07306;K07307;K07325;K07329;K07330;K07331;K07332;K07333;K07334;K07335;K07339;K07341;K07342;K07343;K07344;<br>K07347;K07387;K07389;K07398;K07403;K07405;K07442;K07444;K07445;K07446;K07455;K07458;K07461;K07463;K07466;K07469;K07474;K07477;K07488;K07491;K07492;K07497;K07501;K07502;<br>K07507;K07508;K07512;K07535;K07536;K07537;K07538;K07539;K07544;K07546;K07549;K07558;K07559;K07561;K07571;K07572;K07573;K07575;K07577;K07581;K07583;K07584;K07588;K07649;<br>K07652;K07658;K07665;K07667;K07673;K07686;K07709;K07710;K07714;K07716;K07722;K07724;K07732;K07739;K07743;K07749;K07755;K07766;K07767;K07772;K07774;K07806;K07823;K07979;<br>K07991;K07997;K08096;K08097;K08099;K08153;K08161;K08176;K08188;K08223;K08234;K08279;K08295;K08303;K08315;K08318;K08320;K08321;K08344;K08352;K08354;K08355;K08356;K08358;<br>K08363;K08365;K08478;K08482;K08483;K08587;K08608;K08614;K08637;K08641;K08646;K08677;K08678;K08681;K08689;K08691;K08693;K08697;K08726;K08738;K08787;K08818;K08851;K08884;<br>K08963;K08966;K08967;K08972;K08975;K08979;K08982;K08984;K08990;K08995;K09000;K09002;K09005;K09012;K09013;K09014;K09018;K09019;K09020;K09022;K09118;K09120;K09121;K09122;<br>K09127;K09128;K09129;K09131;K09137;K09138;K09140;K09141;K09142;K09143;K09145;K09148;K09149;K09152;K09162;K09163;K09286;K09386;K09457;K09459;K09461;K09482;K09527;K09607;<br>K09608;K09611;K09640;K09667;K09681;K09690;K09691;K09692;K09693;K09707;K09711;K09716;K09721;K09722;K09723;K09726;K09735;K09736;K09738;K09759;K09763;K09764;K09766;K09771;<br>K09777;K09778;K09779;K09791;K09798;K09803;K09807;K09809;K09816;K09866;K09873;K09924;K09931;K09933;K09954;K09967;K09979;K09981;K09985;K09988;K09990;K09991;K09994;K10012;<br>K10026;K10047;K10111;K10112;K10191;K10222;K10227;K10228;K10229;K10238;K10253;K10255;K10324;K10325;K10407;K10439;K10440;K10532;K10535;K10553;K10554;K10565;K10566;K10580;<br>K10597;K10601;K10609;K10614;K10620;K10621;K10622;K10623;K10670;K10671;K10672;K10674;K10678;K10725;K10726;K10751;K10775;K10794;K10797;K10798;K10810;K10819;K10822;K10848;<br>K10854;K10908;K10913;K10914;K10915;K10917;K10921;K10926;K10947;K10951;K10956;K10977;K10979;K11068;K11073;K11074;K11076;K11078;K11102;K11105;K11106;K11127;K11131;K11141;<br>K11173;K11176;K11178;K11192;K11196;K11203;K11206;K11212;K11260;K11312;K11355;K11356;K11357;K11358;K11366;K11414;K11443;K11477;K11493;K11517;K11523;K11529;K11530;K11600;<br>K11620;K11621;K11646;K11693;K11717;K11747;K11751;K11780;K11781;K11785;K11787;K11903;K11913;K11921;K11928;K11932;K11935;K11936;K11937;K11945;K11946;K11948;

	K11954;K11994;K11997;K12035;K12055;K12132;K12143;K12196;K12198;K12234;K12255;K12261;K12283;K12373;K12409;K12410;K12448;K12449;K12502;K12507;K12511;K12527;K12532;K12537;K12538;K12541;K12584;K12589;K12685;K12800;K12944;K12960;K12973;K12976;K12984;K12988;K12992;K12995;K12999;K13004;K13006;K13010;K13013;K13014;K13015;K13018;K13019;K13020;K13028;K13037;K13052;K13060;K13061;K13069;K13075;K13236;K13237;K13280;K13290;K13307;K13327;K13419;K13483;K13503;K13525;K13527;K13541;K13571;K13582;K13584;K13587;K13588;K13589;K13590;K13592;K13593;K13607;K13626;K13632;K13677;K13678;K13687;K13688;K13706;K13713;K13719;K13722;K13748;K13787;K13796;K13798;K13799;K13816;K13858;K13932;K13953;K13993;K13995;K14063;K14080;K14113;K14127;K14152;K14165;K14166;K14257;K14259;K14333;K14358;K14387;K14388;K14415;K14445;K14451;K14459;K14475;K14491;K14534;K14554;K14558;K14564;K14568;K14574;K14578;K14581;K14584;K14588;K14591;K14596;K14647;K14654;K14656;K14660;K14672;K14727;K14728;K14729;K14733;K14749;K14750;K14751;K14855;K14940;K14974;K15003;K15011;K15016;K15019;K15022;K15023;K15024;K15052;K15061;K15062;K15065;K15125;K15241;K15270;K15357;K15358;K15384;K15429;K15449;K15468;K15497;K15504;K15554;K15583;K15634;K15635;K15669;K15736;K15750;K15751;K15756;K15760;K15761;K15762;K15763;K15764;K15765;K15780;K15856;K15864;K15865;K15888;K15894;K15897;K15974;K15977;K15980;K16017;K16033;K16035;K16044;K16047;K16133;K16147;K16148;K16153;K16159;K16167;K16202;K16238;K16254;K16255;K16268;K16293;K16299;K16300;K16302;K16304;K16306;K16317;K16319;K16320;K16426;K16431;K16436;K16531;K16634;K16651;K16653;K16704;K16842;K16843;K16850;K16856;K16869;K16871;K16873;K16874;K16875;K16899;K16901;K16905;K16906;K16907;K16922;K16928;K16968;K16969;K17064;K17080;K17104;K17228;K17242;K17243;K17249;K17266;K17292;K17322;K17323;K17325;K17363;K17364;K17382;K17398;K17462;K17468;K17530;K17535;K17560;K17624;K17662;K17722;K17724;K17749;K17752;K17754;K17758;K17759;K17762;K17781;K17810;K17828;K17830;K17832;K17837;K17839;K17872;K17884;K17893;K17898;K17899;K17947;K17950;K17981;K17993;K17994;K17996;K18014;K18016;K18023;K18028;K18029;K18030;K18057;K18067;K18074;K18087;K18088;K18092;K18096;K18101;K18123;K18130;K18162;K18163;K18166;K18209;K18210;K18229;K18232;K18233;K18235;K18237;K18240;K18242;K18243;K18244;K18258;K18290;K18292;K18293;K18295;K18297;K18312;K18313;K18336;K18361;K18362;K18363;K18384;K18398;K18429;K18430;K18471;K18479;K18480;K18532;K18534;K18556;K18567;K18587;K18593;K18594;K18601;K18602;K18603;K18604;K18605;K18611;K18678;K18700;K18702;K18703;K18770;K18779;K18802;K18816;K18824;K18825;K18827;K18843;K18855;K18882;K18896;K18897;K18900;K18905;K18911;K18912;K18914;K18918;K18928;K18929;K18931;K18997;K19032;K19055;K19058;K19064;K19114;K19116;K19141;K19189;K19200;K19221;K19243;K19244;K19265;K19278;K19285;K19286;K19294;K19302;K19340;K19341;K19343;K19344;K19345;K19360;K19367;K19405;K19411;K19413;K19429;K19509;K19517;K19548;K19560;K19576;K19592;K19593;K19594;K19620;K19647;K19660;K19670;K19696;K19702;K19707;K19712;K19715;K19732;K19761;K19794;K19795;K19803;K19813;K19856;K19874;K19966;K19967;K20037;K20038;K20148;K20215;K20218;K20276;K20333;K20428;K20446;K20451;K20454;K20459;K20523;K20527;K20532;K20716;K20762;K20807;K20814;K20938;K20941;K20942;K20962;K20976;K20989;K20995;K21014;K21027;K21028;K21029;K21039;K21052;K21071;K21087;K21119;K21135;K21140;K21142;K21162;K21185;K21195;K21210;K21219;K21232;K21285;K21306;K21310;K21331;K21332;K21337;K21344;K21377;K21399;K21400;K21416;K21417;K21495;K21498;K21512;K21514;K21563;K21567;K21577;K21579;K21584;K21606;K21647;K21672;K21677;K21678;K21679;K21700;K21721;K21723;K21731;K21741;K21759;K21814;K21833;K21884;K21898;K21902;K21929;K21947;K21972;K21990;K22010;K22027;K22044;K22073;K22085;K22087;K22099;K22130;K22133;K22135;K22169;K22205;K22210;K22213;K22223;K22224;K22225;K22226;K22231;K22270;K22278;K22296;K22297;K22299;K22301;K22305;K22317;K22322;K22348;K22349;K22373;K22389;K22396;K22405;K22447;K22451;K22452;K22473;K22481;K22482;K22486;K22491;K22579;K22585;K22696;K22699;K22704;K22705;K22712;K22715;K22732;K22734;K22736;K22737;K22818;K22837;K22843;K22844;K22848;K22881;K22894;K22907;K22955;K22958;K22960;K22970;K23010;K23020;K23021;K23078;K23086;K23107;K23144;K23148;K23163;K23167;K23184;K23237;K23255;K23259;K23264;K23265;K23275;K23300;K23349;K23356;K23359;K23370;K23376;K23398;K23462;K23464;K23499;K23518;K23528;K23550;K23555;K23556;K23557;K23684;K23725;K23743;K23755;K23773;K23876;K23886;K23911;K23976;K23977;K23992;K23998;K24003;K24012;K24028;K24071;K24105

Module 15	K00086;K00218;K00272;K00488;K00630;K00811;K00818;K00910;K00999;K01021;K01060;K01068;K01396;K01436;K01513;K01763;K01909;K02080;K02366;K02505;K02589;K02831;K03095;K03112;K03162;K03191;K03219;K03222;K03224;K03226;K03227;K03228;K03229;K03309;K03429;K03464;K03633;K04030;K04058;K04428;K04497;K04772;K05306;K05352;K05518;K05535;K05574;K05586;K05593;K05600;K05760;K05824;K05929;K05941;K06228;K06233;K06887;K07128;K07148;K07468;K07495;K07543;K07550;K07570;K07670;K07680;K07688;K07723;K07750;K07783;K07809;K07996;K08002;K08004;K08081;K08178;K08246;K08604;K08616;K08687;K08713;K08723;K08765;K08797;K08800;K08906;K09477;K09565;K09612;K09669;K09671;K09700;K09714;K09787;K09828;K09996;K10011;K10017;K10254;K10526;K10673;K10811;K11154;K11395;K11841;K12137;K12140;K12141;K12226;K12374;K12442;K12761;K13002;K13065;K13086;K13311;K13717;K13853;K13856;K13889;K13891;K14154;K14251;K14330;K14427;K14585;K14746;K14748;K15001;K15020;K15060;K15352;K15487;K15502;K15523;K15549;K15641;K15681;K15752;K15754;K15755;K16043;K16164;K16179;K16203;K16217;K16444;K16789;K16849;K16860;K16964;K16965;K16966;K17060;K17077;K17299;K17311;K17312;K17313;K17623;K17725;K17739;K17811;K17877;K18021;K18022;K18073;K18089;K18144;K18236;K18314;K18417;K18424;K18475;K18482;K18546;K18566;K18670;K18693;K19188;K19428;K19595;K19658;K19731;K19975;K20053;K20171;K20200;K20202;K20268;K20334;K20412;K20448;K20772;K20816;K20872;K20895;K20987;K20998;K21021;K21053;K21115;K21172;K21255;K21256;K21272;K21681;K21912;K22100;K22101;K22478;K22747;K22808;K22874;K22920;K22952;K23004;K23181;K23448;K23685;K23774;K23790;K24085
Module 16	K01750;K02298;K03465;K03815;K03834;K04984;K05340;K05916;K06217;K06921;K07484;K09698;K12202;K12203;K12204;K12205;K12206;K12207;K12208;K12209;K12210;K12211;K12212;K12213;K12214;K12216;K12217;K12218;K12219;K12221;K12222;K12223;K12224;K16925;K18119;K19742;K20265;K23060
Module 17	K00049;K00196;K00200;K00201;K00202;K00203;K00213;K00222;K00300;K00485;K00672;K00801;K01090;K01281;K01499;K01500;K01506;K01630;K01678;K01903;K02424;K02571;K02637;K02653;K02667;K02672;K02725;K03044;K03316;K03319;K03416;K03649;K03793;K03810;K03830;K03855;K04480;K05275;K05556;K05580;K05597;K05966;K06059;K06132;K06583;K06718;K06897;K06913;K06914;K06984;K07072;K07099;K07103;K07144;K07244;K07264;K07357;K07715;K07728;K07731;K08003;K08082;K08137;K08169;K08983;K09119;K09154;K09384;K09725;K09730;K09733;K09880;K09932;K09973;K09983;K10041;K10338;K10529;K10702;K10713;K10714;K10715;K10742;K10978;K11440;K11444;K11687;K11912;K12536;K13007;K13317;K13380;K13490;K13831;K14676;K15234;K15734;K15740;K15757;K15896;K15904;K15981;K16200;K16395;K16904;K17910;K17942;K18608;K18610;K18612;K19003;K19052;K20049;K20332;K21019;K21211;K21440;K21613;K22026;K22168;K22298;K22750;K22879;K22895;K23054;K23075;K23271;K23424
Module 18	K00124;K00127;K00263;K00520;K01608;K01816;K02106;K02226;K02233;K02390;K02412;K02556;K02557;K03556;K04562;K05772;K05773;K06140;K06923;K07136;K07250;K08083;K08988;K09181;K09978;K10216;K10943;K13895;K16876;K16877;K16878;K16879;K16880;K20447;K20906;K20907;K22424;K23150;K23777

Module 19	K00288;K00436;K00810;K00923;K00984;K01590;K01822;K03516;K03623;K04377;K05100;K05122;K05286;K05653;K05889;K05994;K05996;K06072;K06683;K06871;K06936;K07049;K07109;K07129;K07180;K07557;K07579;K07644;K07719;K07899;K07976;K08174;K08588;K08635;K08683;K08685;K08804;K08831;K08987;K09628;K10634;K10863;K11166;K11183;K11210;K11228;K11711;K11729;K12071;K12343;K12350;K12351;K12686;K13651;K13655;K13703;K14085;K14392;K14983;K15047;K15067;K16051;K16172;K16269;K16368;K16557;K16639;K16646;K16698;K17474;K17861;K17891;K18006;K18007;K18069;K18306;K18307;K18476;K18589;K18795;K18967;K18974;K18975;K19301;K19519;K19626;K19668;K19705;K20011;K20032;K20169;K20429;K20780;K20796;K20873;K20896;K20928;K21163;K21867;K22249;K22353;K22982;K23034;K23270;K23975
